# In silico genetic robustness analysis of microRNA secondary structures: potential evidence of congruent evolution in microRNA

**DOI:** 10.1186/1471-2148-7-223

**Published:** 2007-11-13

**Authors:** Wenjie Shu, Xiaochen Bo, Ming Ni, Zhiqiang Zheng, Shengqi Wang

**Affiliations:** 1 Beijing Institute of Radiation Medicine, Beijing 100850, China; 2 College of Electro-Mechanic and Automation, National University of Defense Technology, Changsha, Hunan 410073, China

## Abstract

**Background:**

Robustness is a fundamental property of biological systems and is defined as the ability to maintain stable functioning in the face of various perturbations. Understanding how robustness has evolved has become one of the most attractive areas of research for evolutionary biologists, as it is still unclear whether genetic robustness evolved as a direct consequence of natural selection, as an intrinsic property of adaptations, or as congruent correlate of environment robustness. Recent studies have demonstrated that the stem-loop structures of microRNA (miRNA) are tolerant to some structural changes and show thermodynamic stability. We therefore hypothesize that genetic robustness may evolve as a correlated side effect of the evolution for environmental robustness.

**Results:**

We examine the robustness of 1,082 miRNA genes covering six species. Our data suggest the stem-loop structures of miRNA precursors exhibit a significantly higher level of genetic robustness, which goes beyond the intrinsic robustness of the stem-loop structure and is not a byproduct of the base composition bias. Furthermore, we demonstrate that the phenotype of miRNA buffers against genetic perturbations, and at the same time is also insensitive to environmental perturbations.

**Conclusion:**

The results suggest that the increased robustness of miRNA stem-loops may result from congruent evolution for environment robustness. Potential applications of our findings are also discussed.

## Background

Robustness, a fundamental and ubiquitously observed phenomenon in biological systems, is defined as the ability to maintain stable functioning in the face of various perturbations, and is characterized as genetic or environmental robustness, depending on whether the perturbations are inheritable or not [1]. Genetic robustness describes insensitivity of a phenotype facing genetic mutations, and the insensitivity to environmental factors is called environmental robustness. Phenotype robustness appears at various levels of biological systems, including gene expression, protein folding, metabolic flux, physiological homeostasis, development, and even organism fitness [2]. It is consequently not surprising that biologists have a long-standing interest in robustness, going back to Fisher's work on dominance [3-5], and to Waddington's developmental canalization research [6,7]. Hiroaki Kitano argues that the requirements for robustness and evolvability are similar, since robustness facilitates evolution and evolution favours robust traits [8]. A proper understanding of the origin of robustness in biological systems will catalyze our understanding of evolution [9].

The evolution of mechanism underlying the buffering of the phenotype against genetic and environmental influences has received much theoretical and experimental attention in recent years, yet the evolutionary origin of the observed robustness remains unresolved. Whether it is a consequence of natural selection or a nonadaptive correlated side-effect of other phenotypic traits is by and large unknown. A recent review article categorized the theories addressing the evolution of genetic robustness into three main classes: adaptive, intrinsic, and congruent [2]. The most straightforward explanation for the evolution of robustness, according to the Darwinian tradition, is adaptive robustness. In this scenario, almost all mutations lead to deviations from the optimum, and robustness is favored by natural selection. High mutation rates, large populations, and asexual reproduction generally favor the evolution of robustness [10,11]. Genetic robustness may also evolve simply because buffering is a necessary consequence of character adaptation; that is, robustness is a nonadaptive correlated side effect of the stabilizing selection acting on other traits [12]. Additionally, because environmental perturbations often have a higher frequency and stronger impact on fitness, they will serve as the driving force; that is, genetic robustness evolves as a correlated side-effect of the evolution for environmental robustness. This is an appealing hypothesis as there is no aspect of an organism that is inherently and persistently vulnerable to genetic but not environmental perturbations [12]. Support for this theory comes from a recent computational study of RNA secondary structure by Ancel and Fontana [13], who find that RNA shapes that are robust against environmental (thermodynamic) perturbations are also robust against mutational perturbations. Simplified modeling of protein structures suggests that a similar correlation between genetic robustness and thermodynamic stability might also exist for proteins [14-16]. Further supports come from recent studies of heat-shock proteins, such as Hsp90 and GroEl, which are thought to have evolved to protect organisms from environmental and developmental perturbations, but appear to also buffer against genetic perturbation in *Drosophila *[17], *Arabidopsis *[18], and *Escherichia coli *[19]. However, to date, the extent to which each of these evolutionary forces contributes to the evolution of robustness remains unresolved, partly due to the difficulty in providing evidence for robustness in natural biological systems [20].

Addressing this challenge, recent studies have resorted to bioinformatics and experimental approaches. One important effort to provide the evidence of environmental robustness has focused on the thermodynamic stability of noncoding RNA secondary structures [21-26]. Although the shuffling sequences generated in these studies rule out the bias of base composition, they do not preserve the structural phenotype of the native RNA sequences. Consequently, it cannot be determined whether the observed increased robustness goes beyond the intrinsic robustness of specific, functionally important structures. Comparing with environmental robustness, genetic robustness, on the other hand, is connected with major technical difficulties [20]. The classical approach has inferred genetic robustness from the increase in genetic variance after a major mutation or exposure to an environmental challenge during development [2], as exemplified by the measurements of vibrissae number in mice, ocelli in *D. subobscura*, and wing- and cross-vein interruptions and scutellar bristle numbers in *D. melanogaster*, which are all discussed in detail by Scharloo [27]. However, the evidence is often indirect and suffers from the lack of a natural reference genotype [20]. Experimental evolution is a more direct approach that has been applied to the study of robustness recently, which utilizes direct laboratory observation of short-term evolutionary processes, mostly in microbes [19,28]. Although its evolutionary potential is limited by time constraints, this approach does not suffer from a lack of control and promises exciting new data and insights for a more comprehensive theory of the evolution of genetic robustness [2].

Using a plausible background model is another strategy applied to elucidate the evolution of robustness, allowing evaluation of the significance of any greater robustness found in the wild-type (WT) [2]. Wagner and Stadler have compared the mutational stability of conserved and non-conserved elements in the secondary structure of a RNA viral genome with respect to point mutations, using non-conserved elements as the reference set [29]. Their research has demonstrated that the conserved elements show a consistently lower variability than non-conserved elements, suggesting that the virus evolves to a state of increased mutational robustness. While their data do not prove that selection that acts directly on the mutational stability of the RNA secondary structure, increases thermodynamic stability, this study do provide the first hint that "genetic canalization" (genetic robustness) can, in fact, evolve as a correlated response to selection for "environmental canalization" (thermodynamic stability), as predicted by population genetic models [1]. On the other hand, experimental studies have demonstrated that miRNA secondary structures are tolerant to some structural changes [30-33], with reports that miRNA precursors exhibit a significantly higher level of thermodynamic stability [21]. We therefore hypothesize that miRNA genetic robustness may evolve as a correlated side effect of the evolution for environmental robustness. To our knowledge, no systematic effort has been made to test this hypothesis in a genome-wide scale, with the exception of an experimental study on the effect of single point mutation for limited miRNAs [30-33].

Previous researches have demonstrated that miRNAs are abundant endogenous ~22-nucleotide (nt) noncoding RNAs, occupying between 1–5% of the genes in any given animal genome [34]. miRNAs regulate gene expression at the post-transcriptional level for cleavage or translational repression through the binding of a minimal-recognition 'seed' sequence [35-38]. Recent comparative phylogenetic studies have revealed that conserved miRNA-binding sequences are in more than one-third of all genes, suggesting that miRNA regulation may be relevant to a large portion of cellular processes [39-44]. Mature miRNAs are cleaved from ~70 nt precursors (pre-miRNA) that fold into a stem-loop hairpin structure, through the action of Dicer endonuclease [45-47]. The miRNA stem-loop structure is conserved in evolution, and plays a crucial role throughout miRNA gene maturation processing steps [33,46,48-50]. Additionally, there is a selective pressure to stabilize the stem-loop structure and some structural changes are tolerated [51], which may result in the evolution of robustness. As well, the use of program packages for RNA secondary structure prediction, such as Mfold [52] and Vienna RNA package [53], facilitates genotype-phenotype mapping and measurement of the structural robustness of a given miRNA stem-loop sequence, by comparing the predicted structure of this sequence with the predicted structure of all its one-mutant neighbors. Different types of reference sequences, with similar phenotypes and/or with exact or nearly exact mononucleotide and dinucleotide base composition as the real pre-miRNA, can be easily generated for miRNAs, allowing for careful control of the effects of secondary structure evolution. These merits make the miRNA stem-loop structure an ideal system to study the evolution of genetic robustness.

In our previous study, we have developed a method to quantitatively measure the genetic robustness of RNA secondary structure [54]. Here, we will apply this method to investigate the robustness of 1,082 miRNA genes from six different species. Our data suggest that the hairpin structures of miRNA precursors exhibit a significantly higher level of mutational robustness. Additionally, through the careful design of reference backgrounds, we show that this excess robustness goes beyond the intrinsic robustness of the stem-loop structure, and is not the byproduct of a base composition bias. Examination of the environmental robustness of real miRNA stem-loops also demonstrates that the phenotype of miRNAs buffers against genetic variations, at the same time is insensitive to environmental perturbations. These data suggest that the increase in genetic robustness may evolve as a correlated side effect of the evolution for environmental robustness.

## Results

**Figure 1:**
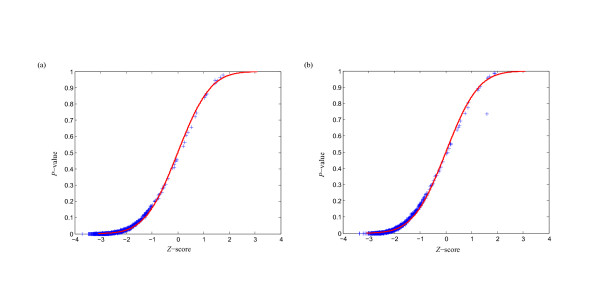
**Correlation between *Z*-score and *P-value *of genetic robustness for all the 1,082 miRNAs at threshold level *T*_1_**. For each real pre-miRNA, the robustness γ1m
 MathType@MTEF@5@5@+=feaafiart1ev1aaatCvAUfKttLearuWrP9MDH5MBPbIqV92AaeXatLxBI9gBaebbnrfifHhDYfgasaacPC6xNi=xH8viVGI8Gi=hEeeu0xXdbba9frFj0xb9qqpG0dXdb9aspeI8k8fiI+fsY=rqGqVepae9pg0db9vqaiVgFr0xfr=xfr=xc9adbaqaaeGacaGaaiaabeqaaeqabiWaaaGcbaacciGae83SdC2aa0baaSqaaiabigdaXaqaaiabd2gaTbaaaaa@3001@ is compared with that of 1,000 random sequences (a) and random pseudo pre-miRNAs (b), and the *Z*-score and *P*-value are computed. The curve for a normal distribution of mean 0 and a standard deviation of 1 is also displayed.

**Figure 2:**
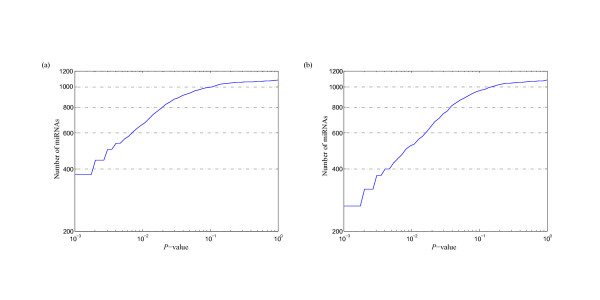
***P*-value distribution of genetic robustness for the 1,082 miRNAs at threshold level *T*_1_**. For each real pre-miRNA, the robustness γ1m
 MathType@MTEF@5@5@+=feaafiart1ev1aaatCvAUfKttLearuWrP9MDH5MBPbIqV92AaeXatLxBI9gBaebbnrfifHhDYfgasaacPC6xNi=xH8viVGI8Gi=hEeeu0xXdbba9frFj0xb9qqpG0dXdb9aspeI8k8fiI+fsY=rqGqVepae9pg0db9vqaiVgFr0xfr=xfr=xc9adbaqaaeGacaGaaiaabeqaaeqabiWaaaGcbaacciGae83SdC2aa0baaSqaaiabigdaXaqaaiabd2gaTbaaaaa@3001@ is compared with that of 1,000 random sequences (a) and random pseudo pre-miRNAs (b).

**Figure 3:**
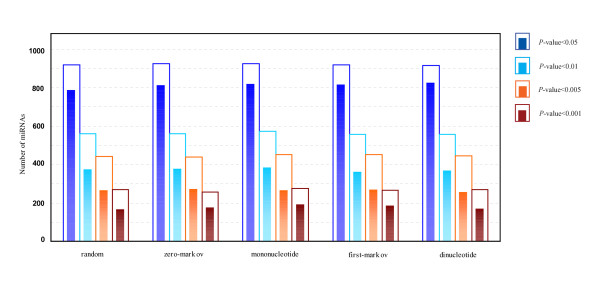
**Number of genetically robust miRNAs with FDR-controlled *P*-values of < 0.05, 0.01, 0.005, and 0.001 at threshold level *T*_1_**. **2D histogram plots of the number of miRNAs with significantly genetic robustness**. Each bar is constituted with an outer hollow sub-bar and an inner solid sub-bar, which represents the number of significantly robust real pre-miRNAs compared with random/shuffled sequences and random/shuffled pseudo pre-miRNAs, respectively.

For each real pre-miRNA, the robustness γ1m
 MathType@MTEF@5@5@+=feaafiart1ev1aaatCvAUfKttLearuWrP9MDH5MBPbIqV92AaeXatLxBI9gBaebbnrfifHhDYfgasaacPC6xNi=xH8viVGI8Gi=hEeeu0xXdbba9frFj0xb9qqpG0dXdb9aspeI8k8fiI+fsY=rqGqVepae9pg0db9vqaiVgFr0xfr=xfr=xc9adbaqaaeGacaGaaiaabeqaaeqabiWaaaGcbaacciGae83SdC2aa0baaSqaaiabigdaXaqaaiabd2gaTbaaaaa@3001@ at threshold level *T*_1 _is compared with that of 1,000 random sequences, and the *P*-value versus *Z*-score is showed in Figure 1(a). Figure 2(a) shows the corresponding *P*-value distribution of genetic robustness for the 1,082 miRNAs at threshold level *T*_1_. Of the 1,082 miRNA investigated, 920 (85.0%), 534 (49.4%), 376 (34.8%) miRNA genes show significant increases in robustness at FDR-controlled *P*-values of < 0.05, < 0.01, and < 0.005, respectively (Figure 3 and Table S1, Additional file 15). We further compare the robustness *y*_1 _of each real pre-miRNA with that of 1,000 pseudo pre-miRNAs, and the *P*-value versus *Z*-score is showed in Figure 1(b), so as to exclude the intrinsic robustness associated with the stem-loop hairpin structure. The corresponding *P*-value distribution of genetic robustness for the 1,082 miRNAs at threshold level *T*_1 _is showed in Figure 2(a). Our results suggest 787 (72.7%) miRNA genes of 1,082 are significantly more robust than pseudo pre-miRNAs at FDR-controlled *P*-value of < 0.05 (Figure 3 and Table S2, Additional file 15). These data suggest that the genetics of miRNA robustness is not a byproduct of the specific stem-loop structure. Additionally, investigation of the robustness *y*_1 _of miRNA genes within species is similar for each species individually (Table 1 – 2).

**Table 1:** *Z*-scores of the robustness γ1m
 MathType@MTEF@5@5@+=feaafiart1ev1aaatCvAUfKttLearuWrP9MDH5MBPbIqV92AaeXatLxBI9gBaebbnrfifHhDYfgasaacPC6xNi=xH8viVGI8Gi=hEeeu0xXdbba9frFj0xb9qqpG0dXdb9aspeI8k8fiI+fsY=rqGqVepae9pg0db9vqaiVgFr0xfr=xfr=xc9adbaqaaeGacaGaaiaabeqaaeqabiWaaaGcbaacciGae83SdC2aa0baaSqaaiabigdaXaqaaiabd2gaTbaaaaa@3001@ at threshold level *T*_1_. Rates for each real pre-miRNA compared to 1,000 random and four types of shuffled sequences.

Species	Random	Zero-markov	Mononucleotide	First-markov	Dinucleotide
*H. sapiens*	-2.39 ± 0.64	-2.42 ± 0.63	-2.42 ± 0.63	-2.45 ± 0.63	-2.44 ± 0.64
*C. elegans*	-2.50 ± 0.92	-2.48 ± 0.89	-2.47 ± 0.89	-2.52 ± 0.90	-2.49 ± 0.89
*D. melanogaster*	-2.44 ± 0.75	-2.39 ± 0.74	-2.39 ± 0.74	-2.43 ± 0.74	-2.40 ± 0.73
*D. rerio*	-2.39 ± 0.59	-2.40 ± 0.58	-2.40 ± 0.57	-2.44 ± 0.57	-2.41 ± 0.57
*M. musculus*	-2.23 ± 0.79	-2.25 ± 0.77	-2.26 ± 0.77	-2.29 ± 0.77	-2.27 ± 0.76
*R. norvegicus*	-2.32 ± 0.78	-2.37 ± 0.75	-2.37 ± 0.76	-2.40 ± 0.76	-2.37 ± 0.75
*Average*	-2.37 ± 0.71	-2.38 ± 0.69	-2.38 ± 0.69	-2.42 ± 0.70	-2.40 ± 0.69

**Table 2:** *Z*-scores of the robustness γ1m
 MathType@MTEF@5@5@+=feaafiart1ev1aaatCvAUfKttLearuWrP9MDH5MBPbIqV92AaeXatLxBI9gBaebbnrfifHhDYfgasaacPC6xNi=xH8viVGI8Gi=hEeeu0xXdbba9frFj0xb9qqpG0dXdb9aspeI8k8fiI+fsY=rqGqVepae9pg0db9vqaiVgFr0xfr=xfr=xc9adbaqaaeGacaGaaiaabeqaaeqabiWaaaGcbaacciGae83SdC2aa0baaSqaaiabigdaXaqaaiabd2gaTbaaaaa@3001@ at threshold level *T*_1_. Rates for each real pre-miRNA compared to 1,000 random and four types of shuffled pseudo pre-miRNAs.

Species	Random	Zero-markov	Mononucleotide	First-markov	Dinucleotide
*H. sapiens*	-2.11 ± 0.62	-2.14 ± 0.61	-2.14 ± 0.61	-2.17 ± 0.61	-2.17 ± 0.62
*C. elegans*	-2.18 ± 0.89	-2.17 ± 0.86	-2.16 ± 0.86	-2.20 ± 0.87	-2.18 ± 0.84
*D. melanogaster*	-2.14 ± 0.74	-2.11 ± 0.71	-2.07 ± 0.80	-2.15 ± 0.72	-2.15 ± 0.72
*D. rerio*	-2.07 ± 0.61	-2.11 ± 0.56	-2.13 ± 0.67	-2.13 ± 0.56	-2.11 ± 0.55
*M. musculus*	-1.96 ± 0.77	-2.00 ± 0.74	-2.00 ± 0.75	-2.04 ± 0.75	-2.02 ± 0.73
*R. norvegicus*	-2.02 ± 0.75	-2.07 ± 0.73	-2.08 ± 0.73	-2.09 ± 0.73	-2.07 ± 0.72
*Average*	-2.07 ± 0.70	-2.10 ± 0.67	-2.10 ± 0.68	-2.13 ± 0.67	-2.11 ± 0.66

The mononucleotide and dinucleotide frequencies of an RNA sequence, not preserved in random sequences, are crucial for the physical stability of the secondary structure [21-23,26]. It is consequently essential to verify that the greater robustness of real pre-miRNAs is not a byproduct of a bias in the base composition of the real pre-miRNA sequences, compared with random sequences. To this end, we generate four types of shuffled miRNAs that preserve the exact or nearly exact mononucleotide and dinucleotide base composition as the real pre-miRNA (see Methods). The robustness γ1m
 MathType@MTEF@5@5@+=feaafiart1ev1aaatCvAUfKttLearuWrP9MDH5MBPbIqV92AaeXatLxBI9gBaebbnrfifHhDYfgasaacPC6xNi=xH8viVGI8Gi=hEeeu0xXdbba9frFj0xb9qqpG0dXdb9aspeI8k8fiI+fsY=rqGqVepae9pg0db9vqaiVgFr0xfr=xfr=xc9adbaqaaeGacaGaaiaabeqaaeqabiWaaaGcbaacciGae83SdC2aa0baaSqaaiabigdaXaqaaiabd2gaTbaaaaa@3001@ of each real pre-miRNA is compared with that of 1,000 shuffled sequences generated by four types of shuffled methods, and the *P*-value versus *Z*-score is showed (Additional files 1 and 2). More than 910 of the 1,082 (>84% for the four types of shuffled methods) miRNA sequences have a significantly larger γ1m
 MathType@MTEF@5@5@+=feaafiart1ev1aaatCvAUfKttLearuWrP9MDH5MBPbIqV92AaeXatLxBI9gBaebbnrfifHhDYfgasaacPC6xNi=xH8viVGI8Gi=hEeeu0xXdbba9frFj0xb9qqpG0dXdb9aspeI8k8fiI+fsY=rqGqVepae9pg0db9vqaiVgFr0xfr=xfr=xc9adbaqaaeGacaGaaiaabeqaaeqabiWaaaGcbaacciGae83SdC2aa0baaSqaaiabigdaXaqaaiabd2gaTbaaaaa@3001@ at FDR-controlled *P*-value of < 0.05, with a high proportion (>34% for the four types of shuffled methods) is also observed at FDR-controlled *P*-value of < 0.005 (Figure 3 and Table S1, Additional file 15). To further confirm that the excess robustness goes beyond the intrinsic robustness of the stem-loop structure, we generate four types of reference sets for each miRNA, consisting of 1,000 shuffled pseudo pre-miRNAs that preserve not only the stem-loop structure, but also the exact or nearly exact mononucleotide and dinucleotide frequencies as real pre-miRNA (see Methods). Of the 1,082 miRNA genes, over 810 (>75%) miRNAs show significant more robust than shuffled pseudo pre-miRNA sequences at FDR-controlled *P*-value of < 0.05 (Figure 3, Table 2, Additional files 3 and 4, and Table S2- Additional file 15), suggesting that the genetic robustness of miRNA is not a byproduct of the specific stem-loop structure and that there is no bias of the base composition on the intrinsic robustness analysis. These results also demonstrate that the different types of shuffled methods are indistinguishable (Figures 3, Additional files 1, 2, 3, 4, Tables 1, 2, and Table S1–S2, Additional file 15).

**Figure 4:**
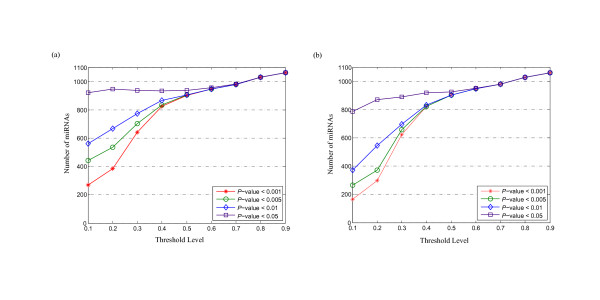
**Number of genetically robust miRNAs with FDR-controlled *P*-values of < 0.05, 0.01, 0.005, and 0.001 at different threshold levels**. For each real pre-miRNA, the robustness γim
 MathType@MTEF@5@5@+=feaafiart1ev1aaatCvAUfKttLearuWrP9MDH5MBPbIqV92AaeXatLxBI9gBaebbnrfifHhDYfgasaacPC6xNi=xH8viVGI8Gi=hEeeu0xXdbba9frFj0xb9qqpG0dXdb9aspeI8k8fiI+fsY=rqGqVepae9pg0db9vqaiVgFr0xfr=xfr=xc9adbaqaaeGacaGaaiaabeqaaeqabiWaaaGcbaacciGae83SdC2aa0baaSqaaiabdMgaPbqaaiabd2gaTbaaaaa@306C@, *i *= 1, 2, ⋯, 9 is compared with 1,000 random sequences (a) and random pseudo pre-miRNAs (b).

To test the effect of the threshold level on robustness, we also analyze the genetic robustness at different threshold levels (from *T*_1 _to *T*_9_). Figure 4 shows the number of significantly robust miRNAs with FDR-controlled *P*-values of < 0.05, < 0.01, < 0.005, and < 0.001 at different threshold levels, where the robustness γim
 MathType@MTEF@5@5@+=feaafiart1ev1aaatCvAUfKttLearuWrP9MDH5MBPbIqV92AaeXatLxBI9gBaebbnrfifHhDYfgasaacPC6xNi=xH8viVGI8Gi=hEeeu0xXdbba9frFj0xb9qqpG0dXdb9aspeI8k8fiI+fsY=rqGqVepae9pg0db9vqaiVgFr0xfr=xfr=xc9adbaqaaeGacaGaaiaabeqaaeqabiWaaaGcbaacciGae83SdC2aa0baaSqaaiabdMgaPbqaaiabd2gaTbaaaaa@306C@, *i *= 1, 2, ⋯, 9 for each real pre-miRNA is compared to that of 1,000 random sequences and pseudo pre-miRNA sequences at each threshold level. Increased threshold levels result in a rapid increase in the number of robust miRNA sequences. After threshold level *T*_6_, the number of miRNAs with FDR-controlled *P*-values of < 0.05, < 0.01, < 0.005, and < 0.001 as filters is almost identical, and is almost equal to 1,082 at *T*_9_. Similarly, we also show the number of significantly robust miRNAs with different FDR-controlled *P*-values at different threshold levels, where the robustness γim
 MathType@MTEF@5@5@+=feaafiart1ev1aaatCvAUfKttLearuWrP9MDH5MBPbIqV92AaeXatLxBI9gBaebbnrfifHhDYfgasaacPC6xNi=xH8viVGI8Gi=hEeeu0xXdbba9frFj0xb9qqpG0dXdb9aspeI8k8fiI+fsY=rqGqVepae9pg0db9vqaiVgFr0xfr=xfr=xc9adbaqaaeGacaGaaiaabeqaaeqabiWaaaGcbaacciGae83SdC2aa0baaSqaaiabdMgaPbqaaiabd2gaTbaaaaa@306C@, *i *= 1, 2, ⋯, 9 of each real pre-miRNA, is compared with that of 1,000 shuffled sequences and shuffled pseudo pre-miRNAs at each threshold level, respectively (Additional files 5 and 6). These data demonstrate the threshold levels have not affect robustness and the different types of shuffled methods are indistinguishable (Figure 4 and Additional files 5 and 6).

**Figure 5:**
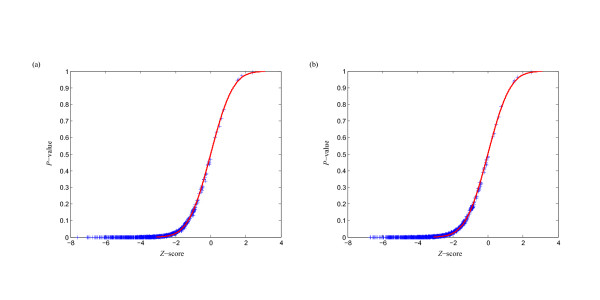
**Correlation between *Z*-scores and *P-values *of environmental robustness for all the 1,082 miRNAs**. For each real pre-miRNA, the free energy is compared with that of 1,000 random sequences (a) and random pseudo pre-miRNAs (b), and the *Z*-score and *P*-value are computed. The curve for a normal distribution of mean 0 and a standard deviation of 1 is also displayed.

**Figure 6:**
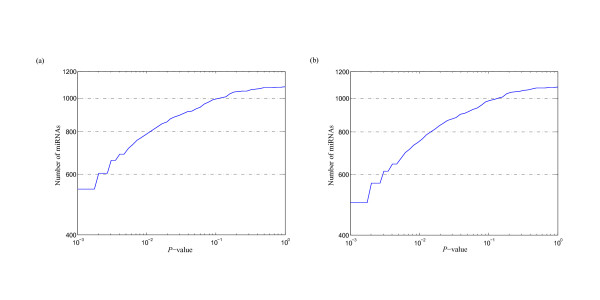
***P*-value distribution of environmental robustness for the 1,082 miRNAs**. For each real pre-miRNA, the free energy is compared with that of 1,000 random sequences (a) and random pseudo pre-miRNAs (b).

**Figure 7:**
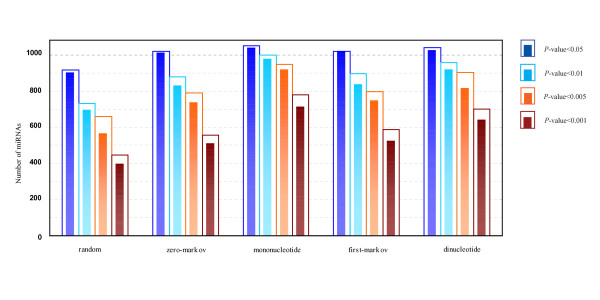
**Number of miRNAs with significantly environmental robustness at FDR-controlled *P*-values of < 0.05, 0.01, 0.005, and 0.001**. **2D histogram plots of the number of miRNAs with significantly genetic and environmental robustness**. Each bar is constituted with an outer hollow sub-bar and an inner solid sub-bar, which represents the number of significantly robust real pre-miRNAs compared with random/shuffled sequences and random/shuffled pseudo pre-miRNAs, respectively.

Due to the correlation between the thermodynamic stability of the minimum free energy structure of a given sequence and its genetics robustness [55], the increased genetic robustness described above may arise from the increased thermodynamic stability of miRNAs, as recently reported by Bonnet *et al*. [21]. If so, genetic robustness may have evolved as a correlated side effect of environmental robustness. Here, we not only examine the thermodynamic stability of miRNAs in an analogous manner to that done by Bonnet *et al*. [21] but also using a background model, based on random and shuffled pseudo pre-miRNAs, that maintain the stem-loop structure as in the real pre-miRNA (see Methods). Figure 5 graphs *P*-value against *Z*-score of environmental robustness for all the 1,082 miRNAs, and the corresponding *P*-value distribution is showed in Figure 6. Comparison with random sequences suggests 917 out of 1,082 (84.8%) show a high level of thermodynamic stability at FDR-controlled *P*-value of < 0.05, similar to that previously reported [21] (Figure 7, Table 3, and Table S3, Additional file 15). As comparing with random pseudo pre-miRNAs, significant thermodynamic stability is also observed in most miRNAs (904 of 1,082, 83.5%) at FDR-controlled *P*-value of < 0.05, suggesting that it is not a byproduct of the special stem-loop structure (Figure 7, Table 4, and Table S4, Additional file 15). There are also a high proportion of miRNA sequences at each FDR-controlled *P*-value (Figure 7, Table 3, Additional files 7 and 8, and Table S3, Additional file 15), when compared with the four types of shuffling sequences. Over 90% of the 1,082 miRNA sequences show a high level of thermodynamic stability at FDR-controlled *P*-value of < 0.05, based on four types of shuffled methods (Figure 7, Table 3 and Table S3, Additional file 15). There is also a high proportion of miRNAs (>72% for the four types of shuffled methods) with FDR-controlled *P*-value < 0.005 as filter (Figure 7, Table 3, and Table S3, Additional file 15). The reference sets of shuffled pseudo pre-miRNAs generated for each miRNA are utilized to further confirm that the increased thermodynamic stability is not the product of the specific stem-loop structure (Figure 7, Additional files 9 and 10, Table 4, and Table S4- Additional file 15). Our data suggest that more than 1010 of the 1,082 (>93% for the four types of shuffled methods) miRNA sequences show a high level of thermodynamic stability at FDR-controlled *P*-value of < 0.05, with a high proportion (>68% for the four types of shuffled methods) is also observed at FDR-controlled *P*-value of < 0.005 (Figure 7, Table 4, and Table S4- Additional file 15). Additionally, examining the thermodynamic stability of miRNA genes within species provides a similar picture for each species separately (Figure 7, Additional files 7, 8, 9, 10, Table 3 – 4, and Additional file 15, Tables S3–S4).

**Table 3:** *Z*-scores of free energy. Comparison between each real pre-miRNA and 1,000 random and four types of shuffled sequences.

Species	Random	Zero-markov	Mononucleotide	First-markov	Dinucleotide
*H. sapiens*	-3.96 ± 1.27	-4.31 ± 1.40	-4.36 ± 1.40	-6.05 ± 1.85	-5.63 ± 1.87
*C. elegans*	-2.51 ± 1.52	-3.34 ± 1.45	-3.37 ± 1.46	-4.85 ± 2.18	-4.87 ± 2.09
*D. melanogaster*	-2.30 ± 1.05	-3.53 ± 1.08	-3.53 ± 1.08	-4.83 ± 1.51	-4.85 ± 1.56
*D. rerio*	-2.87 ± 1.33	-3.53 ± 1.19	-3.64 ± 1.23	-4.91 ± 1.69	-4.67 ± 1.64
*M. musculus*	-3.63 ± 1.25	-3.89 ± 1.40	-3.87 ± 1.34	-5.41 ± 1.83	-4.95 ± 1.84
*R. norvegicus*	-3.68 ± 1.33	-3.60 ± 1.30	-3.68 ± 1.33	-5.25 ± 1.80	-4.80 ± 1.86
*Average*	-3.26 ± 1.42	-3.75 ± 1.35	-3.81 ± 1.35	-5.28 ± 1.86	-4.98 ± 1.83

**Table 4:** *Z*-scores of free energy. Comparison between each real pre-miRNA and 1,000 random and four types of shuffled pseudo pre-miRNAs

Species	Random	Zero-markov	Mononucleotide	First-markov	Dinucleotide
*H. sapiens*	-3.81 ± 1.22	-4.16 ± 1.38	-4.22 ± 1.36	-5.73 ± 1.78	-5.27 ± 1.79
*C. elegans*	-2.39 ± 1.49	-3.22 ± 1.42	-3.25 ± 1.42	-4.57 ± 2.05	-4.59 ± 2.01
*D. melanogaster*	-2.20 ± 1.03	-3.42 ± 1.06	-3.43 ± 1.04	-4.57 ± 1.46	-4.61 ± 1.52
*D. rerio*	-2.74 ± 1.29	-3.40 ± 1.16	-3.51 ± 1.19	-4.61 ± 1.62	-4.37 ± 1.58
*M. musculus*	-3.48 ± 1.20	-3.75 ± 1.37	-3.74 ± 1.33	-5.12 ± 1.76	-4.62 ± 1.78
*R. norvegicus*	-3.52 ± 1.25	-3.44 ± 1.26	-3.51 ± 1.29	-4.96 ± 1.74	-4.46 ± 1.77
*Average*	-3.12 ± 1.37	-3.62 ± 1.32	-3.68 ± 1.32	-4.98 ± 1.78	-4.66 ± 1.76

**Figure 8:**
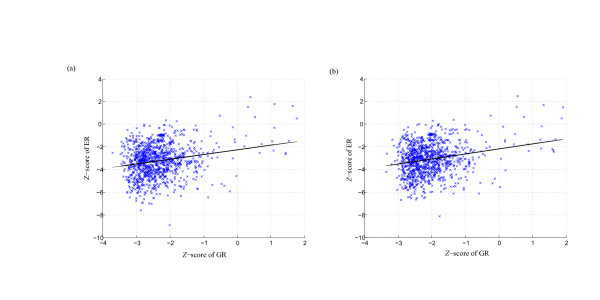
**Correlation between genetic and environmental robustness**. Scatter plots of *Z*-scores of genetic robustness and environmental robustness. The *Z*-scores are obtained by comparing the robustness of real pre-miRNAs with that of 1,000 random sequences (a) and random pseudo pre-miRNAs (b).

**Figure 9:**
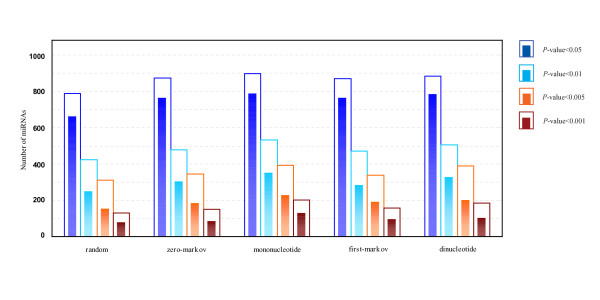
**Number of miRNAs with significantly genetic and environmental robustness at FDR-controlled *P*-values of < 0.05, 0.01, 0.005, and 0.001**. **2D histogram plots of the number of miRNAs with significantly genetic and environmental robustness**. Each bar is constituted with an outer hollow sub-bar and an inner solid sub-bar, which represents the number of significantly robust real pre-miRNAs compared with random/shuffled sequences and random/shuffled pseudo pre-miRNAs, respectively.

**Figure 10:**
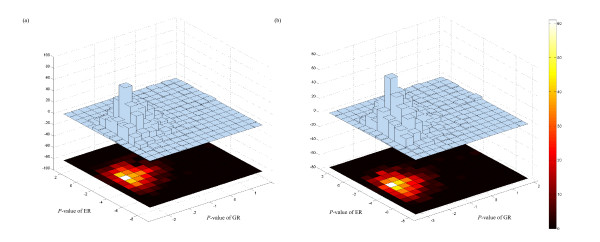
**Distribution of *Z*-score of genetic and environmental robustness**. 3D histogram plots of *Z*-scores of genetic and environmental robustness. The *Z*-scores are obtained by comparing the robustness of real pre-miRNAs with that of 1,000 random sequences (a) and random pseudo pre-miRNAs (b).

We also investigate the correlation between genetic robustness and thermodynamic stability of miRNA. Figure 8 shows the scatter plots of *Z*-scores of genetic and environmental robustness for all the 1,082 miRNAs. Although a strong correlation is not observed (Pearson's correlation coefficient is 0.43, 0.40 for random sequences and random pseudo pre-miRNAs, respectively), most of the significantly genetic robust miRNA genes exhibit significant thermodynamic stability at the same time. Of the 1,082 miRNA genes investigated, 789 (72.9%) and 662 (61.2%) miRNAs are showed significantly genetic and environmental robustness with FDR-controlled *P*-value of < 0.05 (Figure 9, Table S5 and S6 Additional file 15), compared with random sequences and random pseudo pre-miRNAs, respectively. These also can be seen from the 3D histogram plots of *Z*-scores of genetic and environmental robustness (Figure 10). Furthermore, the correlation between the genetic and environmental robustness is not a byproduct of a base composition bias (Additional files 11, 12, 13, 14 and Tables S5–S6 Additional file 15). Our results demonstrate that the stem-loop structures of miRNAs buffer against genetic variations, at the same time is also insensitive to environmental perturbations, indicating that the increase in genetic robustness may evolve as a correlated side effect of the evolution for environmental robustness.

## Discussion

There has been a growing interest of evolutionary biologists in the origins, mechanisms and consequences of robustness [2], which is a fundamental and ubiquitously observed property of biological systems [8]. A greater understanding of the evolution of robustness is needed to clarify whether genetic robustness evolved as a direct consequence of natural selection (adaptive robustness), as a byproduct of stabilizing selection acting on fitness-related traits (intrinsic robustness), or as a correlated response to environmental robustness (congruent robustness) [2]. Recent studies have demonstrated that the stem-loop structures of miRNA are tolerant to some structural changes and show thermodynamic stability. We therefore hypothesize that the genetic robustness of miRNAs may evolve as a correlated side effect of the evolution for environmental robustness in the current study. Using the method proposed in our previous study [54], we examine the robustness of 1,082 miRNA genes covering six species, and show that the stem-loop structures of miRNA precursors exhibit a significantly higher level of genetic robustness at different FDR-controlled *P*-values. Additionally, we generate a reference background of phenotypes similar to real pre-miRNAs (pseudo pre-miRNAs), and demonstrate that this excess robustness of miRNA genes goes beyond the intrinsic robustness of the stem-loop structure. The effect of base composition bias on robustness is ruled out by careful design of corresponding shuffling reference backgrounds. Examination of the environmental robustness of real pre-miRNAs also demonstrates that the phenotype of miRNA buffers against genetic perturbations, and at the same time is insensitive to environmental perturbations. These results suggest that increased genetic robustness may evolve as a correlated side effect of the evolution for environmental robustness.

Pang et al. [56] argue that even slight changes in miRNA sequences can fundamentally alter their function, when they interpret the conservation of miRNAs and snoRNAs. Our data suggest this interpretation might be premature. Experimental studies also have demonstrated that stem-loop structures of miRNAs are, to a certain degree, tolerant to some structural changes, as exemplified by studies in which no effects are noted resulting from changes in the loop sequence [30-33] and further suggesting that the sequence/structure requirements for miRNA processing are quite lax, in agreement with our results.

A recent study, published during the course of our work, reports that the stem-loop structures of miRNA precursors show excess robustness with respect to mutational perturbation, compared with random RNA sequences with similar stem-loop structures [57], suggesting that this excess robustness of miRNAs goes beyond the intrinsic robustness of the stem-loop hairpin structure. The study further demonstrates that this excess robustness is not the byproduct of a base composition bias. These results are in much agreement with our findings, although the quantitative measures of genetic robustness defined in these two studies are quite different. The authors then reexamine the thermodynamic stability of miRNA utilizing the method used by Bonnet *et al*. [21], with a background model based on inversely folded sequences rather than the shuffled sequences, and find that most of the statistical effect vanished, suggesting that the excess robustness of miRNA stem-loops is the result of direct evolutionary pressure toward increased robustness. The different conclusions (adaptive robustness vs. congruent robustness) may have resulted from differences in the reference backgrounds employed. The reference backgrounds in our study are made up of random and shuffled pseudo pre-miRNA sequences with preserved phenotypes that are similar to real pre-miRNAs (see Methods). Their reference backgrounds, on the other hand, are produced by inverse folding.

On the other hand, Hermission and Wagner classify robustness as adaptive and intrinsic [58]. The adaptive robustness in their definition encompass both adaptive and congruent scenarios within the classification system established by de Visser *et al*. [2]. They consider robustness to be adaptive if the buffering of that trait with respect to some source of variation has the target of natural selection. In other words, robust character states are selected because of their reduced variability. According to this view, our study and the study of Borenstein and Ruppin [57] come to the same conclusion of adaptive robustness, which is a property that evolves for its own sake. The difference is that the natural forces, assumed to be responsible for its evolution, function as a kind of stabilizing selection acting directly on a character, or on some highly correlated pleiotropic trait [58].

Our study will facilitate the in silico identification of novel miRNAs. Computational identification of miRNAs is based largely on the phylogenetic conservation and the structural characteristics of miRNA precursors [59-61]. A recent study integrating bioinformatics predictions with microarray analysis and sequence-directed cloning, that does not include inter-species conservation, identifies hundreds of new human miRNAs, many of which are poorly conserved beyond primates [62]. The significantly greater robustness of the miRNA stem-loop structures that buffers environmental and genetic perturbations may also facilitate improved miRNA identification on a single genome and can serve as a complementary method to filter out random pseudo pre-miRNA sequences. Our findings may also be utilized for the improvement of in vitro selection or SELEX (Systematic Evolution of Ligands by Exponential enrichment), an experimental method for selecting functional RNAs from a large pool (10^15^) of random sequences [63,64]. The use of designed sequences with thermodynamic stability and genetic robustness, in lieu of random sequences, may increase the probability of identifying novel functional RNAs.

The in silico approach utilized in the current study is a simple yet powerful, biologically well-grounded model for studying the evolution of genetic robustness. The importance of this computational approach is emphasized by the fact that the large numbers of perturbations (more than 2 × 10^9 ^point mutants are generated and folded) analyzed here are not easily studied in laboratory experiments. Although this in silico study can not completely rule out all alternative hypotheses, the carefully designed reference background model and the statistical analysis of the results strongly support the hypothesis of congruent evolution of robustness. A greater understanding of the evolution of robustness will require quantitative knowledge of the forces producing robustness, such as the distribution of fitness effects of mutations [12]. Further researches are needed to fully elucidate the mechanisms of the evolution of robustness.

## Conclusion

The current study investigate the evolutionary origin of genetic robustness – a fundamental evolutionary molecular biology problem which has not been fully elucidated. We have shown that miRNA stem-loop structures exhibit a significantly higher level of genetic robustness at different FDR-controlled *P*-values, which goes beyond the intrinsic robustness of the stem-loop structure and is not a byproduct of a base composition bias. Furthermore, we have demonstrated that the phenotypes of miRNAs buffer against genetic perturbations, and at the same time are also insensitive to environmental perturbations. Our findings suggest that the increased robustness of miRNA stem-loops may evolve as a correlated side effect of evolution for environmental robustness.

## Methods

### Real pre-miRNAs, reference sets and RNA folding

1,082 real pre-miRNA sequences included in the analysis are selected from MicroRNA Registry release 7.1, which all have been experimentally verified to avoid a possible bias introduced by consideration of predicted miRNA precursors (Table 5) [65,66]. The available sequences covered six species: *H. sapiens*, *D. melanogaster*, *D. rerio*, *C. elegans*, *M. musculus*, *and R. norvegicus*.

**Table 5:** Robustness analysis of miRNA within each species

Species	*N*_*s*_	Length	%GC	No. of genetically robust miRNAs (%)	No. of environmentally robust miRNAs (%)
*H. sapiens*	242	85.48 ± 14.49	47.67 ± 7.88	177(73.1)	233(96.3)
*C. elegans*	112	98.29 ± 6.42	44.61 ± 6. 89	93(83.0)	74(66.1)
*D. melanogaster*	75	87.67 ± 12.74	41.60 ± 5.39	54(72.0)	48(64.0)
*D. rerio*	350	94.36 ± 18.49	45.11 ± 6.51	254(72.6)	254(72.6)
*M. musculus*	191	80.26 ± 12.48	48.19 ± 8.26	130(68.1)	177(92.7)
*R. norvegicus*	112	90.96 ± 9.14	50.34 ± 7.96	77(68.8)	105(93.8)

*N*_*s*_, total number of experimentally verified miRNA sequences; Length, distribution of miRNA sequence length; %GC, distribution of GC content of miRNA sequences; No. of genetically (environmentally) robust miRNAs (%), the number of significantly genetically (environmentally) robust miRNAs (%) at FDR-controlled *P*-value of < 0.05, comparing with random pseudo pre-miRNAs.

In addition to the real pre-miRNAs specified in Table 5, a reference set consisting of 1,000 random RNA sequences for each real pre-miRNA is generated, and an additional reference set consisting of 1,000 hairpin sequences with similar stem-loops as real pre-miRNAs (pseudo pre-miRNAs) using a similar idea of the *RNAinverse *program in Vienna RNA package [53] is also generated to investigate whether the increased robustness stemmed intrinsically from the miRNA stem-loop structures. To rule out the effect of base composition bias on robustness, four types of reference sets for each real pre-miRNA are made using four different sequence shuffling methods: mononucleotide shuffling, dinucleotide shuffling and shuffling based on a zero and first order Markov model. Each type of reference set is also consisted of 1,000 shuffled sequences that preserved the exact or nearly exact mononucleotide and dinucleotide frequencies as real pre-miRNA. Additionally, four types of shuffling reference sets which consisted of 1,000 shuffled pseudo pre-miRNAs that preserved not only the stem-loop structure but also the exact or nearly exact mononucleotide and dinucleotide frequencies as real pre-miRNA are generated for each miRNA. These sequence shuffling methods first generate the corresponding shuffling sequences preserving exact or nearly exact mononucleotide and dinucleotide frequencies with the methods previously used in the studies of the thermodynamic stability of RNA secondary structures [21-23,26], and then search for the shuffling sequences with similar stem-loop as real pre-miRNAs using a similar idea of the *RNAinverse *program [53].

The *RNAfold *in Vienna RNA package (version 1.6) [53] is utilized with default parameter values (*T *= 37°*C*) to predict the secondary structures, based on Zuker's minimum free energy algorithm [52]. The current study only utilized optimal folding results.

### Robustness evaluation

Experimental researches have demonstrated that the secondary structures of miRNAs are tolerant to some structural changes [30-33]. To reflect this flexibility in sequence/structure requirements, at a given threshold, *T*_*j*_, we define the robustness [54], *γ*_*j*_, as follows:

*γ*_*j *_= ⟨*N*_*j*_(*d*)⟩, *j *= 1, 2, ..., 9.

where *d *is the structure distance between the secondary structure of the WT sequence and the secondary structure of the mutant, and *N*_*j*_(*d*) is number of mutants with structure distance lesser than or equal to the threshold *T*_*j*_. *γ*_*j *_is the average of *N*_*j*_(*d*) over all 3 × *L *one-mutant neighbors. The maximum value of the structural distance between the secondary structure of the WT sequence and the secondary structure of the mutants is used as a baseline value to evaluate the threshold level [54]. The threshold *T*_*j*_, *j *= 1, 2, ..., 9 is set to 10%, 20%, ..., 90% of the maximum value of the metric, respectively. The larger value of the robustness *γ*_*j *_at threshold *T*_*j *_indicates a relatively higher level of robustness.

We compare the secondary structure between WT and its mutant using a variety of distance measures for secondary structures [53,67-69], including topological indices [70], tree-edit and string-eidt distance [53,71], and base-pair distance [55]. While the data in this paper are obtained by base-pair distance, the qualitative results are obtained independent of the distance measure used.

### Statistical significance analysis

At each threshold *T*_*j*_, we evaluate the robustness γjm
 MathType@MTEF@5@5@+=feaafiart1ev1aaatCvAUfKttLearuWrP9MDH5MBPbIqV92AaeXatLxBI9gBaebbnrfifHhDYfgasaacPC6xNi=xH8viVGI8Gi=hEeeu0xXdbba9frFj0xb9qqpG0dXdb9aspeI8k8fiI+fsY=rqGqVepae9pg0db9vqaiVgFr0xfr=xfr=xc9adbaqaaeGacaGaaiaabeqaaeqabiWaaaGcbaacciGae83SdC2aa0baaSqaaiabdQgaQbqaaiabd2gaTbaaaaa@306E@ of the real pre-miRNA and ϒ_*j *_= {γjri
 MathType@MTEF@5@5@+=feaafiart1ev1aaatCvAUfKttLearuWrP9MDH5MBPbIqV92AaeXatLxBI9gBaebbnrfifHhDYfgasaacPC6xNi=xH8viVGI8Gi=hEeeu0xXdbba9frFj0xb9qqpG0dXdb9aspeI8k8fiI+fsY=rqGqVepae9pg0db9vqaiVgFr0xfr=xfr=xc9adbaqaaeGacaGaaiaabeqaaeqabiWaaaGcbaacciGae83SdC2aa0baaSqaaiabdQgaQbqaaiabdkhaYnaaBaaameaacqWGPbqAaeqaaaaaaaa@3200@, *i *= 1, 2, ..., *N*} of the corresponding 1,000 random sequences in reference set *X *(*N *is the number of the sequences in the reference set, |*X*|), then compare γjm
 MathType@MTEF@5@5@+=feaafiart1ev1aaatCvAUfKttLearuWrP9MDH5MBPbIqV92AaeXatLxBI9gBaebbnrfifHhDYfgasaacPC6xNi=xH8viVGI8Gi=hEeeu0xXdbba9frFj0xb9qqpG0dXdb9aspeI8k8fiI+fsY=rqGqVepae9pg0db9vqaiVgFr0xfr=xfr=xc9adbaqaaeGacaGaaiaabeqaaeqabiWaaaGcbaacciGae83SdC2aa0baaSqaaiabdQgaQbqaaiabd2gaTbaaaaa@306E@ with ϒ_*j*_. *Z*-score and *P*-value are employed here to determine whether the phenotype of a real pre-miRNA sequence shows significantly robust from that of reference sequences, which have been widely applied in statistical significance analysis [21-24,26,72]. The *Z*-score is the number of standard deviations by which γjm
 MathType@MTEF@5@5@+=feaafiart1ev1aaatCvAUfKttLearuWrP9MDH5MBPbIqV92AaeXatLxBI9gBaebbnrfifHhDYfgasaacPC6xNi=xH8viVGI8Gi=hEeeu0xXdbba9frFj0xb9qqpG0dXdb9aspeI8k8fiI+fsY=rqGqVepae9pg0db9vqaiVgFr0xfr=xfr=xc9adbaqaaeGacaGaaiaabeqaaeqabiWaaaGcbaacciGae83SdC2aa0baaSqaaiabdQgaQbqaaiabd2gaTbaaaaa@306E@ of a real pre-miRNA sequence differs from the mean γjri
 MathType@MTEF@5@5@+=feaafiart1ev1aaatCvAUfKttLearuWrP9MDH5MBPbIqV92AaeXatLxBI9gBaebbnrfifHhDYfgasaacPC6xNi=xH8viVGI8Gi=hEeeu0xXdbba9frFj0xb9qqpG0dXdb9aspeI8k8fiI+fsY=rqGqVepae9pg0db9vqaiVgFr0xfr=xfr=xc9adbaqaaeGacaGaaiaabeqaaeqabiWaaaGcbaacciGae83SdC2aa0baaSqaaiabdQgaQbqaaiabdkhaYnaaBaaameaacqWGPbqAaeqaaaaaaaa@3200@, *i *= 1, 2, ..., *N *of the random reference sequences set *X *and is defined as:

Z(γjm)=<ϒj>−γjσ(ϒj),j=1,2,…,9
 MathType@MTEF@5@5@+=feaafiart1ev1aaatCvAUfKttLearuWrP9MDH5MBPbIqV92AaeXatLxBI9gBaebbnrfifHhDYfgasaacPC6xNi=xI8qiVKYPFjYdHaVhbbf9v8qqaqFr0xc9vqFj0dXdbba91qpepeI8k8fiI+fsY=rqGqVepae9pg0db9vqaiVgFr0xfr=xfr=xc9adbaqaaeGacaGaaiaabeqaaeqabiWaaaGcbaqbaeqabeGaaaqaaiabdQfaAjabcIcaOGGaciab=n7aNnaaDaaaleaacqWGQbGAaeaacqWGTbqBaaGccqGGPaqkcqGH9aqpjuaGdaWcaaqaaiabgYda8iabfk9aHoaaBaaabaGaemOAaOgabeaacqGH+aGpcqGHsislcqWFZoWzdaWgaaqaaiabdQgaQbqabaaabaGae83WdmNaeiikaGIaeuO0de6aaSbaaeaacqWGQbGAaeqaaiabcMcaPaaakiabcYcaSaqaaiabdQgaQjabg2da9iabigdaXiabcYcaSiabikdaYiabcYcaSiablAciljabcYcaSiabiMda5aaaaaa@4FDE@

where <·> and *σ *(·) denote the mean and the standard deviation of the ϒ_*j*_. The *P*-value of γjm
 MathType@MTEF@5@5@+=feaafiart1ev1aaatCvAUfKttLearuWrP9MDH5MBPbIqV92AaeXatLxBI9gBaebbnrfifHhDYfgasaacPC6xNi=xH8viVGI8Gi=hEeeu0xXdbba9frFj0xb9qqpG0dXdb9aspeI8k8fiI+fsY=rqGqVepae9pg0db9vqaiVgFr0xfr=xfr=xc9adbaqaaeGacaGaaiaabeqaaeqabiWaaaGcbaacciGae83SdC2aa0baaSqaaiabdQgaQbqaaiabd2gaTbaaaaa@306E@ of a real pre-miRNA is the fraction of sequences in *X *having robustness larger than the real pre-miRNA sequence, that is, the area under the distribution function to the right of the γjm
 MathType@MTEF@5@5@+=feaafiart1ev1aaatCvAUfKttLearuWrP9MDH5MBPbIqV92AaeXatLxBI9gBaebbnrfifHhDYfgasaacPC6xNi=xH8viVGI8Gi=hEeeu0xXdbba9frFj0xb9qqpG0dXdb9aspeI8k8fiI+fsY=rqGqVepae9pg0db9vqaiVgFr0xfr=xfr=xc9adbaqaaeGacaGaaiaabeqaaeqabiWaaaGcbaacciGae83SdC2aa0baaSqaaiabdQgaQbqaaiabd2gaTbaaaaa@306E@, and is defined as:

P(γjm)=MN+1,j=1,2,…,9
 MathType@MTEF@5@5@+=feaafiart1ev1aaatCvAUfKttLearuWrP9MDH5MBPbIqV92AaeXatLxBI9gBaebbnrfifHhDYfgasaacPC6xNi=xI8qiVKYPFjYdHaVhbbf9v8qqaqFr0xc9vqFj0dXdbba91qpepeI8k8fiI+fsY=rqGqVepae9pg0db9vqaiVgFr0xfr=xfr=xc9adbaqaaeGacaGaaiaabeqaaeqabiWaaaGcbaqbaeqabeGaaaqaaiabdcfaqjabcIcaOGGaciab=n7aNnaaDaaaleaacqWGQbGAaeaacqWGTbqBaaGccqGGPaqkcqGH9aqpjuaGdaWcaaqaaiabd2eanbqaaiabd6eaojabgUcaRiabigdaXaaakiabcYcaSaqaaiabdQgaQjabg2da9iabigdaXiabcYcaSiabikdaYiabcYcaSiablAciljabcYcaSiabiMda5aaaaaa@435D@

where *M *is the number of sequences in *X *with larger robustness than γjm
 MathType@MTEF@5@5@+=feaafiart1ev1aaatCvAUfKttLearuWrP9MDH5MBPbIqV92AaeXatLxBI9gBaebbnrfifHhDYfgasaacPC6xNi=xH8viVGI8Gi=hEeeu0xXdbba9frFj0xb9qqpG0dXdb9aspeI8k8fiI+fsY=rqGqVepae9pg0db9vqaiVgFr0xfr=xfr=xc9adbaqaaeGacaGaaiaabeqaaeqabiWaaaGcbaacciGae83SdC2aa0baaSqaaiabdQgaQbqaaiabd2gaTbaaaaa@306E@ of the real pre-miRNA sequence.

The statistical significance analysis of environmental robustness is similar to that done for genetic robustness, in which the robustness *γ*_*j *_at a threshold *T*_*j *_is simply replaced by the free energy of the sequences. The thermodynamic stability of pre-miRNAs is examined as previously reported [21], but also used a background model based on the random and shuffled pseudo pre-miRNAs.

### False discovery rate

Because the above statistical significance analysis involves the simultaneous testing of thousands of hypotheses, multiple hypotheses testing is important to control the overall Type I error rate. We will use direct control of the false discoveries using the commonly applied FDR criterion. The FDR, based on the outcomes of *m *statistical tests (Table 6), is defined as the expected proportion of false positives among the rejected hypotheses, i.e.

**Table 6:** Possible outcomes of the *m *statistical tests.

	Called not significant	Called significant	Total
Null true	*U*	*V*	*m*_0_
Alternative true	*T*	*S*	*m - m*_0_
Total	*M *- *R*	*R*	*m*

*N*_*s*_, total number of experimentally verified miRNA sequences; Length, distribution of miRNA sequence length; %GC, distribution of GC content of miRNA sequences; No. of genetically (environmentally) robust miRNAs (%), the number of significantly genetically (environmentally) robust miRNAs (%) at FDR-controlled *P*-value of < 0.05, comparing with random pseudo pre-miRNAs.

FDR=E(VR|R>0)·Pr⁡(R>0)
 MathType@MTEF@5@5@+=feaafiart1ev1aaatCvAUfKttLearuWrP9MDH5MBPbIqV92AaeXatLxBI9gBaebbnrfifHhDYfgasaacPC6xNi=xI8qiVKYPFjYdHaVhbbf9v8qqaqFr0xc9vqFj0dXdbba91qpepeI8k8fiI+fsY=rqGqVepae9pg0db9vqaiVgFr0xfr=xfr=xc9adbaqaaeGacaGaaiaabeqaaeqabiWaaaGcbaGaemOrayKaemiraqKaemOuaiLaeyypa0JaemyrauKaeiikaGscfa4aaSaaaeaacqWGwbGvaeaacqWGsbGuaaGcdaabbaqaaiabdkfasjabg6da+iabicdaWaGaay5bSdGaeiykaKIaeS4JPFMagiiuaaLaeiOCaiNaeiikaGIaemOuaiLaeyOpa4JaeGimaaJaeiykaKcaaa@44DB@

where *V *is the number of false positives and *R *is the number of rejected hypotheses. Therefore, an FDR cut-off has a meaningful interpretation.

To compute the FDR, we apply the Benjamini Hochberg procedure [73]. Considering testing *H*_1_, *H*_2_, ⋯, *H*_*m *_based on the corresponding *P*-values *P*_(1)_, *P*_(2)_, ⋯, *P*_(*m*)_. Let *P*_(1) _≤ *P*_(2) _≤ ⋯ ≤ *P*_(*m*) _be the ordered *P*-values, and denote by *H*_(*i*) _the null hypothesis corresponding to *P*_(*i*)_. Define

k=max⁡{i:P(i)≤imq∗}
 MathType@MTEF@5@5@+=feaafiart1ev1aaatCvAUfKttLearuWrP9MDH5MBPbIqV92AaeXatLxBI9gBaebbnrfifHhDYfgasaacPC6xNi=xI8qiVKYPFjYdHaVhbbf9v8qqaqFr0xc9vqFj0dXdbba91qpepeI8k8fiI+fsY=rqGqVepae9pg0db9vqaiVgFr0xfr=xfr=xc9adbaqaaeGacaGaaiaabeqaaeqabiWaaaGcbaGaem4AaSMaeyypa0JagiyBa0MaeiyyaeMaeiiEaGNaei4EaSNaemyAaKMaeiOoaOJaemiuaa1aaSbaaSqaaiabcIcaOiabdMgaPjabcMcaPaqabaGccqGHKjYOjuaGdaWcaaqaaiabdMgaPbqaaiabd2gaTbaakiabdghaXnaaCaaaleqabaGaey4fIOcaaOGaeiyFa0haaa@441B@

and reject all *H*_(*i*)_, *i *= 1, 2, ⋯, *k*. If no such *i *exists, reject no hypothesis.

## Competing interests

The author(s) declare that they have no competing interests.

## Authors' contributions

WS wrote the programs, analyzed the results and drafted the manuscript. XB, MN and ZZ helped in analysis and discussion, gave useful comments. SW and XB guided the project. All authors read and approved the final manuscript.

## Supplementary Material

Additional File 15 supplementary tables included in this study. Click here for file

Additional File 1 Correlation between Z-scores and P-values of genetic robustness for all the 1,082 miRNAs and all four types of shuffled methods at threshold level *T*_1_. For each real pre-miRNA, the robustness γ1m
 MathType@MTEF@5@5@+=feaafiart1ev1aaatCvAUfKttLearuWrP9MDH5MBPbIqV92AaeXatLxBI9gBaebbnrfifHhDYfgasaacPC6xNi=xH8viVGI8Gi=hEeeu0xXdbba9frFj0xb9qqpG0dXdb9aspeI8k8fiI+fsY=rqGqVepae9pg0db9vqaiVgFr0xfr=xfr=xc9adbaqaaeGacaGaaiaabeqaaeqabiWaaaGcbaacciGae83SdC2aa0baaSqaaiabigdaXaqaaiabd2gaTbaaaaa@3001@ is compared with that of 1,000 zero-markov shuffling sequences (a), monoshuffling sequences (b), first-markov shuffling sequences (c) and dishuffling sequences (d), and the *Z*-score and *P*-value are computed. The curve for a normal distribution of mean 0 and a standard deviation of 1 is also displayed. Click here for file

Additional File 2 *P*-value distribution of genetic robustness for the 1,082 miRNAs at threshold level *T*_1_. For each real pre-miRNA, the robustness γ1m
 MathType@MTEF@5@5@+=feaafiart1ev1aaatCvAUfKttLearuWrP9MDH5MBPbIqV92AaeXatLxBI9gBaebbnrfifHhDYfgasaacPC6xNi=xH8viVGI8Gi=hEeeu0xXdbba9frFj0xb9qqpG0dXdb9aspeI8k8fiI+fsY=rqGqVepae9pg0db9vqaiVgFr0xfr=xfr=xc9adbaqaaeGacaGaaiaabeqaaeqabiWaaaGcbaacciGae83SdC2aa0baaSqaaiabigdaXaqaaiabd2gaTbaaaaa@3001@ is compared with that of 1,000 zero-markov shuffling sequences (a), monoshuffling sequences (b), first-markov shuffling sequences (c) and dishuffling sequences (d), and the *Z*-score and *P*-value are computed. Click here for file

Additional File 3 Correlation between *Z*-scores and *P*-values of genetic robustness for all the 1,082 miRNAs and all four types of shuffled methods at threshold level *T*_1_. For each real pre-miRNA, the robustness γ1m
 MathType@MTEF@5@5@+=feaafiart1ev1aaatCvAUfKttLearuWrP9MDH5MBPbIqV92AaeXatLxBI9gBaebbnrfifHhDYfgasaacPC6xNi=xH8viVGI8Gi=hEeeu0xXdbba9frFj0xb9qqpG0dXdb9aspeI8k8fiI+fsY=rqGqVepae9pg0db9vqaiVgFr0xfr=xfr=xc9adbaqaaeGacaGaaiaabeqaaeqabiWaaaGcbaacciGae83SdC2aa0baaSqaaiabigdaXaqaaiabd2gaTbaaaaa@3001@ is compared with that of 1,000 zero-markov shuffling pseudo pre-miRNAs (a), monoshuffling pseudo pre-miRNAs (b), first-markov shuffling pseudo pre-miRNAs (c) and dishuffling pseudo pre-miRNAs (d), and the *Z*-score and *P*-value are computed. The curve for a normal distribution of mean 0 and a standard deviation of 1 is also displayed. Click here for file

Additional File 4 *P*-value distribution of genetic robustness for the 1,082 miRNAs at threshold level *T*_1_. For each real pre-miRNA, the robustness γ1m
 MathType@MTEF@5@5@+=feaafiart1ev1aaatCvAUfKttLearuWrP9MDH5MBPbIqV92AaeXatLxBI9gBaebbnrfifHhDYfgasaacPC6xNi=xH8viVGI8Gi=hEeeu0xXdbba9frFj0xb9qqpG0dXdb9aspeI8k8fiI+fsY=rqGqVepae9pg0db9vqaiVgFr0xfr=xfr=xc9adbaqaaeGacaGaaiaabeqaaeqabiWaaaGcbaacciGae83SdC2aa0baaSqaaiabigdaXaqaaiabd2gaTbaaaaa@3001@ is compared with that of 1,000 zero-markov shuffling pseudo pre-miRNAs (a), monoshuffling pseudo pre-miRNAs (b), first-markov shuffling pseudo pre-miRNAs (c) and dishuffling pseudo pre-miRNAs (d), and the *Z*-score and *P*-value are computed. Click here for file

Additional File 5 Number of miRNAs with FDR-controlled *P*-values of < 0.05, 0.01, 0.005, and 0.001 at different threshold levels. For each real pre-miRNA, the robustness γim
 MathType@MTEF@5@5@+=feaafiart1ev1aaatCvAUfKttLearuWrP9MDH5MBPbIqV92AaeXatLxBI9gBaebbnrfifHhDYfgasaacPC6xNi=xH8viVGI8Gi=hEeeu0xXdbba9frFj0xb9qqpG0dXdb9aspeI8k8fiI+fsY=rqGqVepae9pg0db9vqaiVgFr0xfr=xfr=xc9adbaqaaeGacaGaaiaabeqaaeqabiWaaaGcbaacciGae83SdC2aa0baaSqaaiabdMgaPbqaaiabd2gaTbaaaaa@306C@, *i *= 1, 2, ⋯, 9 is compared with that of 1,000 shuffling RNA sequences based on zero-markov model (a), monoshuffling RNA sequences (b), shuffling RNA sequences based on first-markov model (c), dishuffling RNA sequences (d). Click here for file

Additional File 6 Number of miRNAs with FDR-controlled *P*-values of < 0.05, 0.01, 0.005, and 0.001 at different threshold levels. For each real pre-miRNA, the robustness γim
 MathType@MTEF@5@5@+=feaafiart1ev1aaatCvAUfKttLearuWrP9MDH5MBPbIqV92AaeXatLxBI9gBaebbnrfifHhDYfgasaacPC6xNi=xH8viVGI8Gi=hEeeu0xXdbba9frFj0xb9qqpG0dXdb9aspeI8k8fiI+fsY=rqGqVepae9pg0db9vqaiVgFr0xfr=xfr=xc9adbaqaaeGacaGaaiaabeqaaeqabiWaaaGcbaacciGae83SdC2aa0baaSqaaiabdMgaPbqaaiabd2gaTbaaaaa@306C@, *i *= 1, 2, ⋯, 9 is compared with that of 1,000 zero-markov shuffling pseudo pre-miRNAs (a), monoshuffling pseudo pre-miRNAs (b), first-markov shuffling pseudo pre-miRNAs (c) and dishuffling pseudo pre-miRNAs (d). Click here for file

Additional File 7 Correlation between *Z*-scores and *P*-values of environmental robustness for all the 1,082 miRNAs. For each real pre-miRNA, the free energy is compared with that of 1,000 zero-markov shuffling sequences (a), monoshuffling sequences (b), first-markov shuffling sequences (c) and dishuffling sequences (d), and the *Z*-score and *P*-value are computed. The curve for a normal distribution of mean 0 and a standard deviation of 1 is also displayed. Click here for file

Additional File 8 *P*-value distributions of environmental robustness for the 1,082 miRNAs. For each real pre-miRNA, the free energy is compared with that of 1,000 zero-markov shuffling sequences (a), monoshuffling sequences (b), first-markov shuffling sequences (c) and dishuffling sequences (d). Click here for file

Additional File 9 Correlation between *Z*-scores and *P*-values of environmental robustness for all the 1,082 miRNAs. For each real pre-miRNA, the free energy is compared with that of 1,000 zero-markov shuffling pseudo pre-miRNAs (a), monoshuffling pseudo pre-miRNAs (b), first-markov shuffling pseudo pre-miRNAs (c) and dishuffling pseudo pre-miRNAs (d). The curve for a normal distribution of mean 0 and a standard deviation of 1 is also displayed. Click here for file

Additional File 10 *P*-value distributions of environmental robustness for the 1,082 miRNAs. For each real pre-miRNA, the free energy is compared with that of 1,000 zero-markov shuffling pseudo pre-miRNAs (a), monoshuffling pseudo pre-miRNAs (b), first-markov shuffling pseudo pre-miRNAs (c) and dishuffling pseudo pre-miRNAs (d). Click here for file

Additional File 11 Correlation between genetic and environmental robustness. Scatter plots of *Z*-scores of genetic robustness and environmental robustness. The *Z*-scores were obtained by comparing the robustness of real pre-miRNAs with that of 1,000 zero-markov shuffling sequences (a), monoshuffling sequences (b), first-markov shuffling sequences (c) and dishuffling sequences (d). Click here for file

Additional File 12 Correlation between genetic and environmental robustness. Scatter plots of Z-scores of genetic robustness and environmental robustness. The Z-scores were obtained by comparing the robustness of real pre-miRNAs with that of 1,000 zero-markov shuffling pseudo pre-miRNAs (a), monoshuffling pseudo pre-miRNAs (b), first-markov shuffling pseudo pre-miRNAs (c) and dishuffling pseudo pre-miRNAs (d). Click here for file

Additional File 13 Distribution of *Z*-score of genetic and environmental robustness. 3D histogram plots of *Z*-scores of genetic robustness and environmental robustness. The *Z*-scores were obtained by comparing the robustness of real pre-miRNAs with that of 1,000 zero-markov shuffling sequences (a), monoshuffling sequences (b), first-markov shuffling sequences (c) and dishuffling sequences (d). Click here for file

Additional File 14 Distribution of *Z*-score of genetic and environmental robustness. 3D histogram plots of *Z*-scores of genetic robustness and environmental robustness. The *Z*-scores were obtained by comparing the robustness of real pre-miRNAs with that of 1,000 zero-markov shuffling pseudo pre-miRNAs (a), monoshuffling pseudo pre-miRNAs (b), first-markov shuffling pseudo pre-miRNAs (c) and dishuffling pseudo pre-miRNAs (d). Click here for file
