# Supplementary material for: In silico genetic robustness analysis of microRNA secondary structures: potential evidence of congruent evolution in microRNA
Source: BMC Evol Biol. 2007 Nov 13;7:223. doi: 10.1186/1471-2148-7-223 (PMC2222248; doi:10.1186/1471-2148-7-223)
Supplement: Additional File 15 — supplementary tables included in this study. [file 1471-2148-7-223-S15.pdf]

# Supplementary Tables

**Table S1**

**Number of miRNAs with** FDR-controlled *P*-values of **< 0.05, 0.01, 0.005, and 0.001 at threshold level $T_1$.**

For each real pre-miRNA, the robustness $\gamma_1^m$ is compared with that of 1,000 random and four types of shuffled sequences.

| FDR-controlled level | random (%) | zero-markov (%) | mononucleotide (%) | first-markov (%) | dinucleotide (%) |
|---|---|---|---|---|---|
| *P*-value < 0.05 | 920(85.0) | 924(85.4) | 925(85.5) | 920(85.0) | 915(84.6) |
| *P*-value < 0.01 | 534(49.4) | 532(49.2) | 573(53.0) | 524(48.4) | 532(49.2) |
| *P*-value < 0.005 | 376(34.8) | 377(34.8) | 388(35.9) | 373(34.5) | 377(34.8) |
| *P*-value < 0.001 | 268(24.8) | 257(23.8) | 274(25.3) | 267(24.7) | 268(24.8) |

**Table S2**

**Number of miRNAs with** FDR-controlled *P*-values of **< 0.05, 0.01, 0.005, and 0.001 at threshold level $T_1$.**

For each real pre-miRNA, the robustness $\gamma_1^m$ is compared with that of 1,000 random and four types of shuffled pseudo pre-miRNAs.

| FDR-controlled level | random (%) | zero-markov (%) | mononucleotide (%) | first-markov (%) | dinucleotide (%) |
|---|---|---|---|---|---|
| *P*-value < 0.05 | 787(72.7) | 812(75.0) | 819(75.7) | 815(75.3) | 820(75.8) |
| *P*-value < 0.01 | 320(29.6) | 378(34.9) | 385(35.6) | 323(29.9) | 367(33.9) |
| *P*-value < 0.005 | 167(15.4) | 175(16.2) | 191(17.7) | 187(17.3) | 169(15.6) |
| *P*-value < 0.001 | 167(15.4) | 175(16.2) | 191(17.7) | 187(17.3) | 169(15.6) |

**Table S3**

**Number of miRNAs with** FDR-controlled *P*-values of **< 0.05, 0.01, 0.005, and 0.001.**

For each real pre-miRNA, the free energy is compared with that of 1,000 random and four types of shuffled sequences.

| FDR-controlled level | random (%) | zero-markov (%) | mononucleotide (%) | first-markov (%) | dinucleotide (%) |
|---|---|---|---|---|---|
| *P*-value < 0.05 | 917(84.8) | 1021(94.4) | 1050(97.0) | 1021(94.4) | 1040(96.1) |
| *P*-value < 0.01 | 732(67.7) | 880(81.3) | 1001(92.5) | 897(82.9) | 958(88.5) |
| *P*-value < 0.005 | 605(55.9) | 789(72.9) | 949(87.7) | 798(73.8) | 904(83.5) |
| *P*-value < 0.001 | 445(41.1) | 558(51.6) | 779(72.0) | 587(54.3) | 701(64.8) |

**Table S4**

**Number of miRNAs with FDR-controlled *P*-values of < 0.05, 0.01, 0.005, and 0.001.**

For each real pre-miRNA, the free energy is compared with that of 1,000 random and four types of shuffled pseudo pre-miRNAs.

| FDR-controlled level | random (%) | zero-markov (%) | mononucleotide (%) | first-markov (%) | dinucleotide (%) |
|---|---|---|---|---|---|
| *P*-value < 0.05 | 904(83.5) | 1015(93.8) | 1041(96.2) | 1016(93.9) | 1026(94.8) |
| *P*-value < 0.01 | 697(64.4) | 830(76.7) | 970(89.6) | 837(77.4) | 921(85.1) |
| *P*-value < 0.005 | 566(52.3) | 740(68.4) | 919(84.9) | 748(69.1) | 816(75.4) |
| *P*-value < 0.001 | 398(36.8) | 512(47.3) | 716(66.2) | 526(48.6) | 642(59.3) |

**Table S5**

**Number of miRNAs with both genetic robustness and thermodynamic stability at FDR-controlled *P*-values of < 0.05, 0.01, 0.005, and 0.001.**

For each real pre-miRNA, the robustness $\gamma_1^m$ and the free energy are compared with that of 1,000 random and four types of shuffled sequences, respectively.

| FDR-controlled level | random (%) | zero-markov (%) | mononucleotide (%) | first-markov (%) | dinucleotide (%) |
|---|---|---|---|---|---|
| *P*-value < 0.05 | 789(72.9) | 874(80.8) | 899(83.1) | 870(80.4) | 883(81.6) |
| *P*-value < 0.01 | 424(39.2) | 477(44.1) | 531(49.1) | 471(43.5) | 505(46.7) |
| *P*-value < 0.005 | 309(28.6) | 345(31.9) | 394(36.4) | 337(31.1) | 388(35.9) |
| *P*-value < 0.001 | 130(12.0) | 151(14.0) | 201(18.6) | 158(14.6) | 185(17.1) |

**Table S6**

**Number of miRNAs with both genetic robustness and thermodynamic stability at FDR-controlled *P*-values of < 0.05, 0.01, 0.005, and 0.001.**

For each real pre-miRNA, the robustness and the free energy $\gamma_1^m$ are compared with that of 1,000 random and four types of shuffled pseudo pre-miRNAs, respectively.

| FDR-controlled level | random (%) | zero-markov (%) | mononucleotide (%) | first-markov (%) | dinucleotide (%) |
|---|---|---|---|---|---|
| *P*-value < 0.05 | 662(61.2) | 764(70.6) | 789(72.9) | 766(70.8) | 784(72.5) |
| *P*-value < 0.01 | 248(22.9) | 303(28.0) | 352(32.5) | 285(26.3) | 326(30.1) |
| *P*-value < 0.005 | 152(14.0) | 186(17.2) | 229(21.2) | 191(17.7) | 200(18.5) |
| *P*-value < 0.001 | 79(7.3) | 86(7.9) | 130(12.0) | 97(9.0) | 103(9.5) |
