# Supplementary figures and images for: In silico genetic robustness analysis of microRNA secondary structures: potential evidence of congruent evolution in microRNA
Source: BMC Evol Biol. 2007 Nov 13;7:223. doi: 10.1186/1471-2148-7-223 (PMC2222248; doi:10.1186/1471-2148-7-223)

(a)

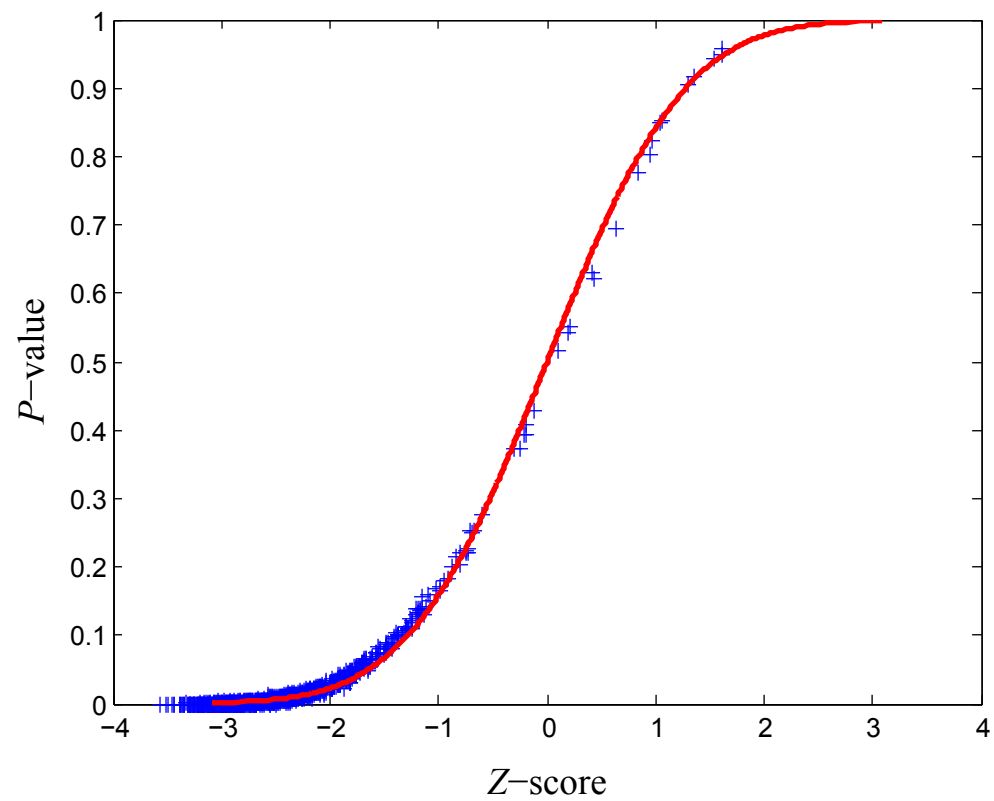

(b)

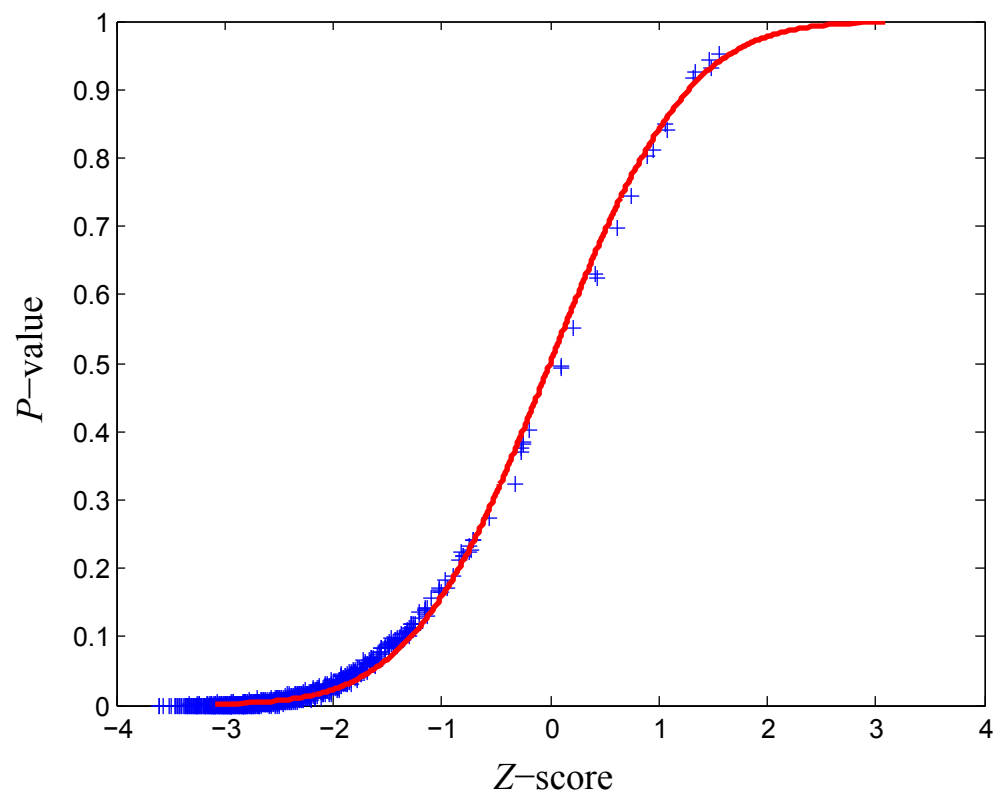

(c)

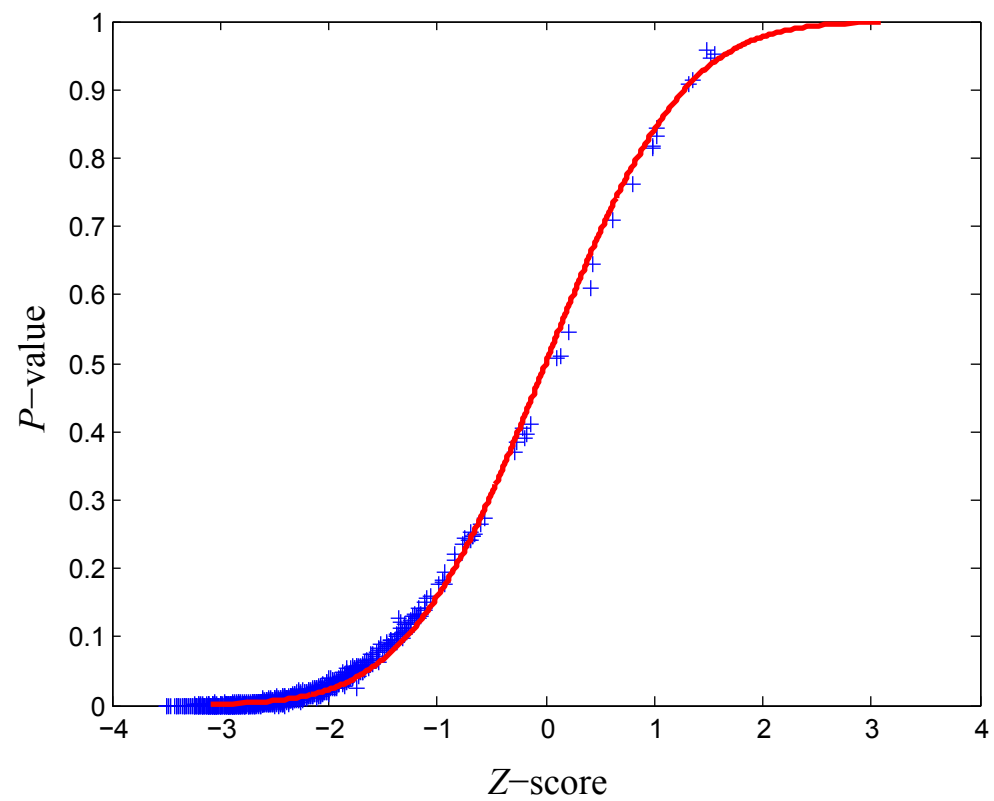

(d)

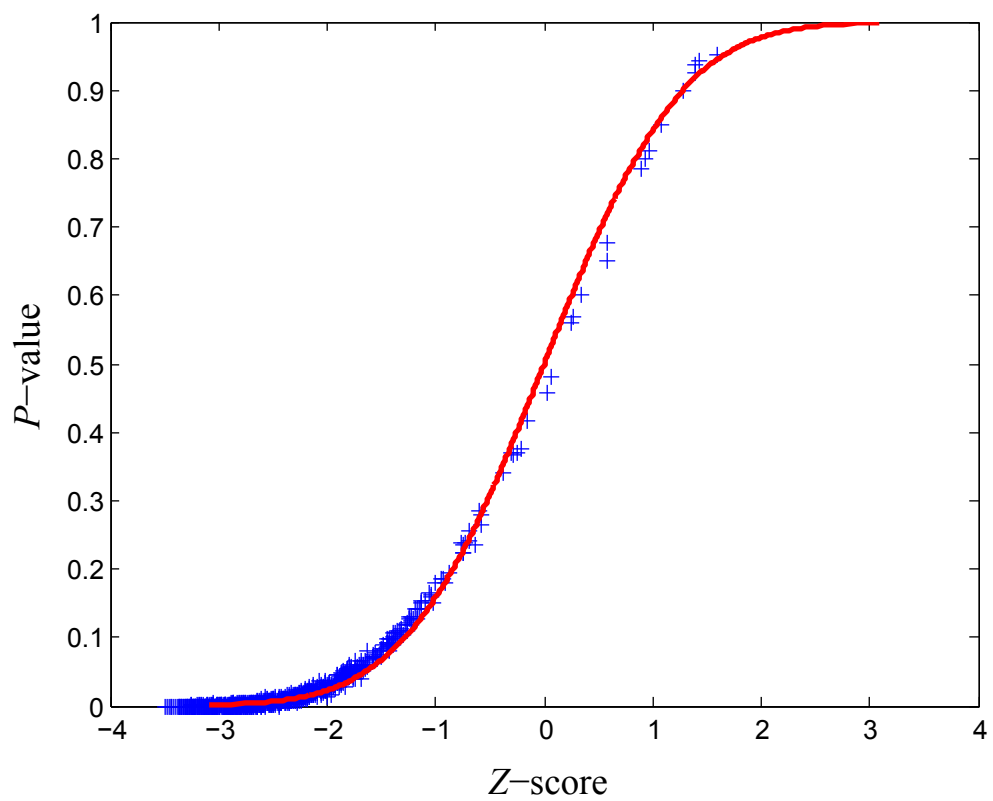

Supplement: Additional File 1 — Correlation between Z-scores and P-values of genetic robustness for all the 1,082 miRNAs and all four types of shuffled methods at threshold level T1. For each real pre-miRNA, the robustness γ1m MathType@MTEF@5@5@+=feaafiart1ev1aaatCvAUfKttLearuWrP9MDH5MBPbIqV92AaeXatLxBI9gBaebbnrfifHhDYfgasaacPC6xNi=xH8viVGI8Gi=hEeeu0xXdbba9frFj0xb9qqpG0dXdb9aspeI8k8fiI+fsY=rqGqVepae9pg0db9vqaiVgFr0xfr=xfr=xc9adbaqaaeGacaGaaiaabeqaaeqabiWaaaGcbaacciGae83SdC2aa0baaSqaaiabigdaXaqaaiabd2gaTbaaaaa@3001@ is compared with that of 1,000 zero-markov shuffling sequences (a), monoshuffling sequences (b), first-markov shuffling sequences (c) and dishuffling sequences (d), and the Z-score and P-value are computed. The curve for a normal distribution of mean 0 and a standard deviation of 1 is also displayed. [file 1471-2148-7-223-S1.pdf]

(a)

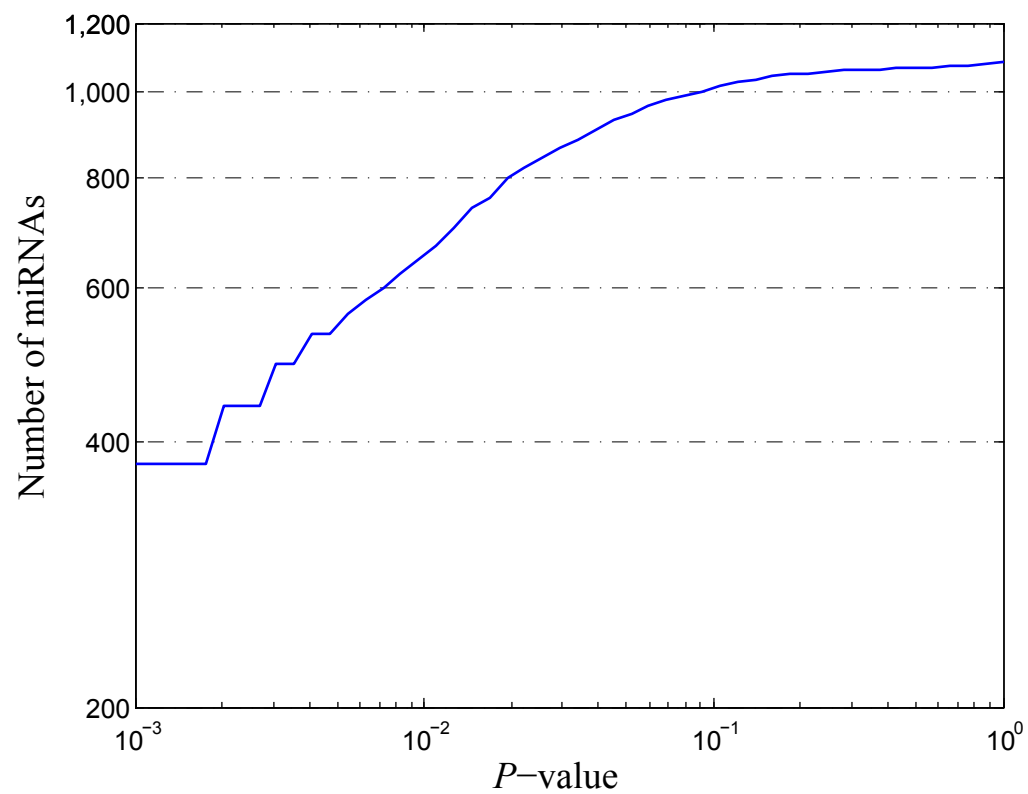

(b)

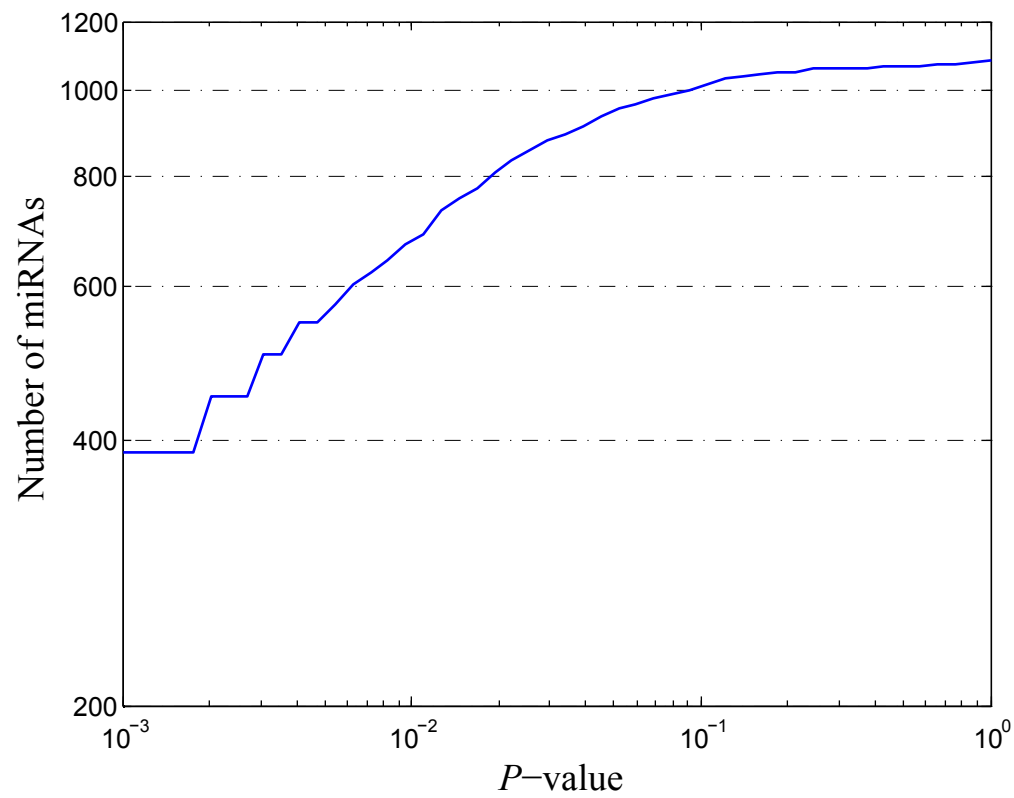

(c)

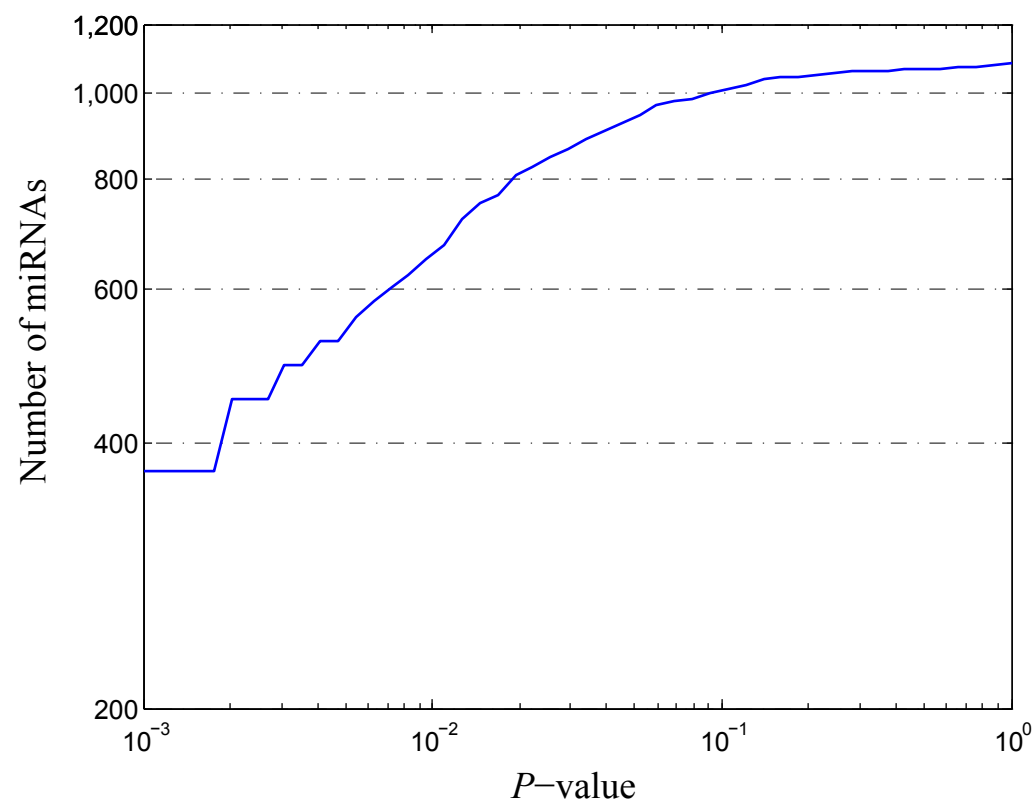

(d)

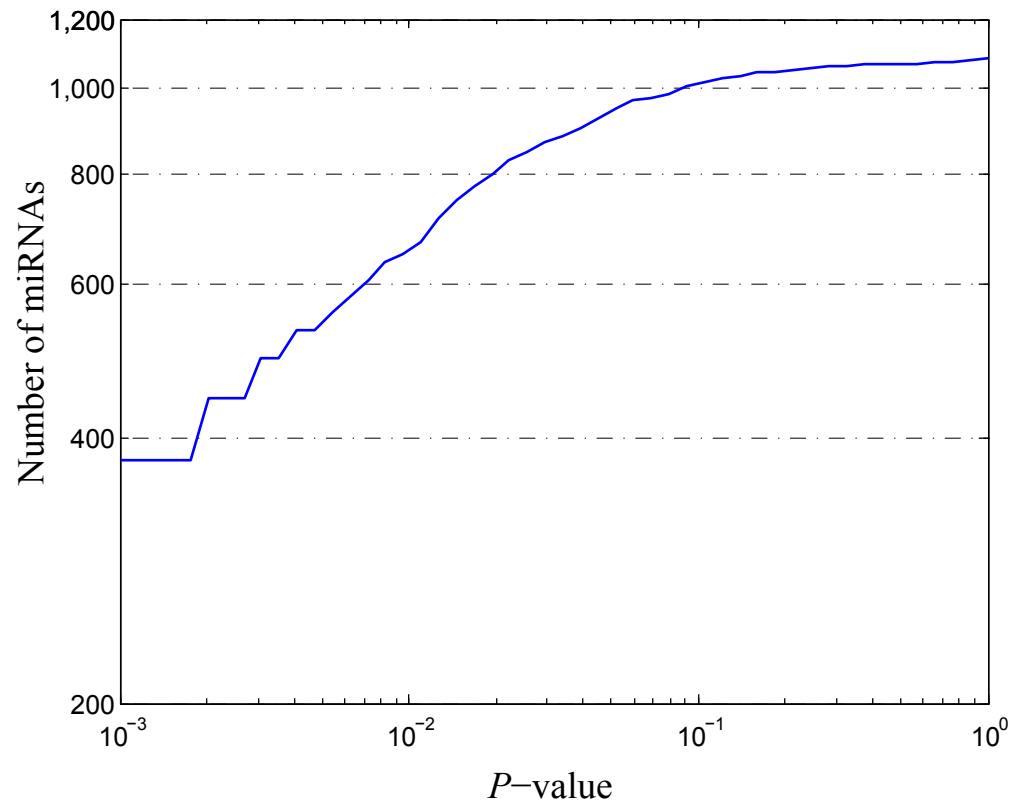

Supplement: Additional File 2 — P-value distribution of genetic robustness for the 1,082 miRNAs at threshold level T1. For each real pre-miRNA, the robustness γ1m MathType@MTEF@5@5@+=feaafiart1ev1aaatCvAUfKttLearuWrP9MDH5MBPbIqV92AaeXatLxBI9gBaebbnrfifHhDYfgasaacPC6xNi=xH8viVGI8Gi=hEeeu0xXdbba9frFj0xb9qqpG0dXdb9aspeI8k8fiI+fsY=rqGqVepae9pg0db9vqaiVgFr0xfr=xfr=xc9adbaqaaeGacaGaaiaabeqaaeqabiWaaaGcbaacciGae83SdC2aa0baaSqaaiabigdaXaqaaiabd2gaTbaaaaa@3001@ is compared with that of 1,000 zero-markov shuffling sequences (a), monoshuffling sequences (b), first-markov shuffling sequences (c) and dishuffling sequences (d), and the Z-score and P-value are computed. [file 1471-2148-7-223-S2.pdf]

(a)

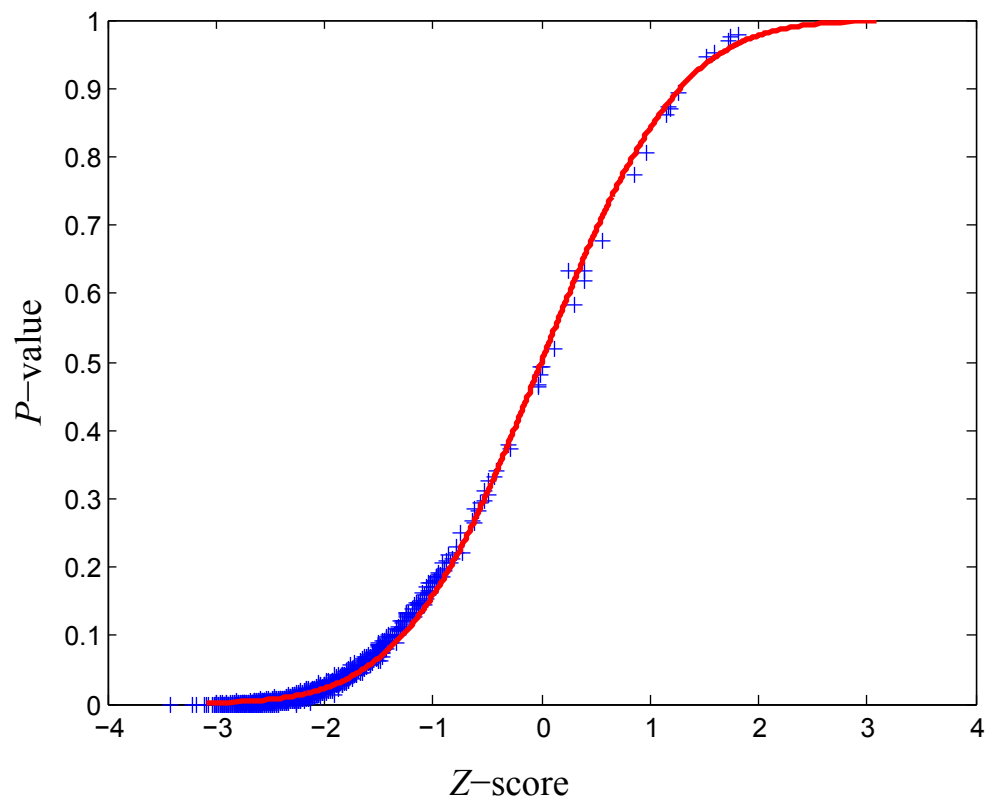

(b)

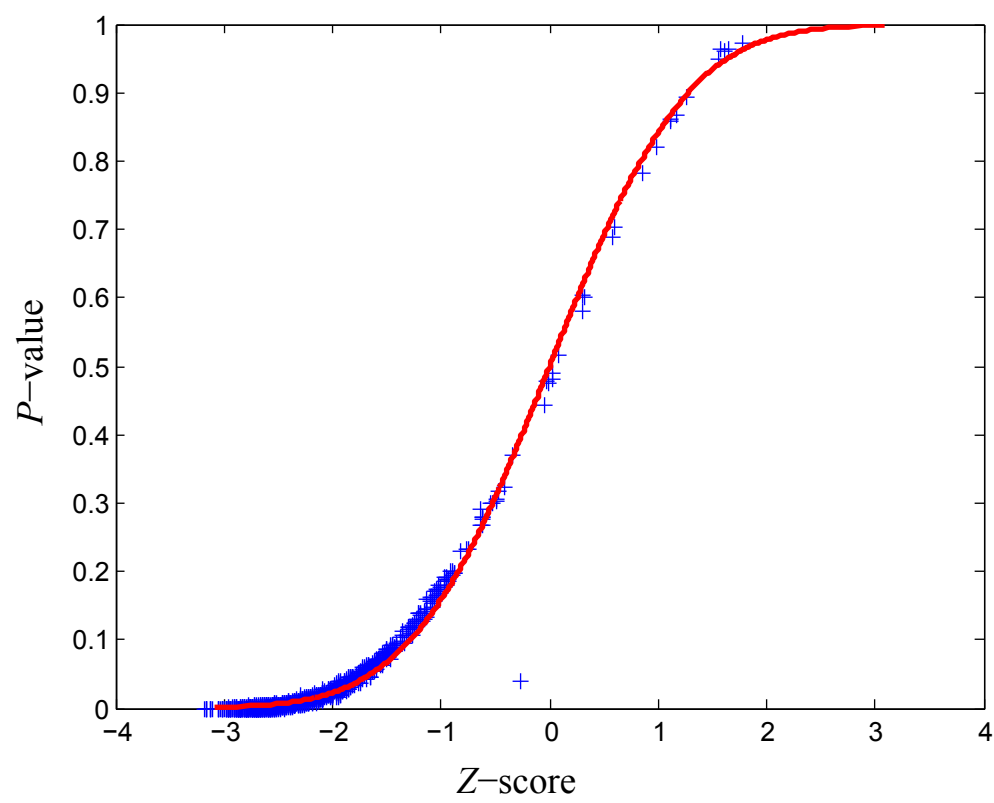

(c)

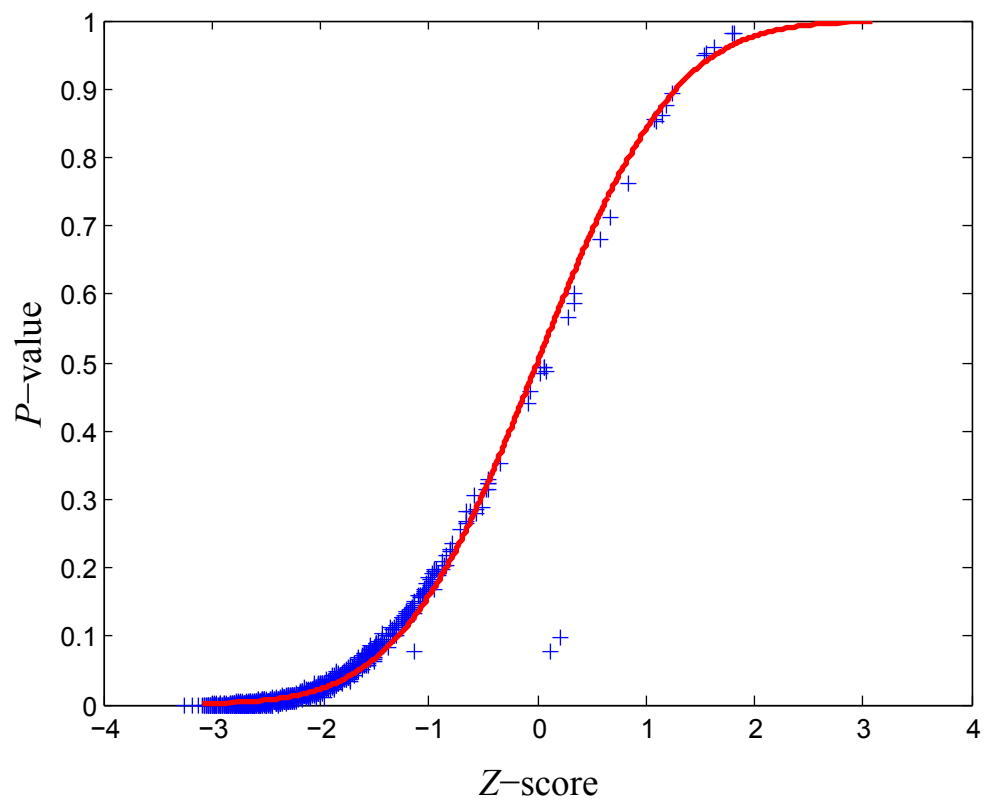

(d)

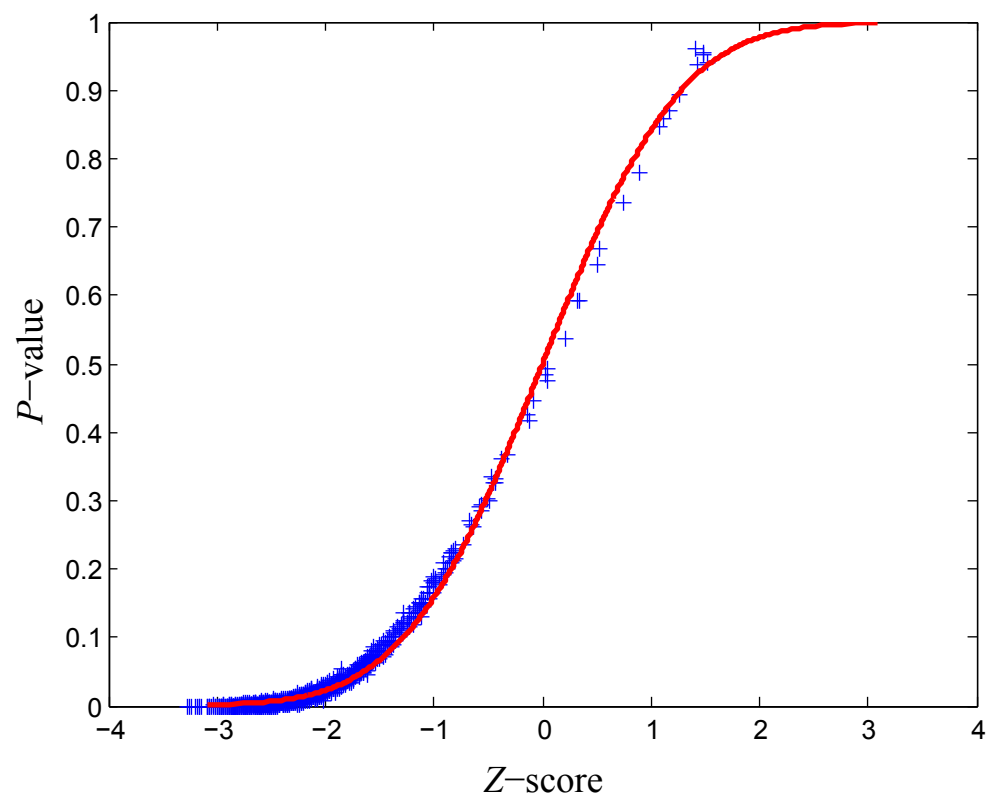

Supplement: Additional File 3 — Correlation between Z-scores and P-values of genetic robustness for all the 1,082 miRNAs and all four types of shuffled methods at threshold level T1. For each real pre-miRNA, the robustness γ1m MathType@MTEF@5@5@+=feaafiart1ev1aaatCvAUfKttLearuWrP9MDH5MBPbIqV92AaeXatLxBI9gBaebbnrfifHhDYfgasaacPC6xNi=xH8viVGI8Gi=hEeeu0xXdbba9frFj0xb9qqpG0dXdb9aspeI8k8fiI+fsY=rqGqVepae9pg0db9vqaiVgFr0xfr=xfr=xc9adbaqaaeGacaGaaiaabeqaaeqabiWaaaGcbaacciGae83SdC2aa0baaSqaaiabigdaXaqaaiabd2gaTbaaaaa@3001@ is compared with that of 1,000 zero-markov shuffling pseudo pre-miRNAs (a), monoshuffling pseudo pre-miRNAs (b), first-markov shuffling pseudo pre-miRNAs (c) and dishuffling pseudo pre-miRNAs (d), and the Z-score and P-value are computed. The curve for a normal distribution of mean 0 and a standard deviation of 1 is also displayed. [file 1471-2148-7-223-S3.pdf]

(a)

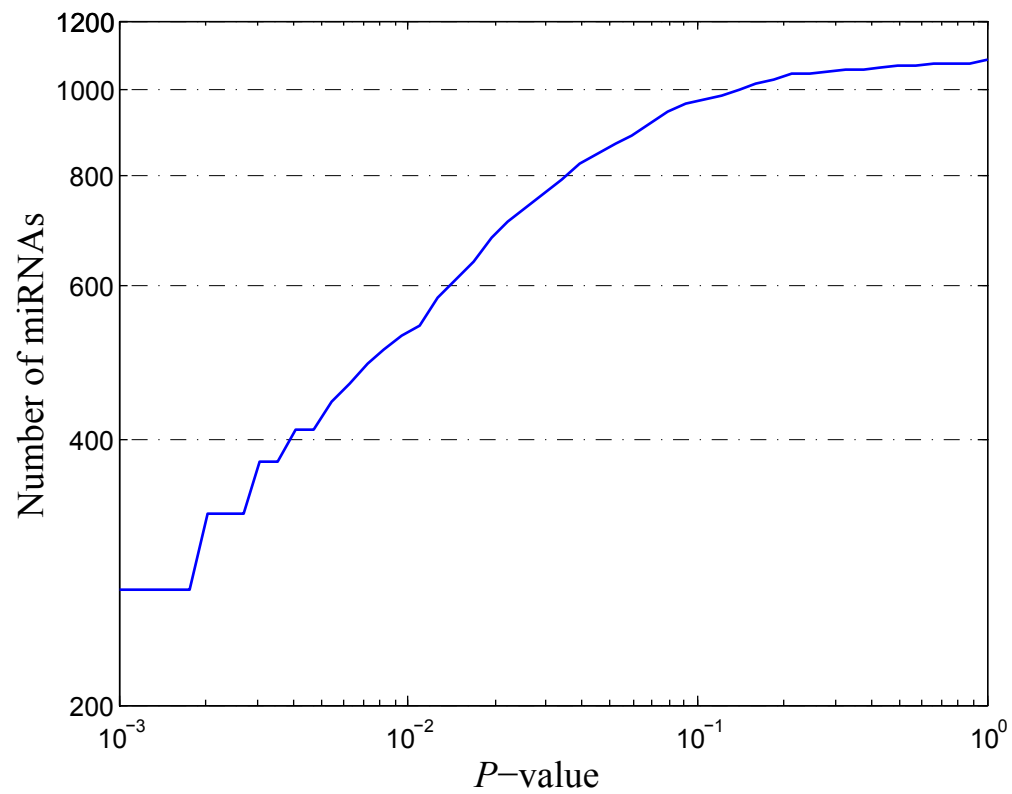

(b)

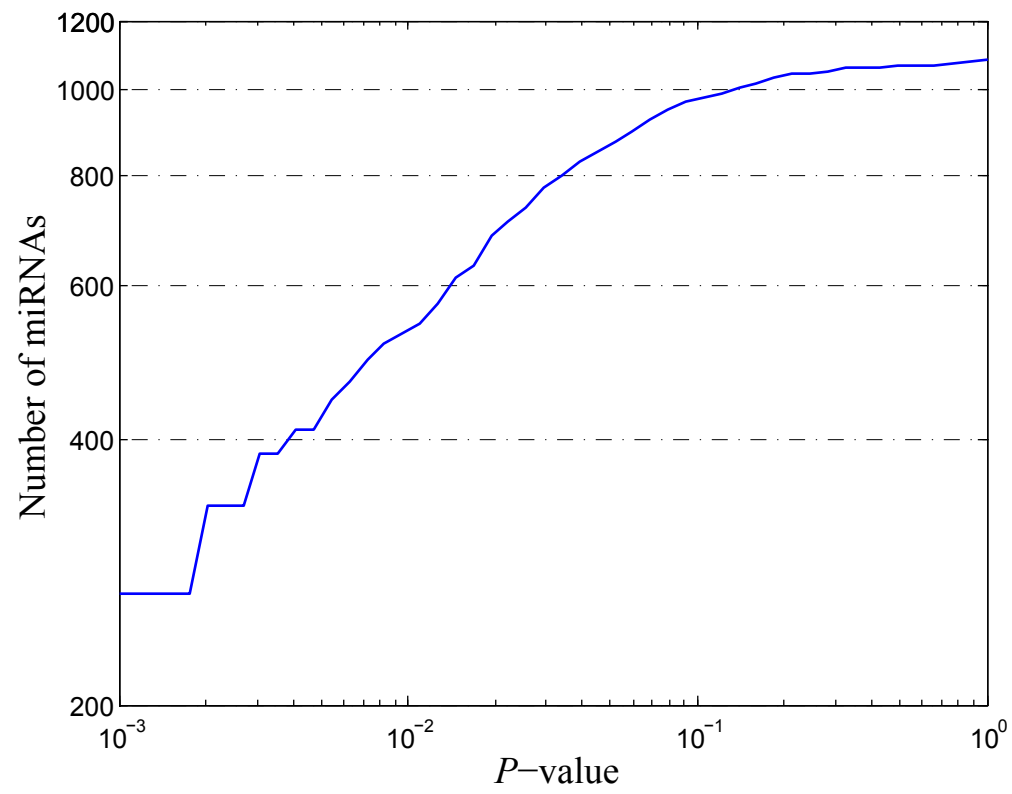

(c)

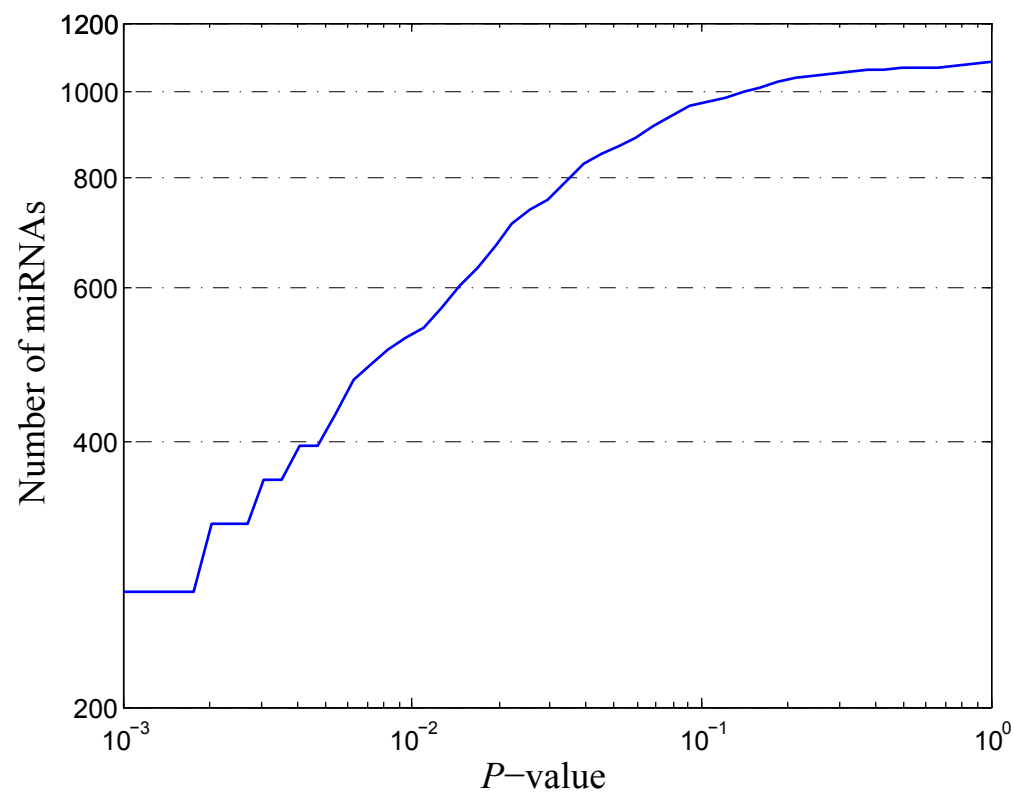

(d)

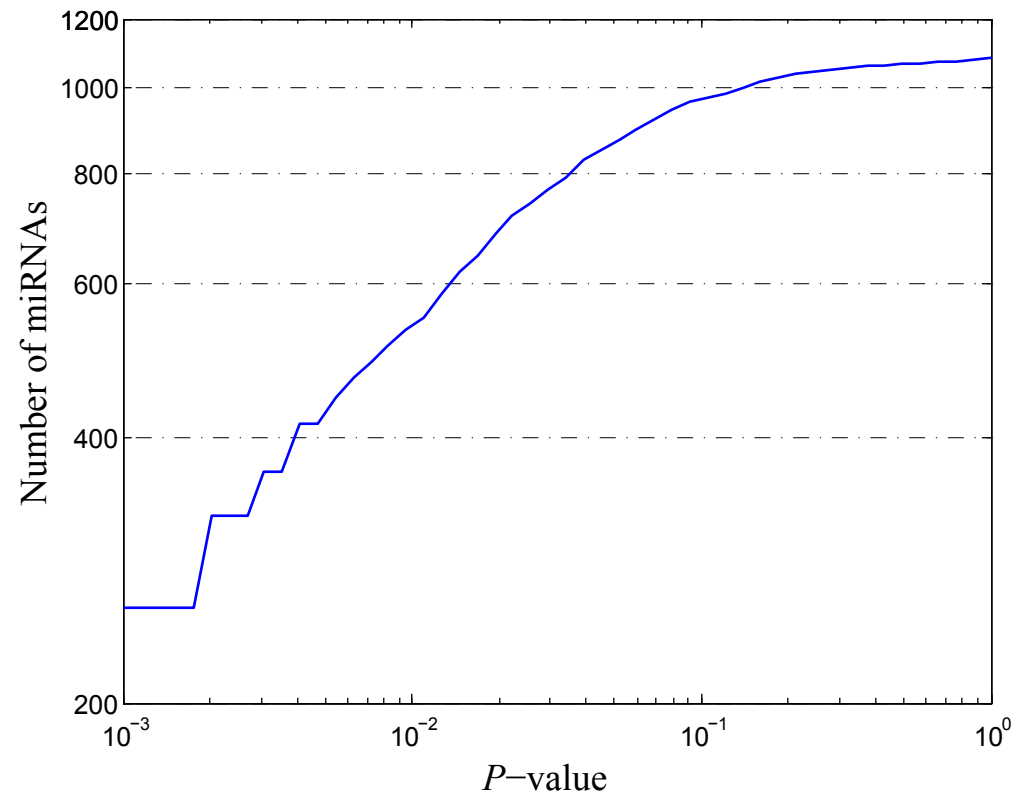

Supplement: Additional File 4 — P-value distribution of genetic robustness for the 1,082 miRNAs at threshold level T1. For each real pre-miRNA, the robustness γ1m MathType@MTEF@5@5@+=feaafiart1ev1aaatCvAUfKttLearuWrP9MDH5MBPbIqV92AaeXatLxBI9gBaebbnrfifHhDYfgasaacPC6xNi=xH8viVGI8Gi=hEeeu0xXdbba9frFj0xb9qqpG0dXdb9aspeI8k8fiI+fsY=rqGqVepae9pg0db9vqaiVgFr0xfr=xfr=xc9adbaqaaeGacaGaaiaabeqaaeqabiWaaaGcbaacciGae83SdC2aa0baaSqaaiabigdaXaqaaiabd2gaTbaaaaa@3001@ is compared with that of 1,000 zero-markov shuffling pseudo pre-miRNAs (a), monoshuffling pseudo pre-miRNAs (b), first-markov shuffling pseudo pre-miRNAs (c) and dishuffling pseudo pre-miRNAs (d), and the Z-score and P-value are computed. [file 1471-2148-7-223-S4.pdf]

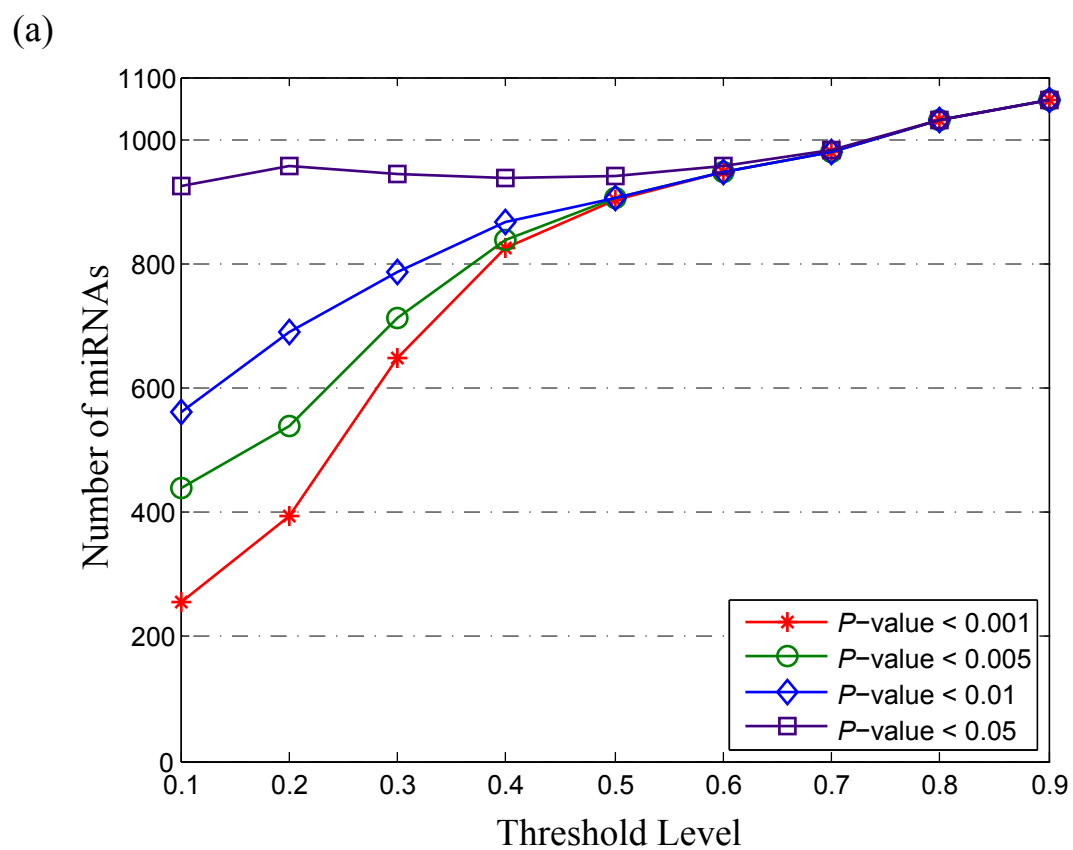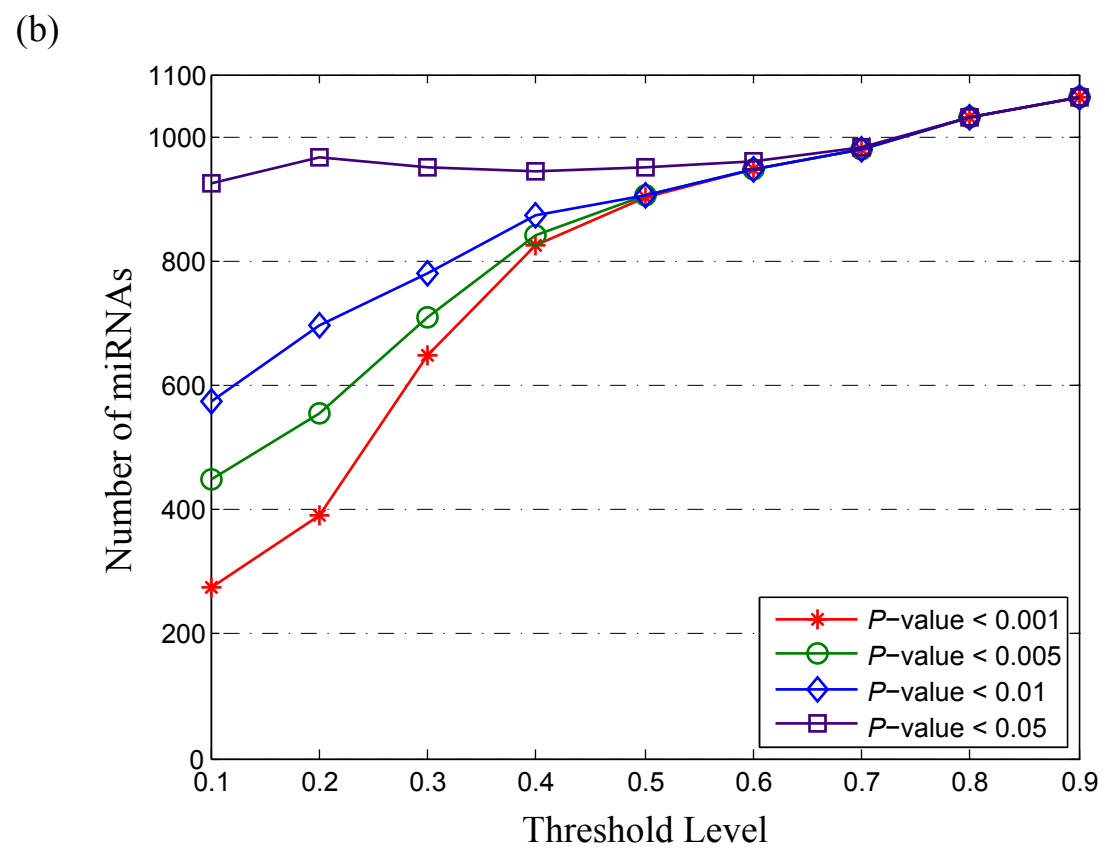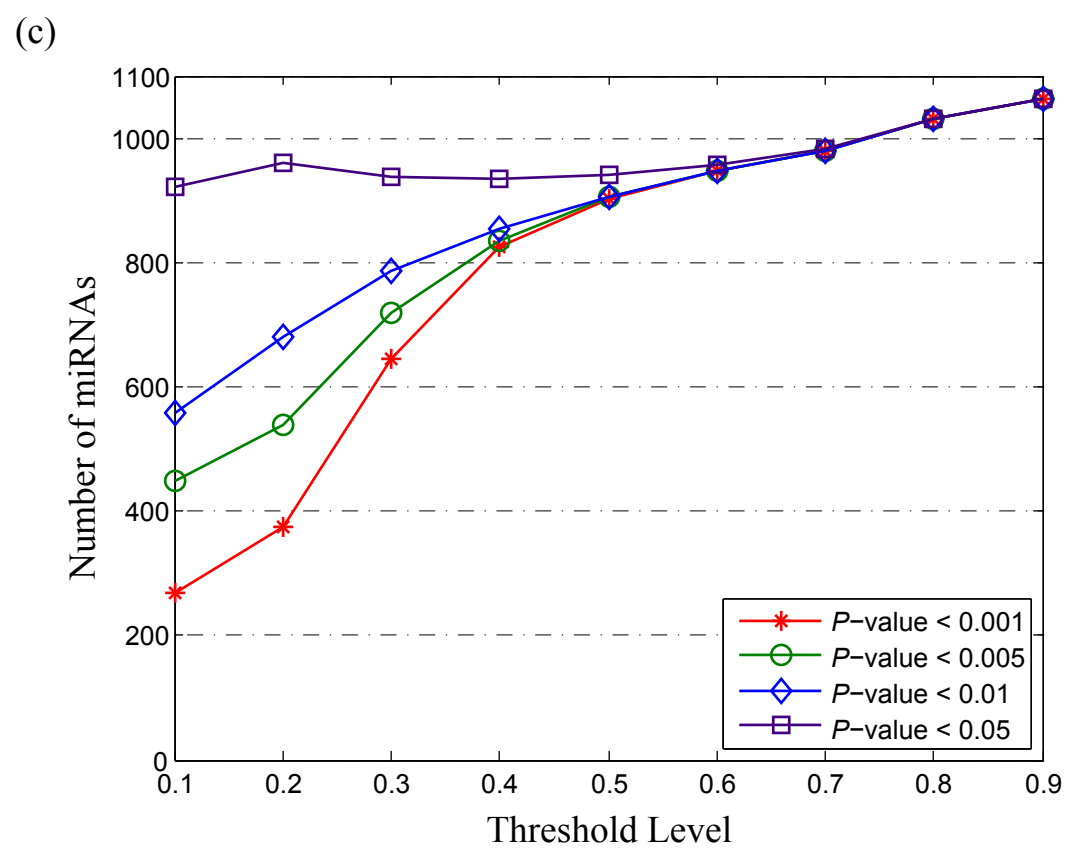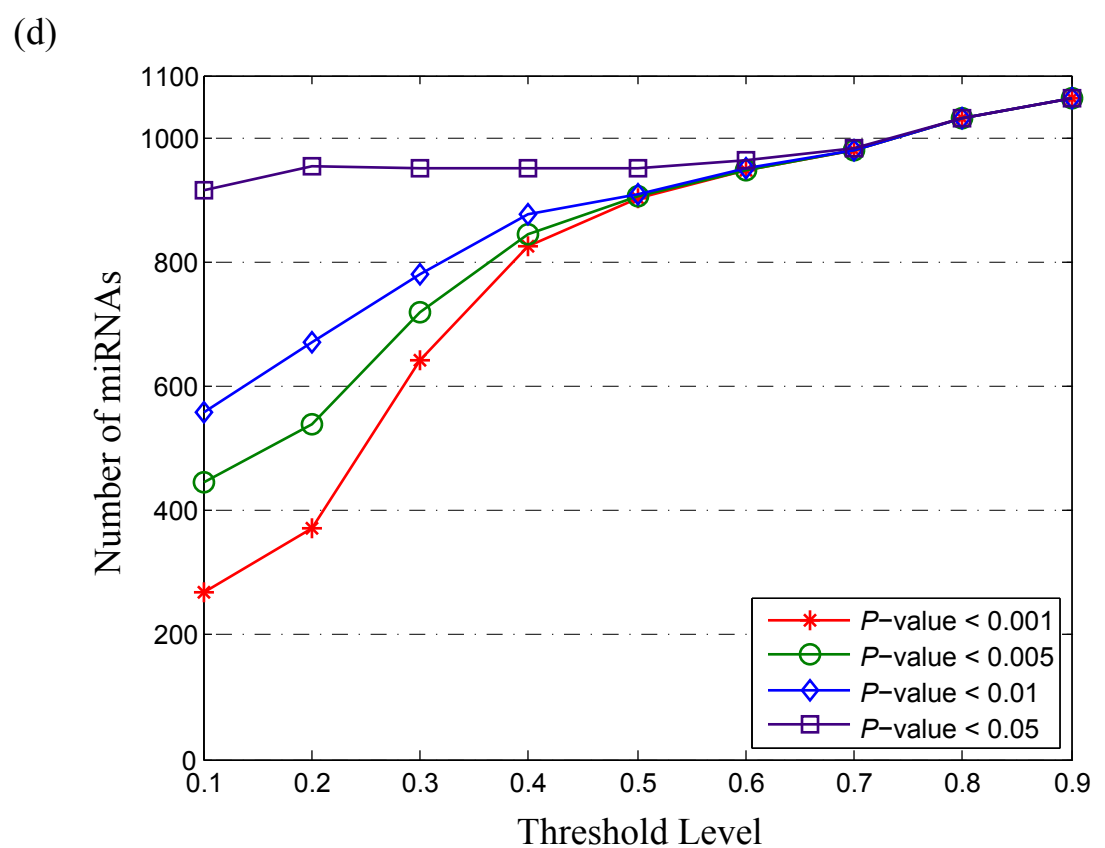

Supplement: Additional File 5 — Number of miRNAs with FDR-controlled P-values of < 0.05, 0.01, 0.005, and 0.001 at different threshold levels. For each real pre-miRNA, the robustness γim MathType@MTEF@5@5@+=feaafiart1ev1aaatCvAUfKttLearuWrP9MDH5MBPbIqV92AaeXatLxBI9gBaebbnrfifHhDYfgasaacPC6xNi=xH8viVGI8Gi=hEeeu0xXdbba9frFj0xb9qqpG0dXdb9aspeI8k8fiI+fsY=rqGqVepae9pg0db9vqaiVgFr0xfr=xfr=xc9adbaqaaeGacaGaaiaabeqaaeqabiWaaaGcbaacciGae83SdC2aa0baaSqaaiabdMgaPbqaaiabd2gaTbaaaaa@306C@, i = 1, 2, ⋯, 9 is compared with that of 1,000 shuffling RNA sequences based on zero-markov model (a), monoshuffling RNA sequences (b), shuffling RNA sequences based on first-markov model (c), dishuffling RNA sequences (d). [file 1471-2148-7-223-S5.pdf]

(a)

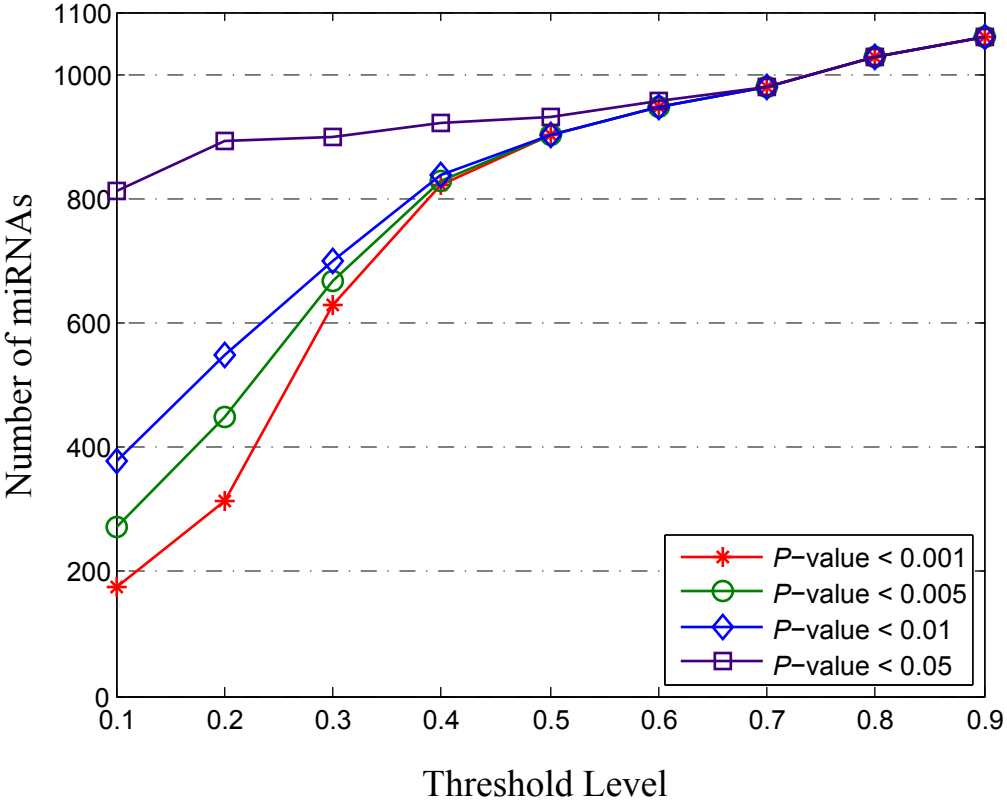

(b)

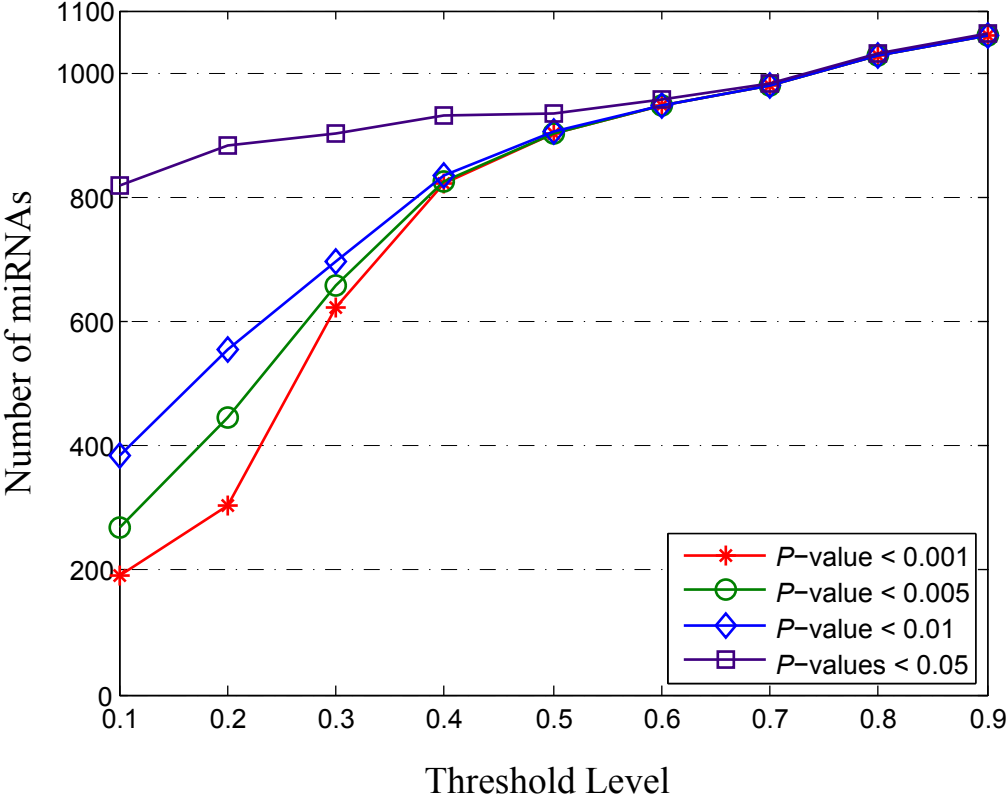

(c)

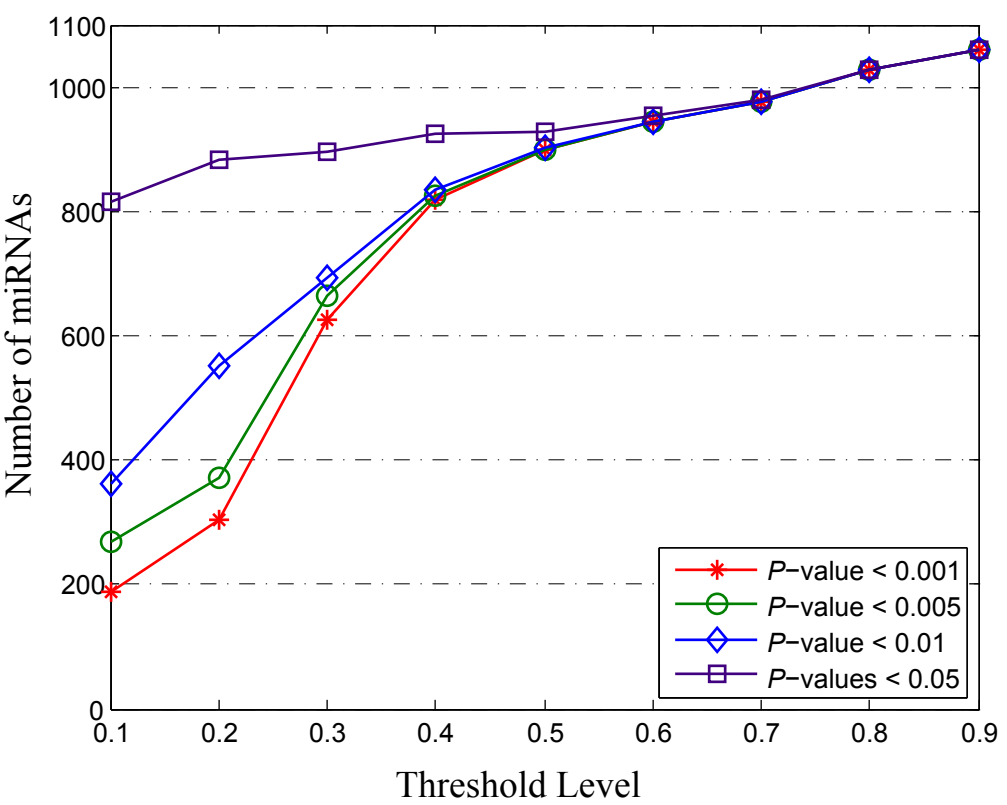

(d)

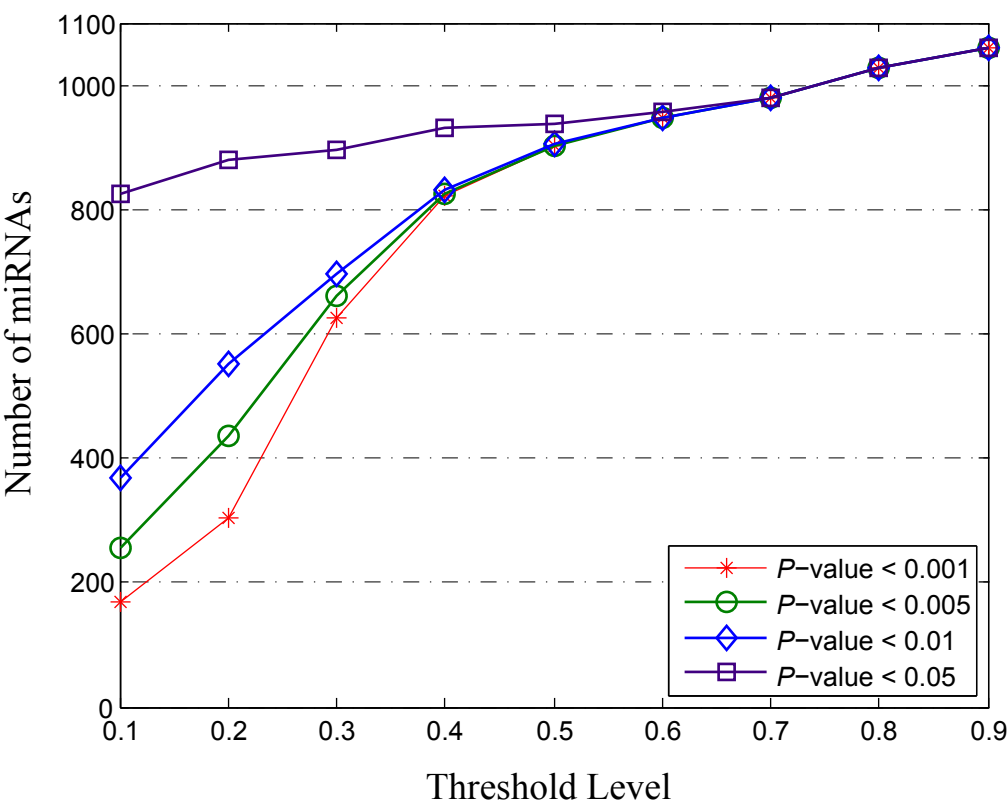

Supplement: Additional File 6 — Number of miRNAs with FDR-controlled P-values of < 0.05, 0.01, 0.005, and 0.001 at different threshold levels. For each real pre-miRNA, the robustness γim MathType@MTEF@5@5@+=feaafiart1ev1aaatCvAUfKttLearuWrP9MDH5MBPbIqV92AaeXatLxBI9gBaebbnrfifHhDYfgasaacPC6xNi=xH8viVGI8Gi=hEeeu0xXdbba9frFj0xb9qqpG0dXdb9aspeI8k8fiI+fsY=rqGqVepae9pg0db9vqaiVgFr0xfr=xfr=xc9adbaqaaeGacaGaaiaabeqaaeqabiWaaaGcbaacciGae83SdC2aa0baaSqaaiabdMgaPbqaaiabd2gaTbaaaaa@306C@, i = 1, 2, ⋯, 9 is compared with that of 1,000 zero-markov shuffling pseudo pre-miRNAs (a), monoshuffling pseudo pre-miRNAs (b), first-markov shuffling pseudo pre-miRNAs (c) and dishuffling pseudo pre-miRNAs (d). [file 1471-2148-7-223-S6.pdf]

(a)

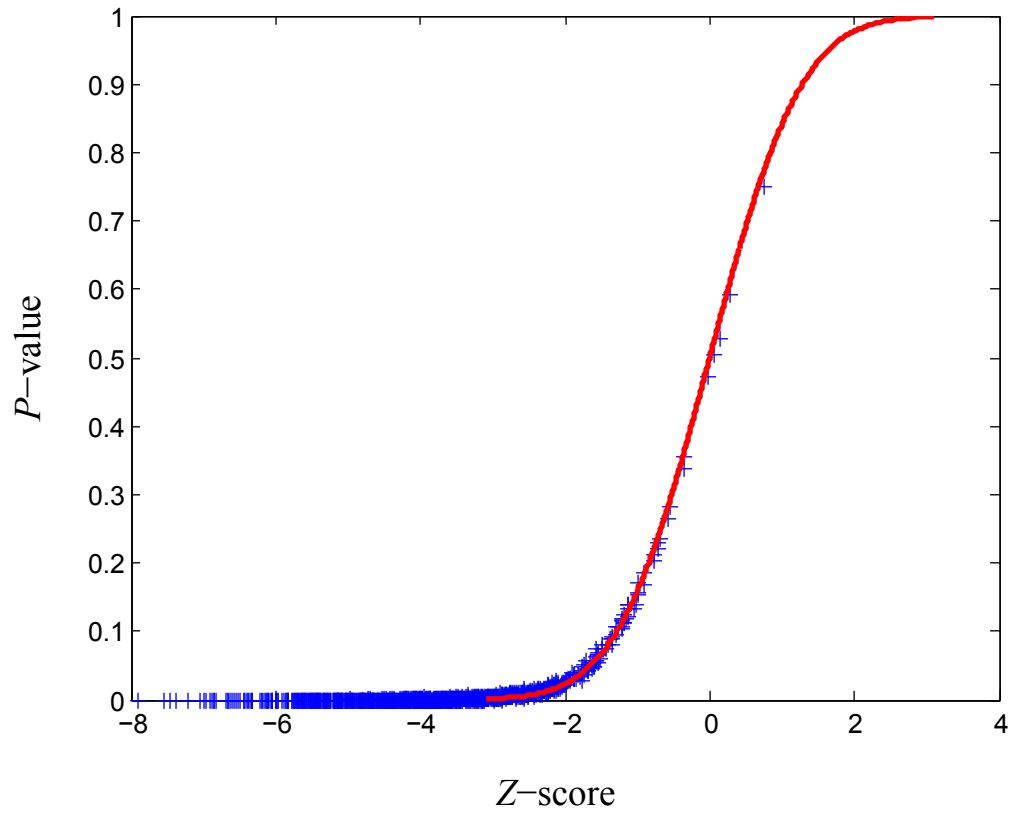

(b)

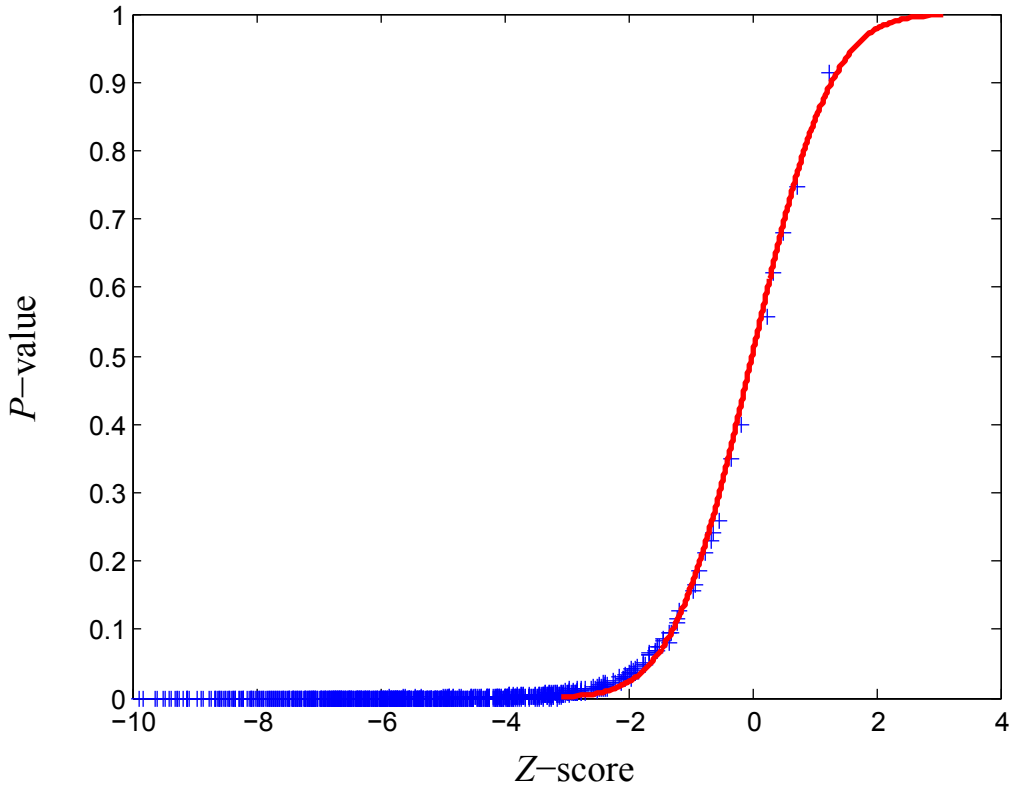

(c)

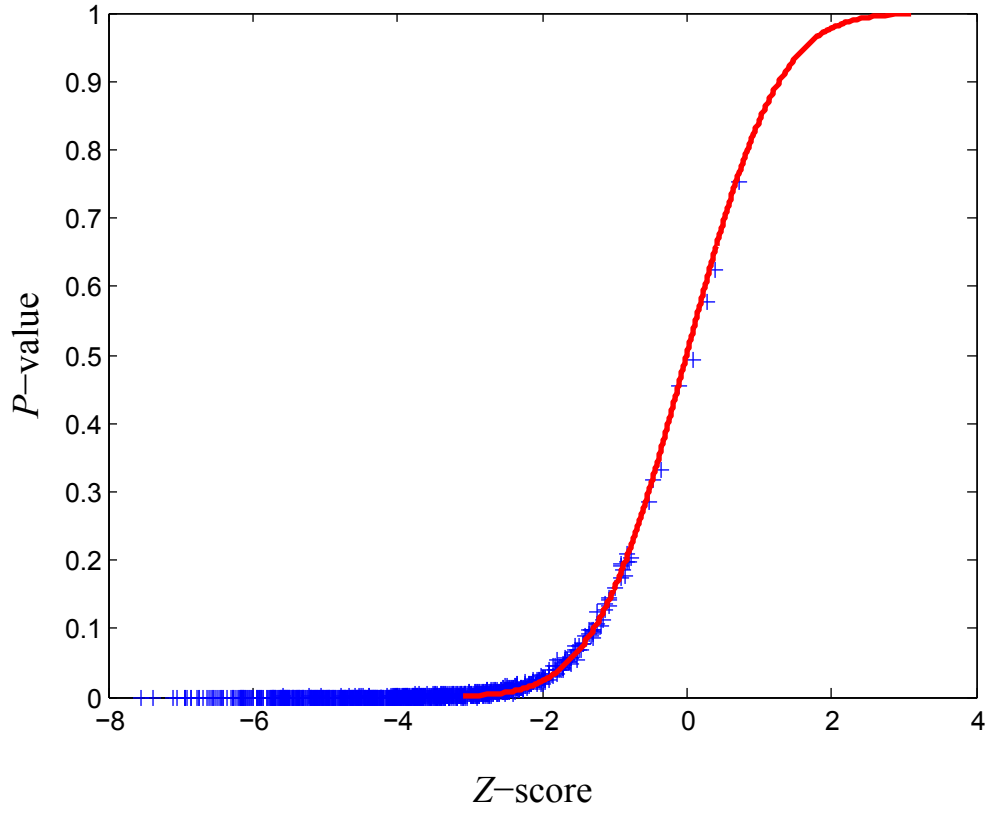

(d)

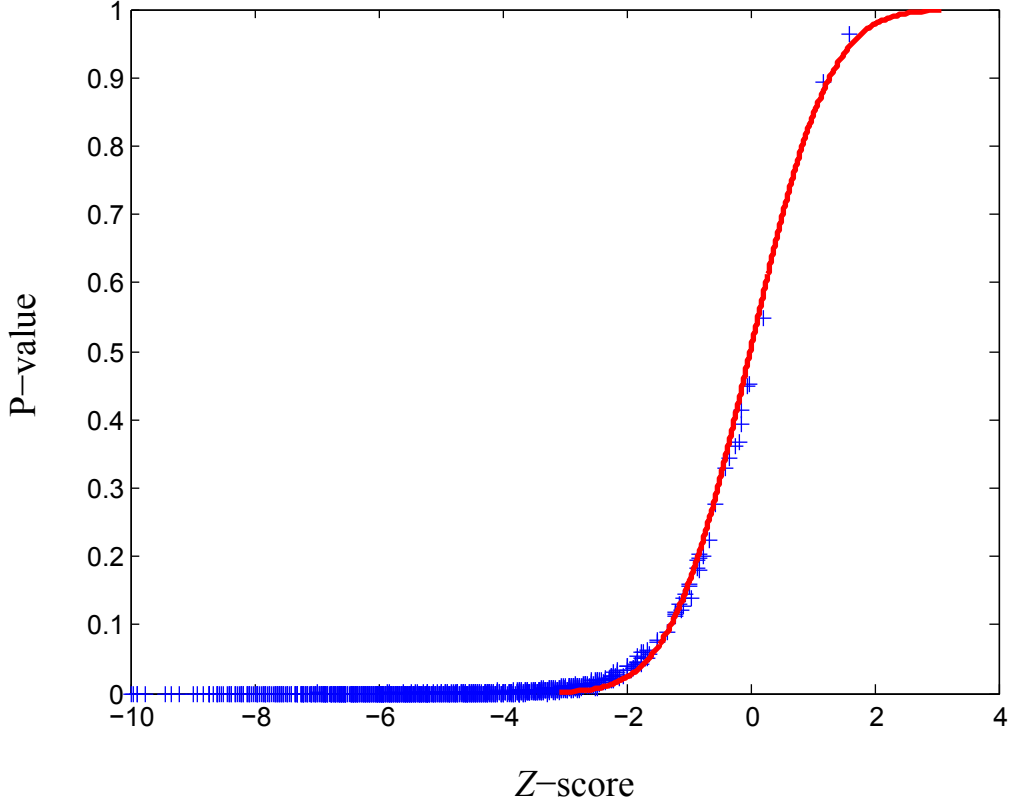

Supplement: Additional File 7 — Correlation between Z-scores and P-values of environmental robustness for all the 1,082 miRNAs. For each real pre-miRNA, the free energy is compared with that of 1,000 zero-markov shuffling sequences (a), monoshuffling sequences (b), first-markov shuffling sequences (c) and dishuffling sequences (d), and the Z-score and P-value are computed. The curve for a normal distribution of mean 0 and a standard deviation of 1 is also displayed. [file 1471-2148-7-223-S7.pdf]

(a)

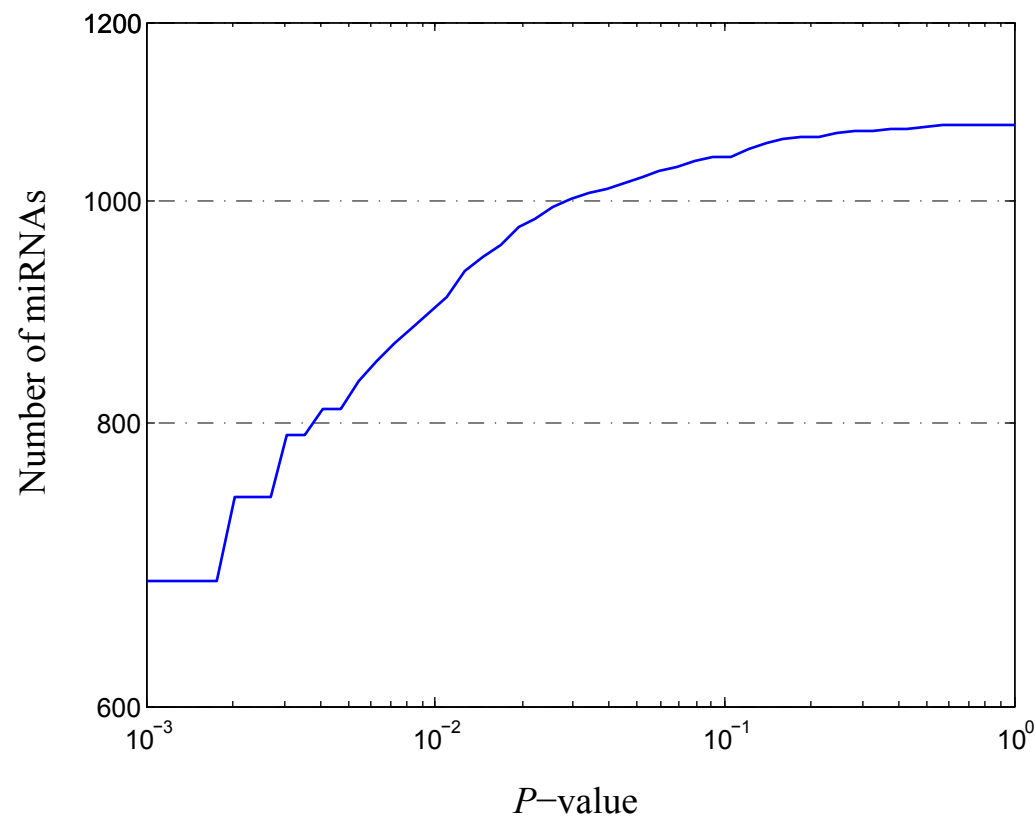

(b)

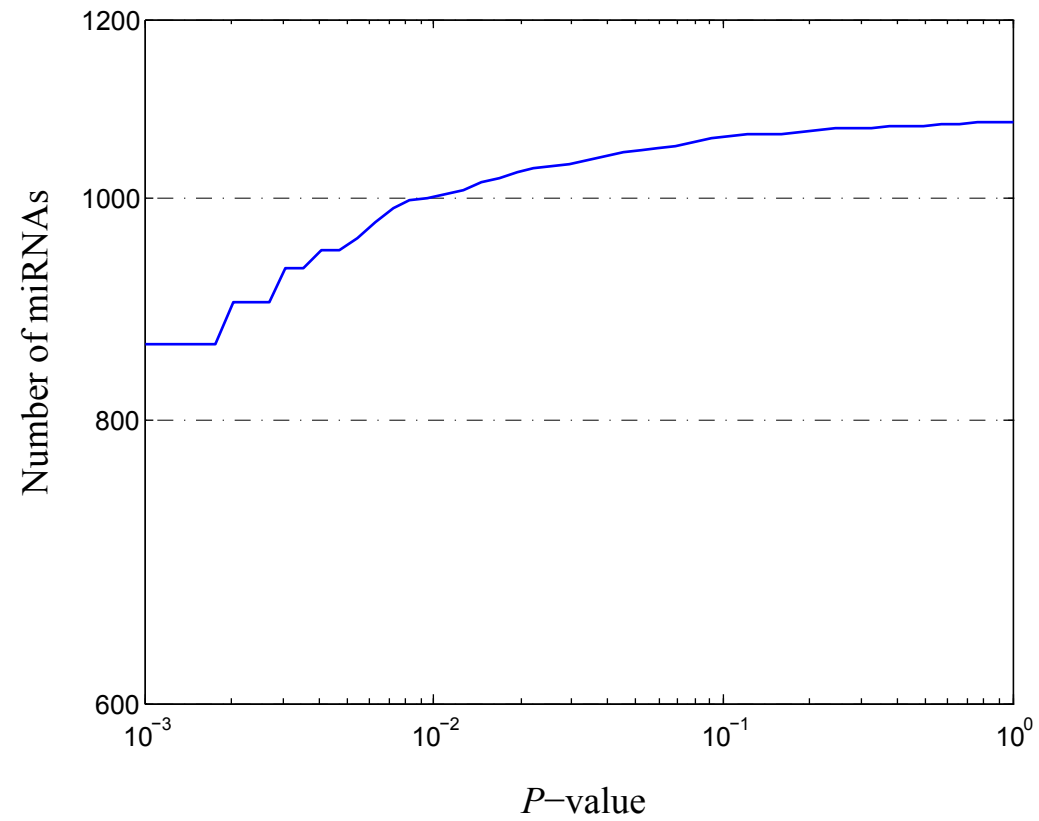

(c)

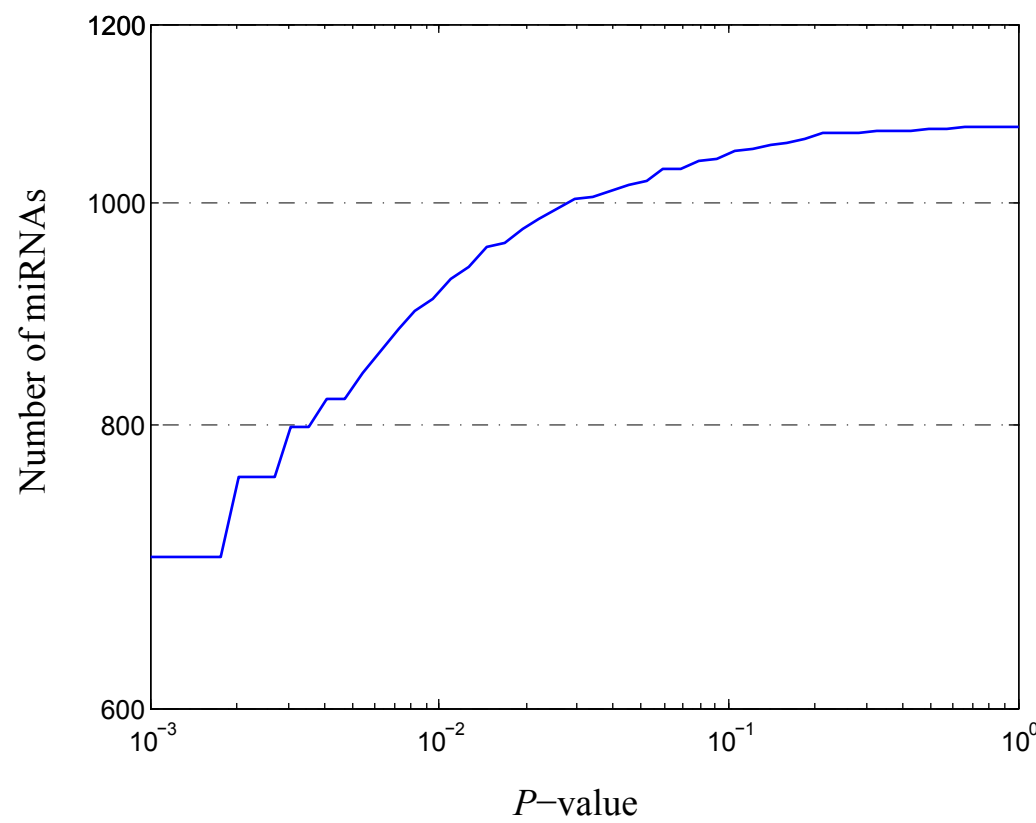

(d)

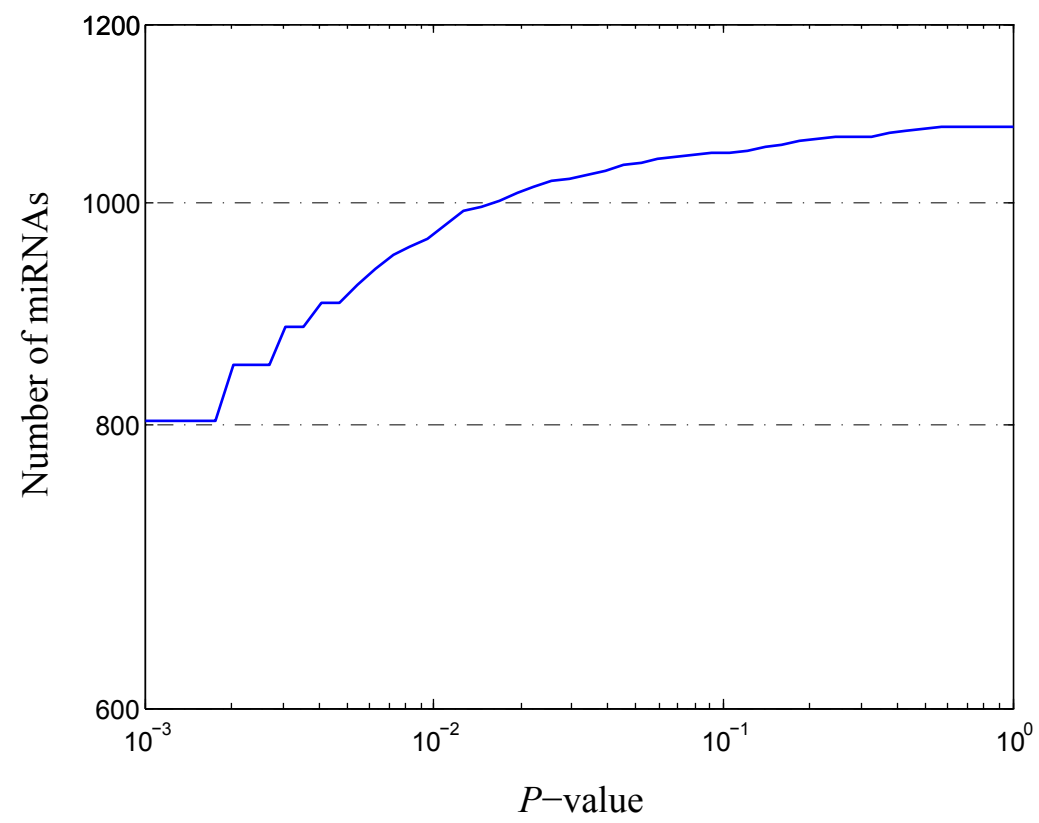

Supplement: Additional File 8 — P-value distributions of environmental robustness for the 1,082 miRNAs. For each real pre-miRNA, the free energy is compared with that of 1,000 zero-markov shuffling sequences (a), monoshuffling sequences (b), first-markov shuffling sequences (c) and dishuffling sequences (d). [file 1471-2148-7-223-S8.pdf]

(a)

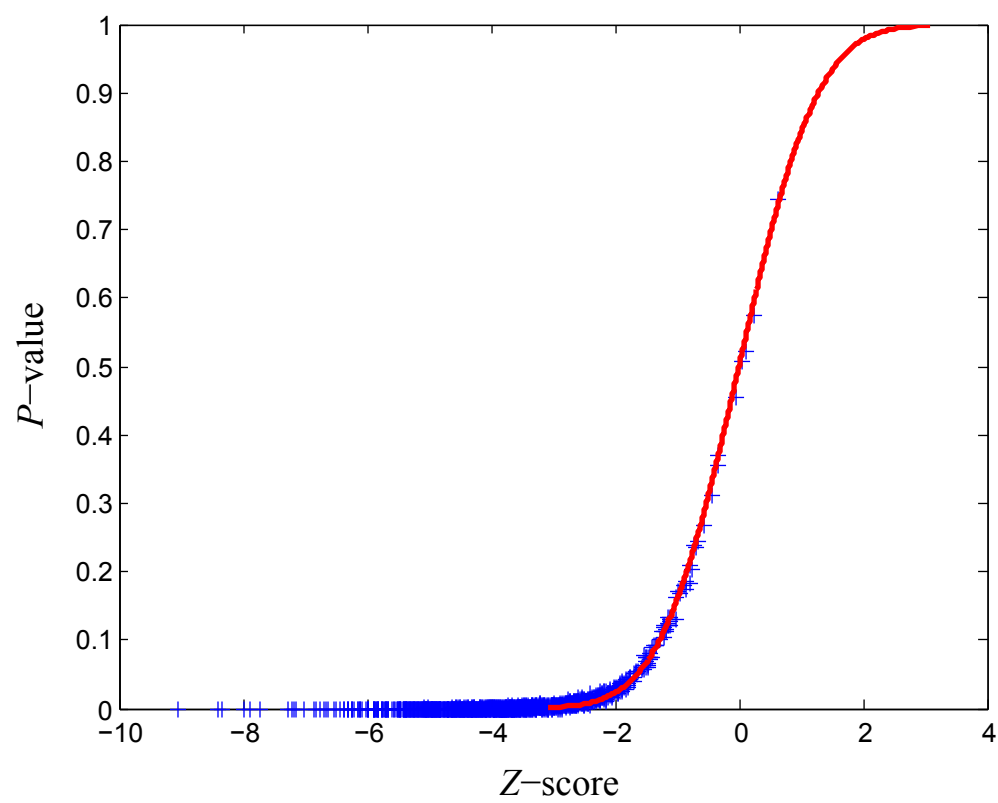

(b)

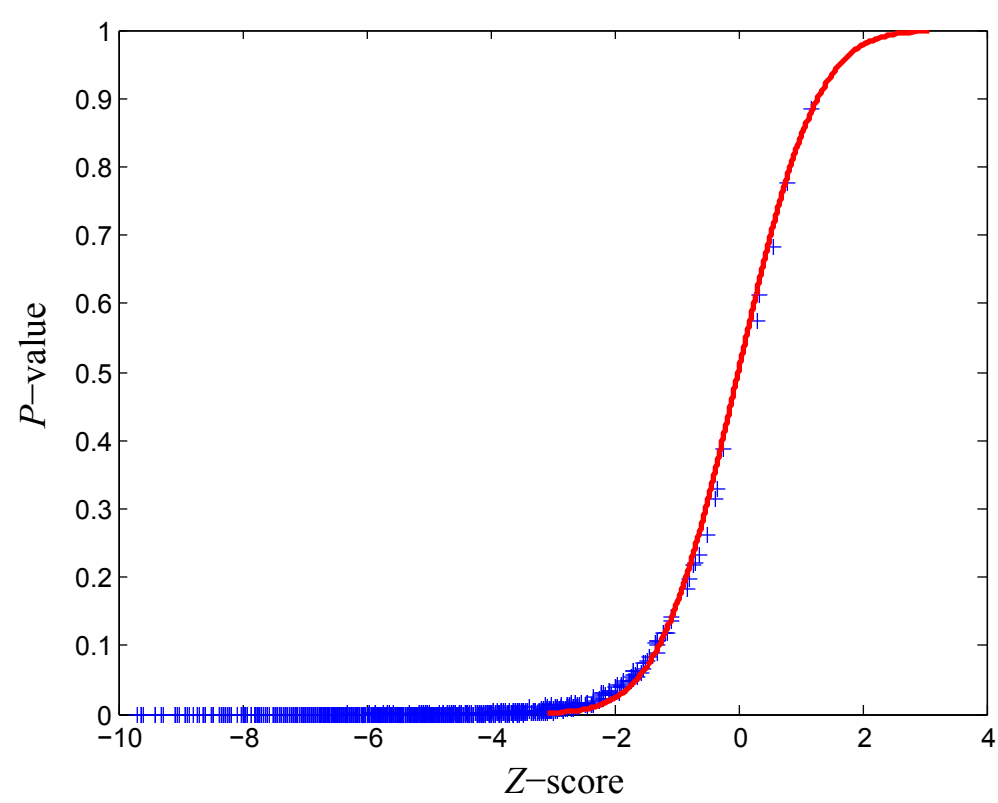

(c)

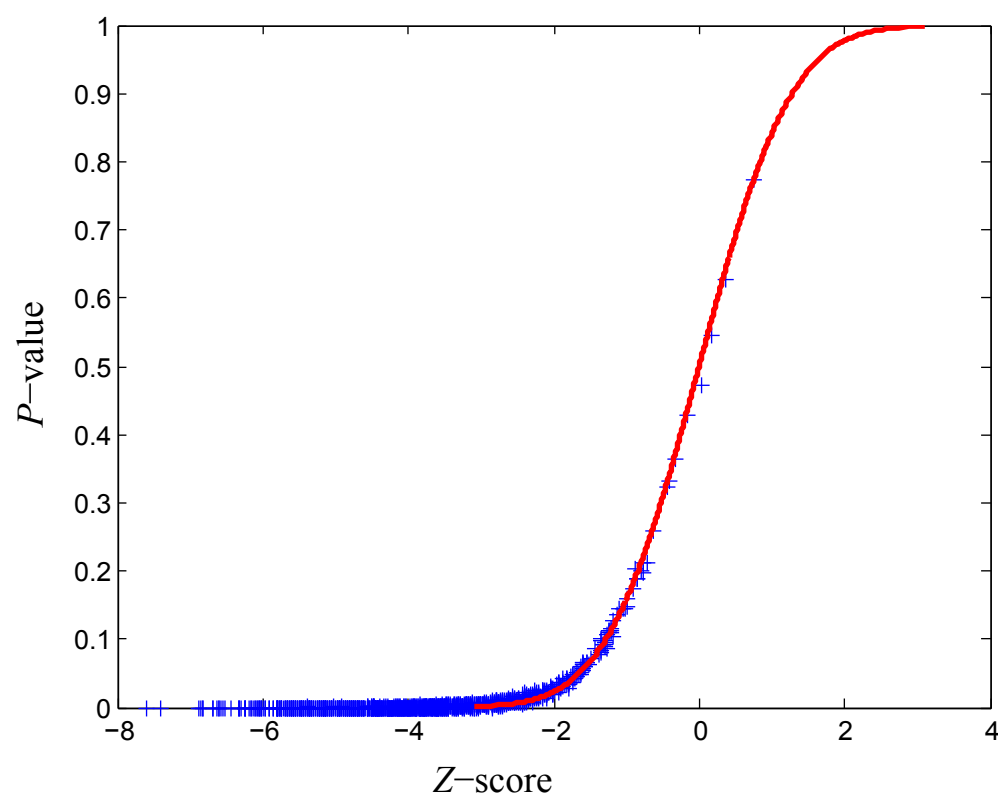

(d)

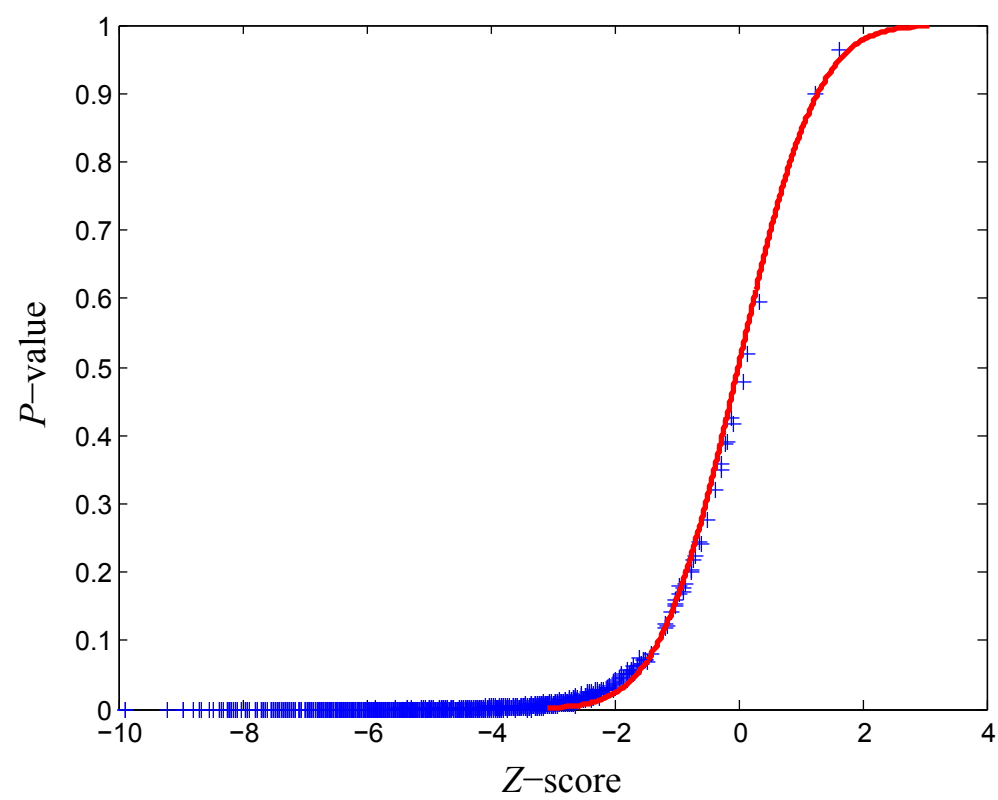

Supplement: Additional File 9 — Correlation between Z-scores and P-values of environmental robustness for all the 1,082 miRNAs. For each real pre-miRNA, the free energy is compared with that of 1,000 zero-markov shuffling pseudo pre-miRNAs (a), monoshuffling pseudo pre-miRNAs (b), first-markov shuffling pseudo pre-miRNAs (c) and dishuffling pseudo pre-miRNAs (d). The curve for a normal distribution of mean 0 and a standard deviation of 1 is also displayed. [file 1471-2148-7-223-S9.pdf]

(a)

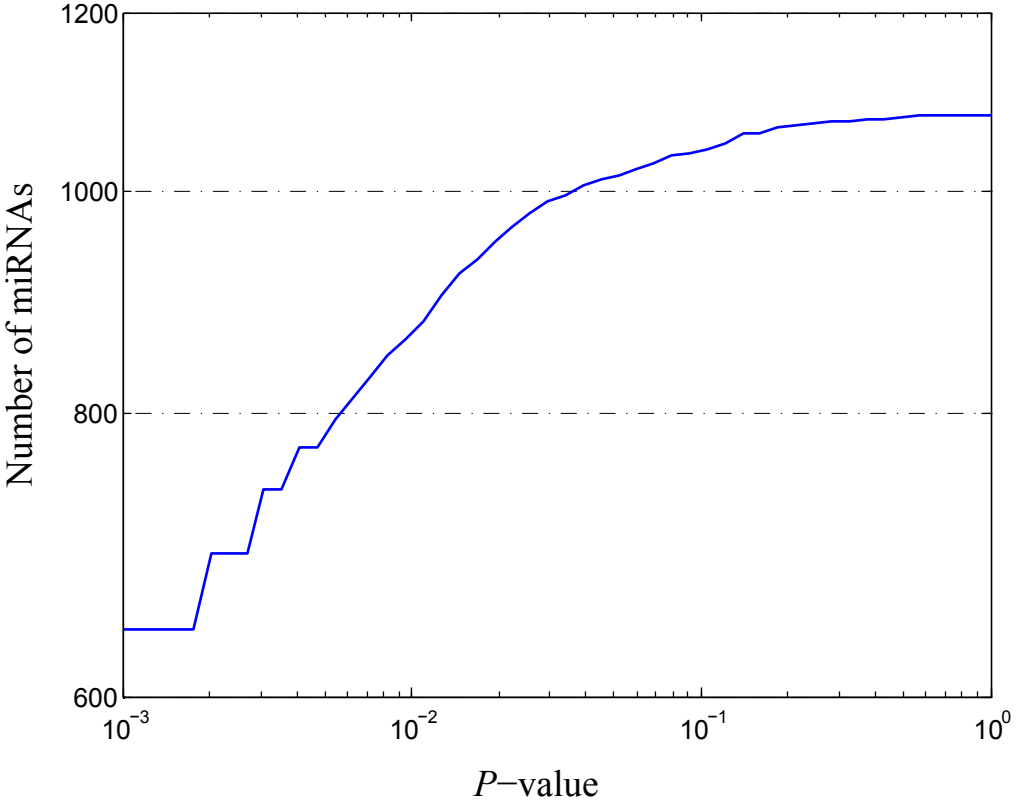

(b)

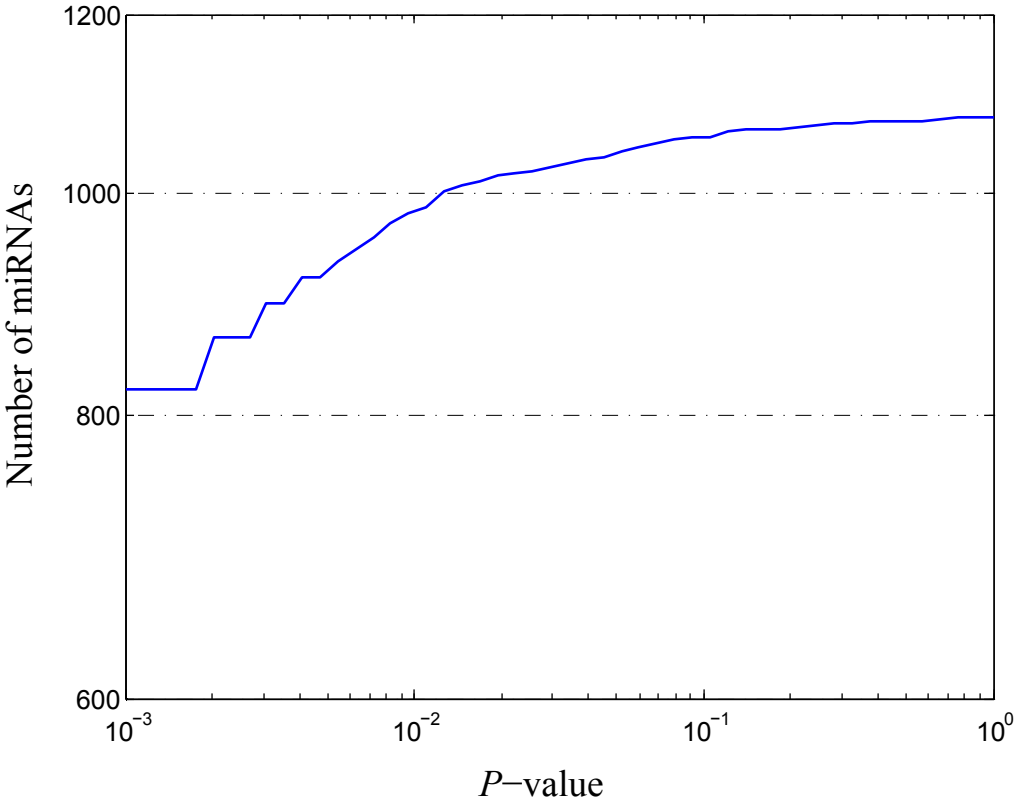

(c)

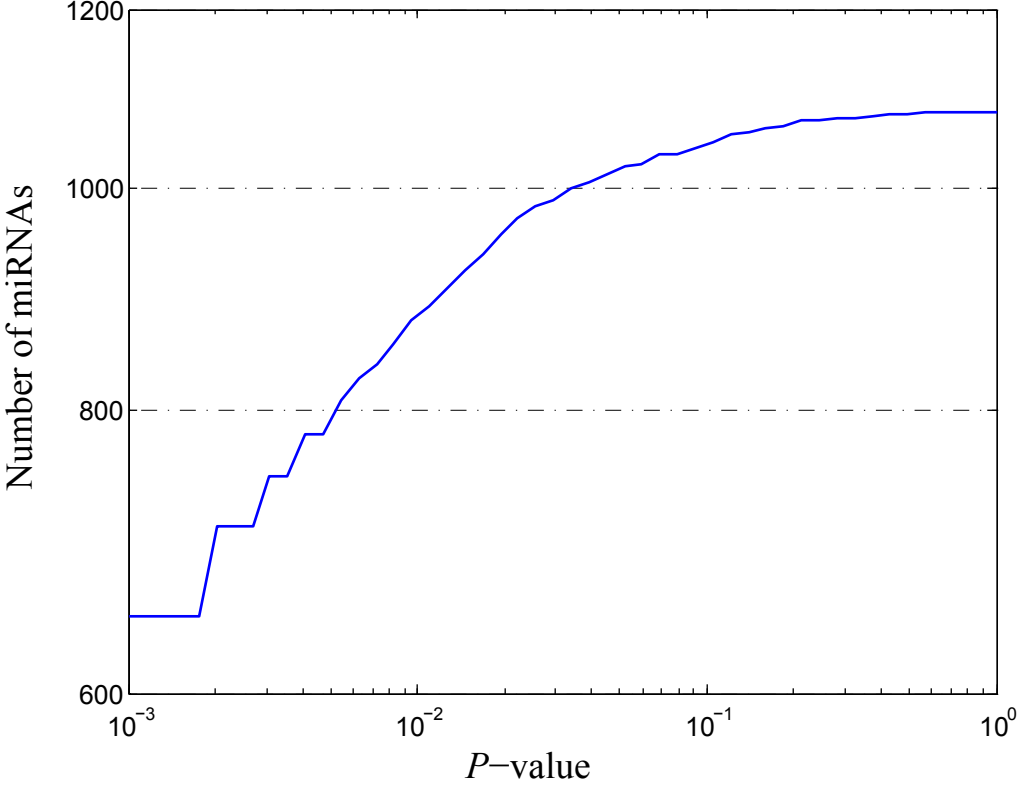

(d)

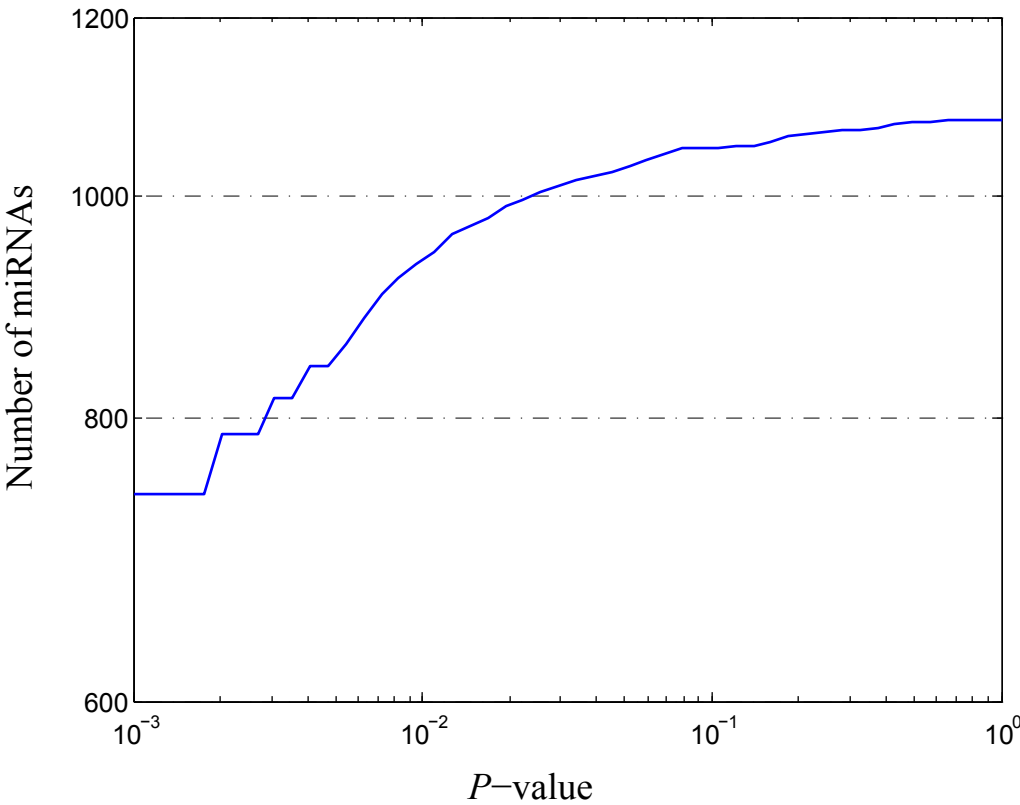

Supplement: Additional File 10 — P-value distributions of environmental robustness for the 1,082 miRNAs. For each real pre-miRNA, the free energy is compared with that of 1,000 zero-markov shuffling pseudo pre-miRNAs (a), monoshuffling pseudo pre-miRNAs (b), first-markov shuffling pseudo pre-miRNAs (c) and dishuffling pseudo pre-miRNAs (d). [file 1471-2148-7-223-S10.pdf]

(a)

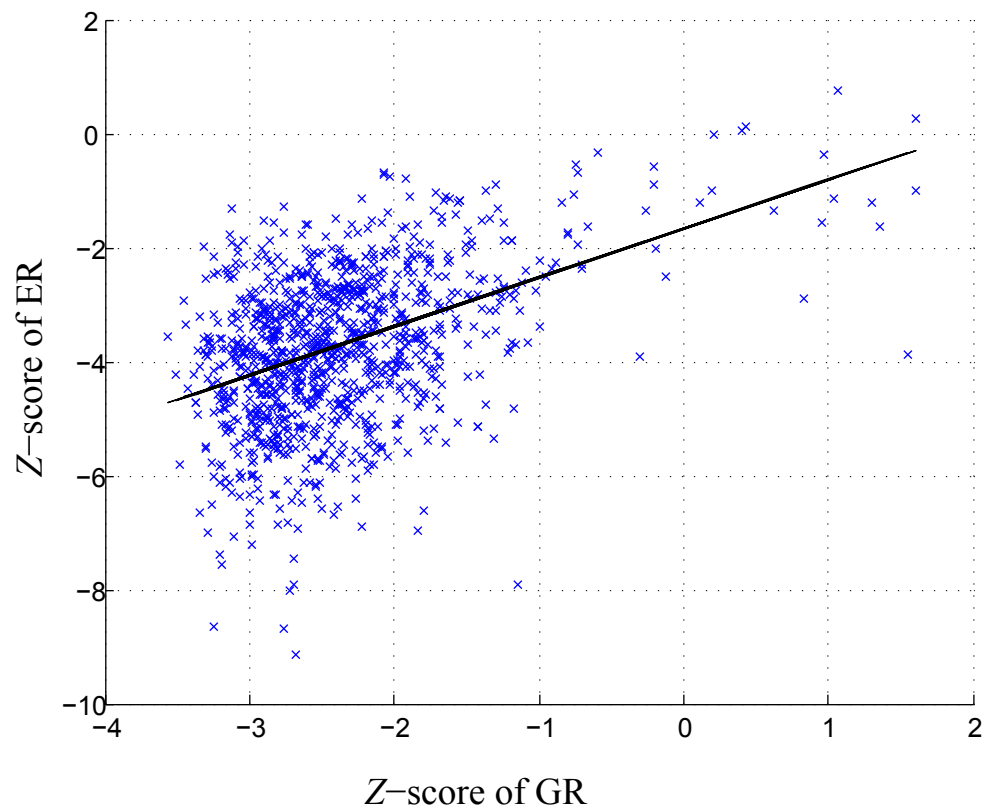

(b)

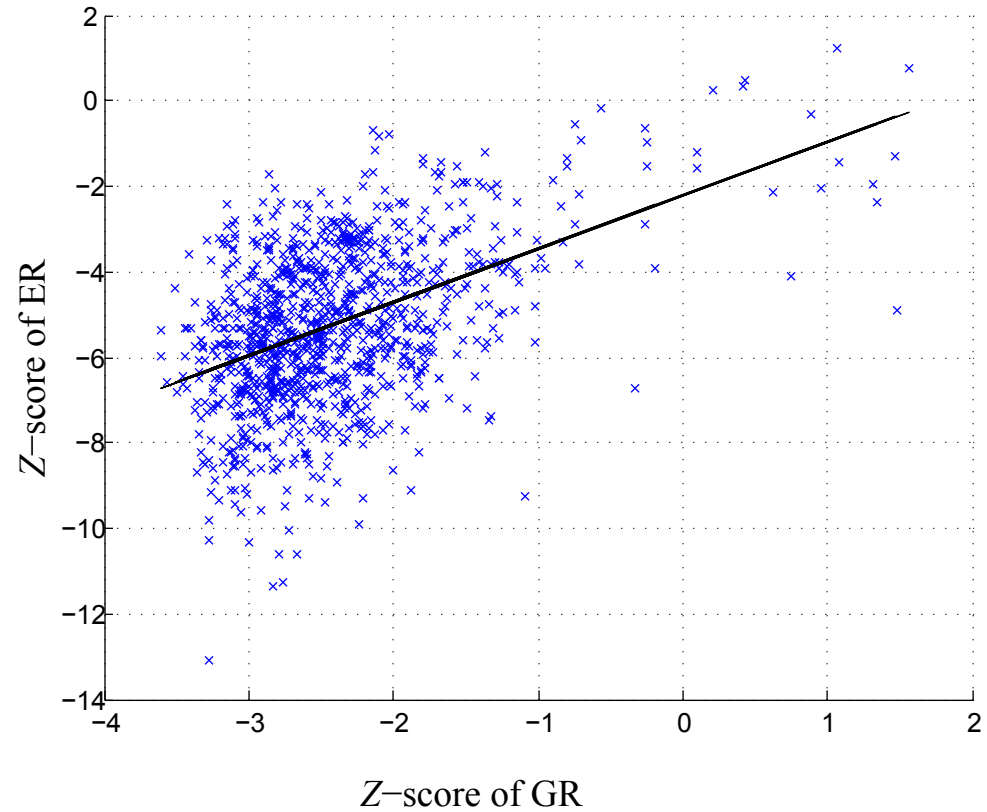

(c)

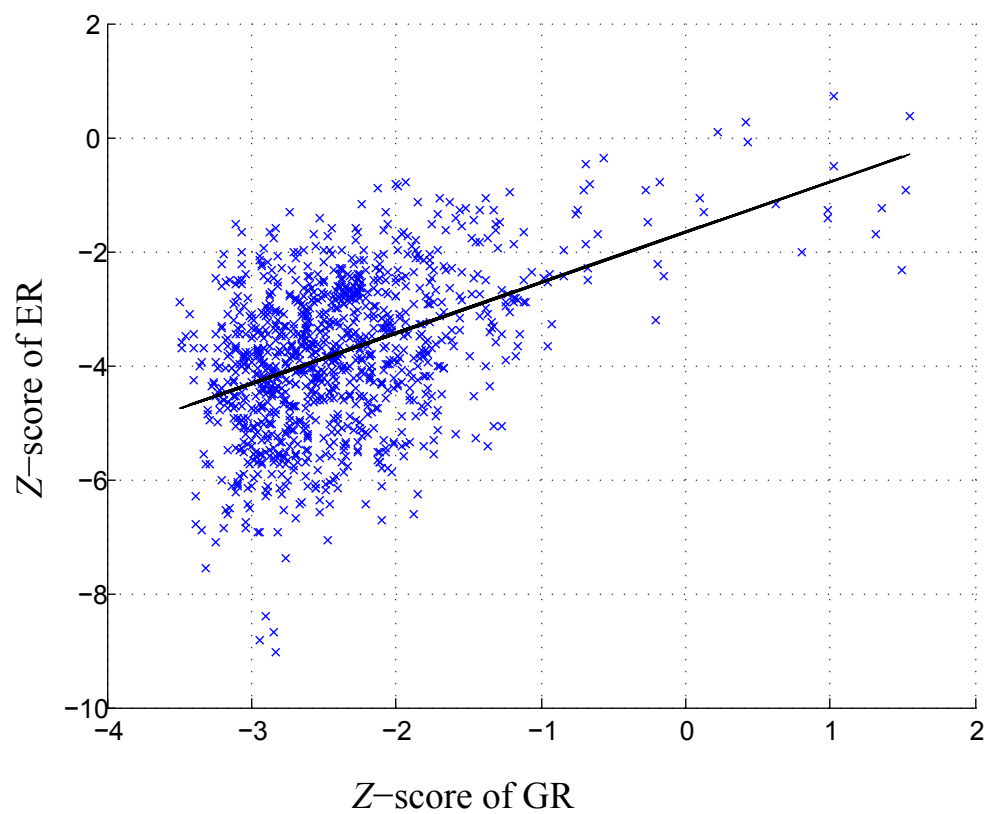

(d)

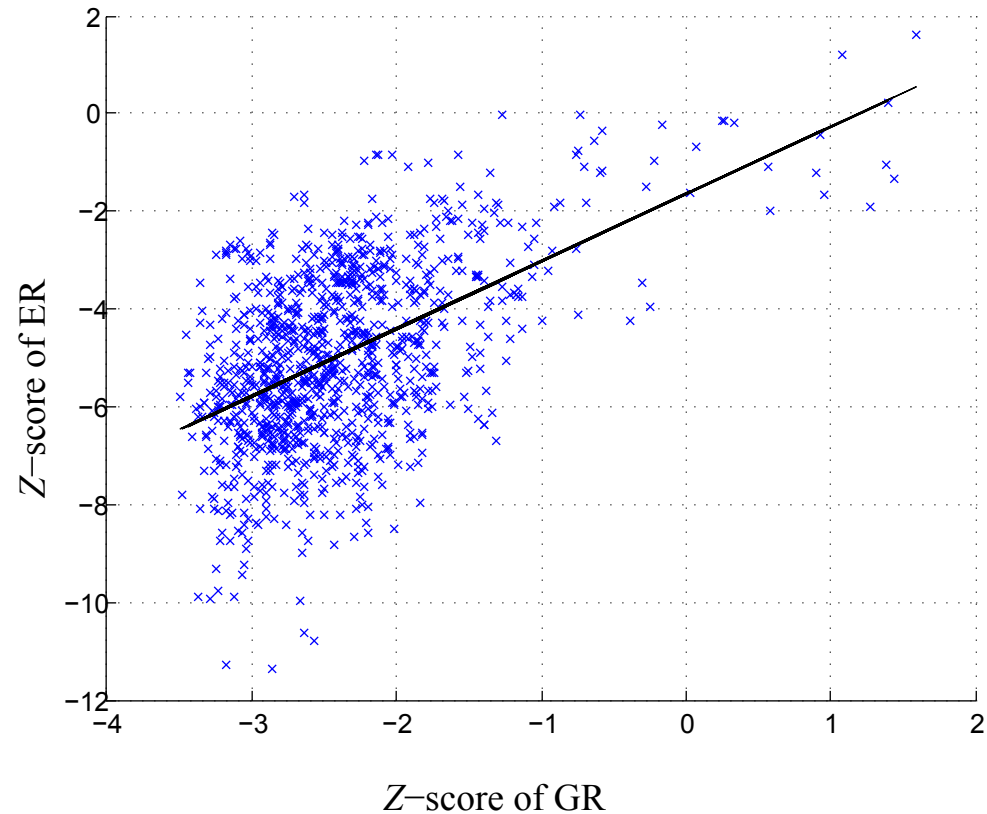

Supplement: Additional File 11 — Correlation between genetic and environmental robustness. Scatter plots of Z-scores of genetic robustness and environmental robustness. The Z-scores were obtained by comparing the robustness of real pre-miRNAs with that of 1,000 zero-markov shuffling sequences (a), monoshuffling sequences (b), first-markov shuffling sequences (c) and dishuffling sequences (d). [file 1471-2148-7-223-S11.pdf]

(a)

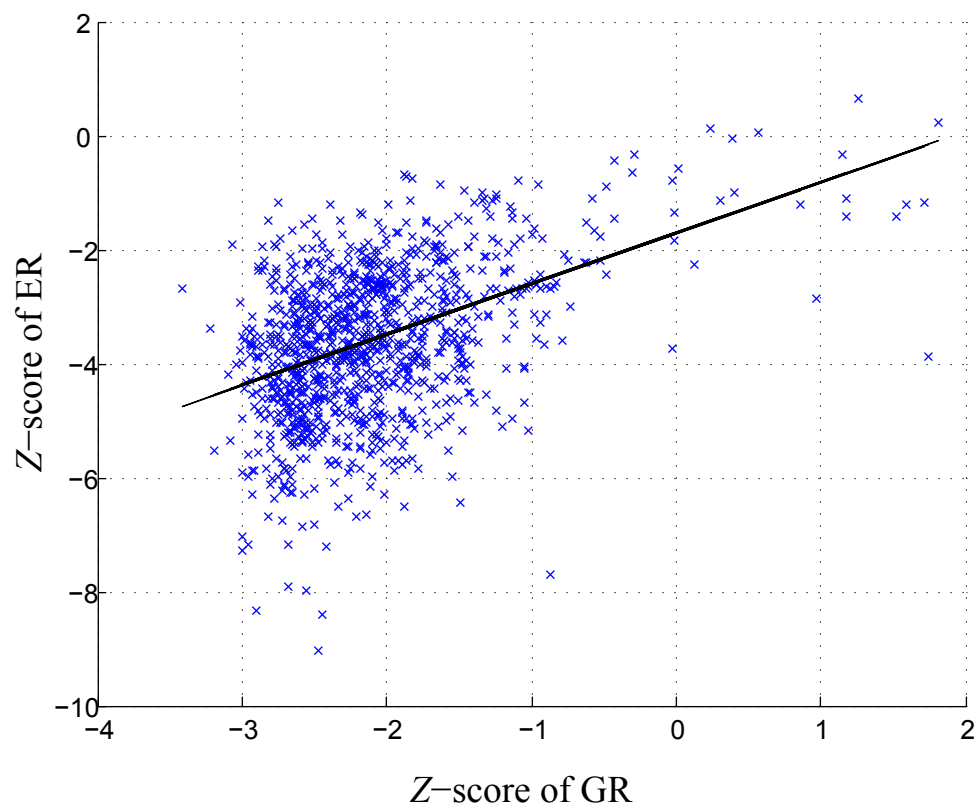

(b)

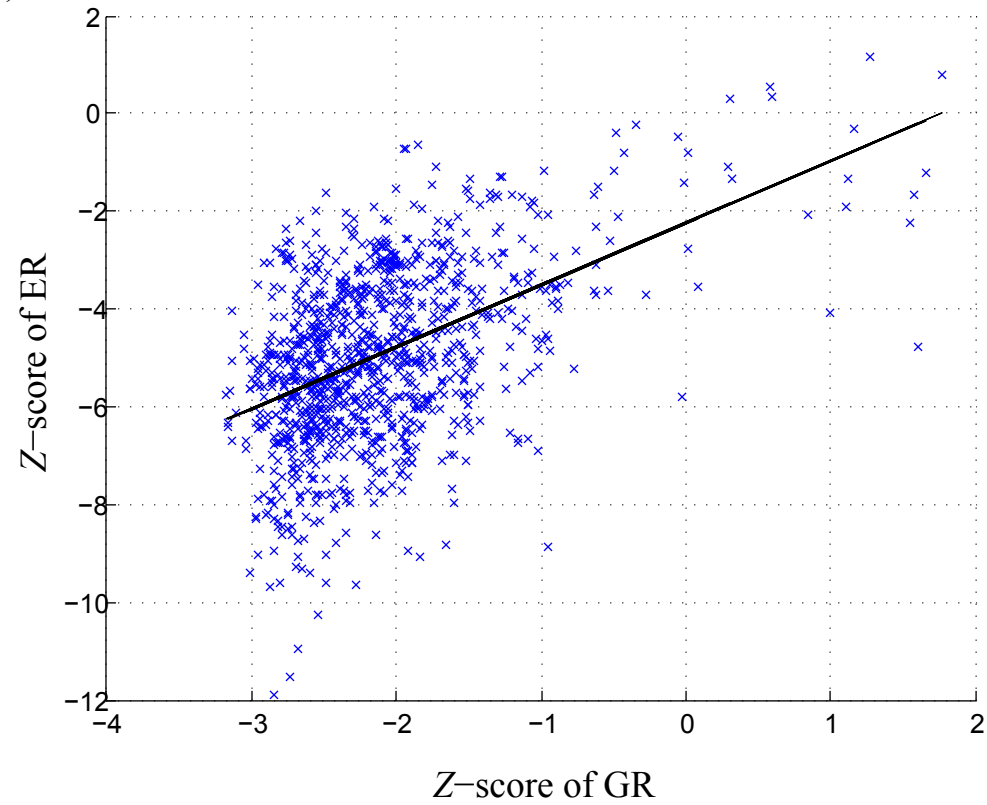

(c)

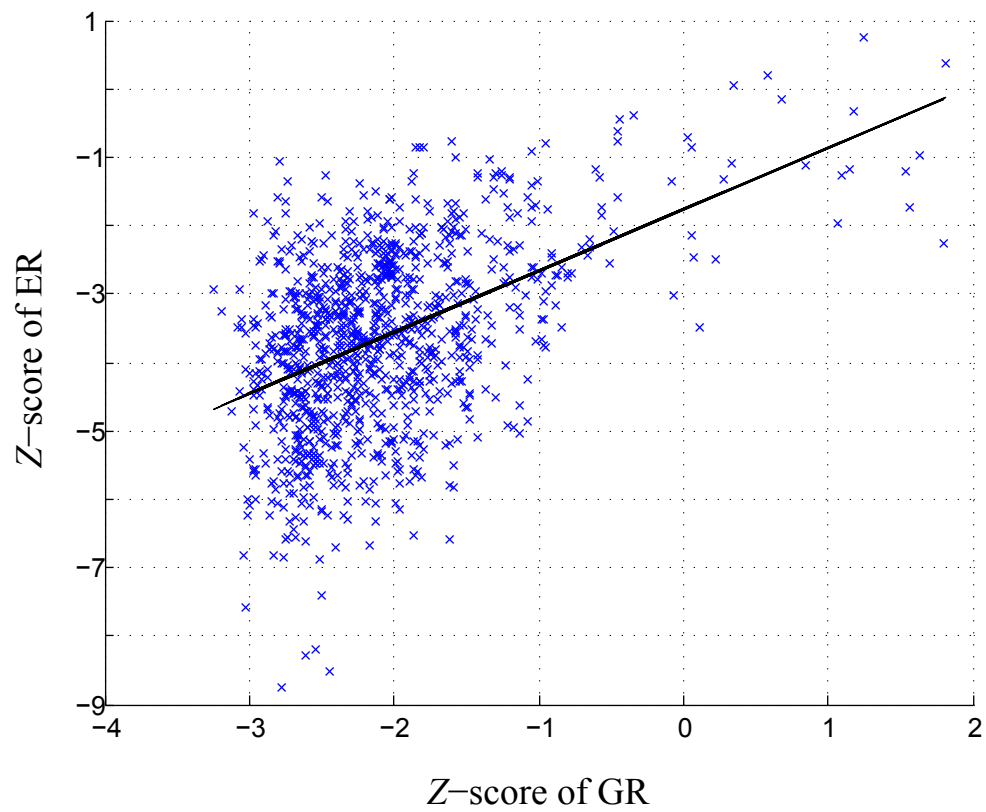

(d)

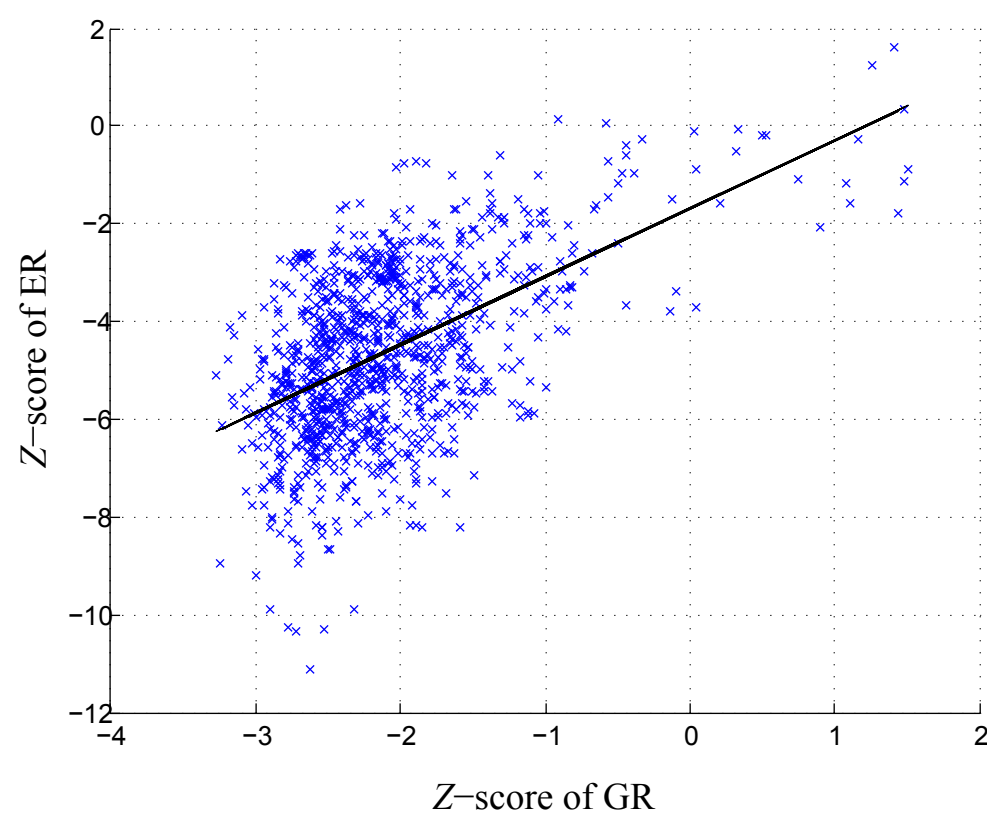

Supplement: Additional File 12 — Correlation between genetic and environmental robustness. Scatter plots of Z-scores of genetic robustness and environmental robustness. The Z-scores were obtained by comparing the robustness of real pre-miRNAs with that of 1,000 zero-markov shuffling pseudo pre-miRNAs (a), monoshuffling pseudo pre-miRNAs (b), first-markov shuffling pseudo pre-miRNAs (c) and dishuffling pseudo pre-miRNAs (d). [file 1471-2148-7-223-S12.pdf]

(a)

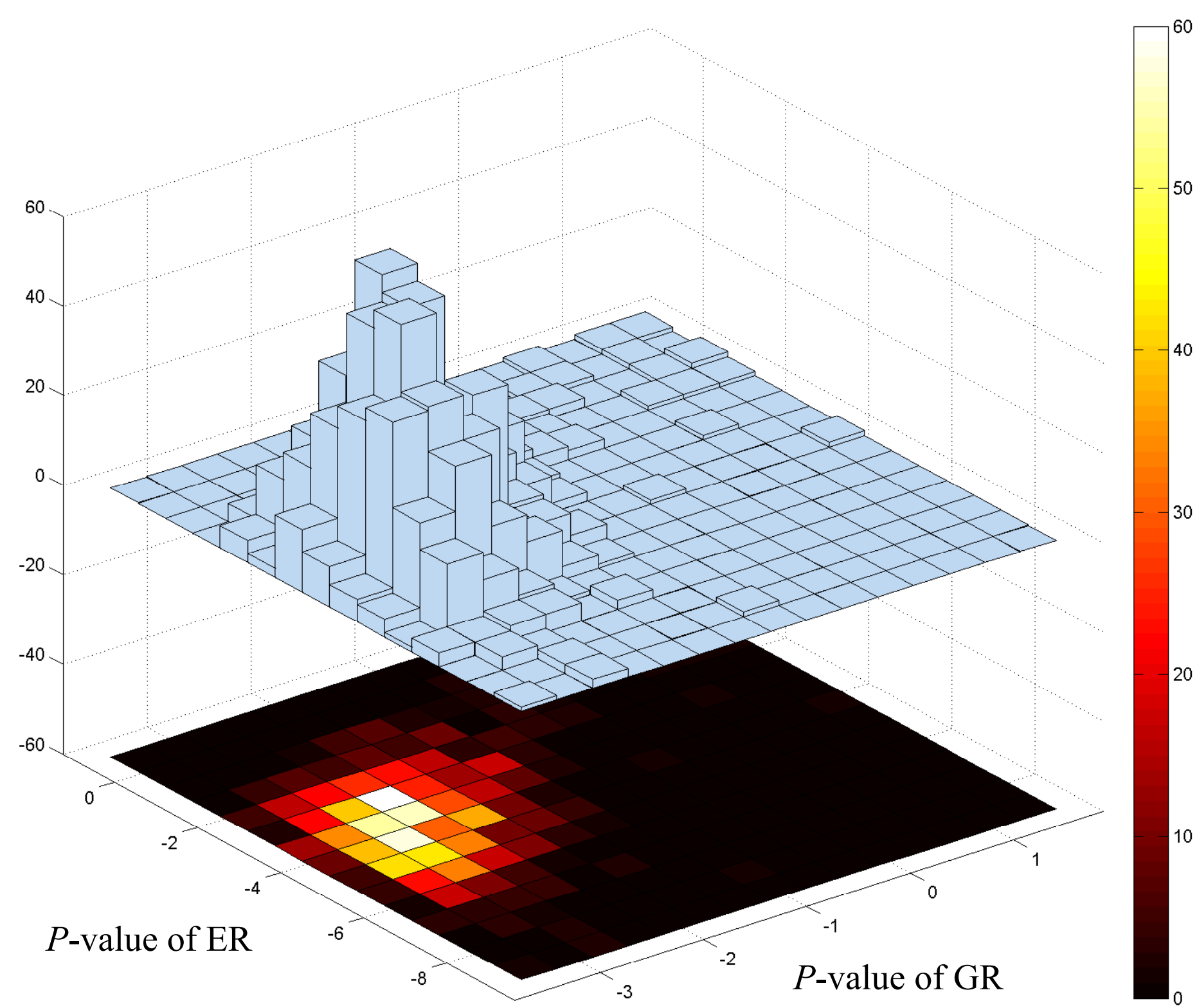

(b)

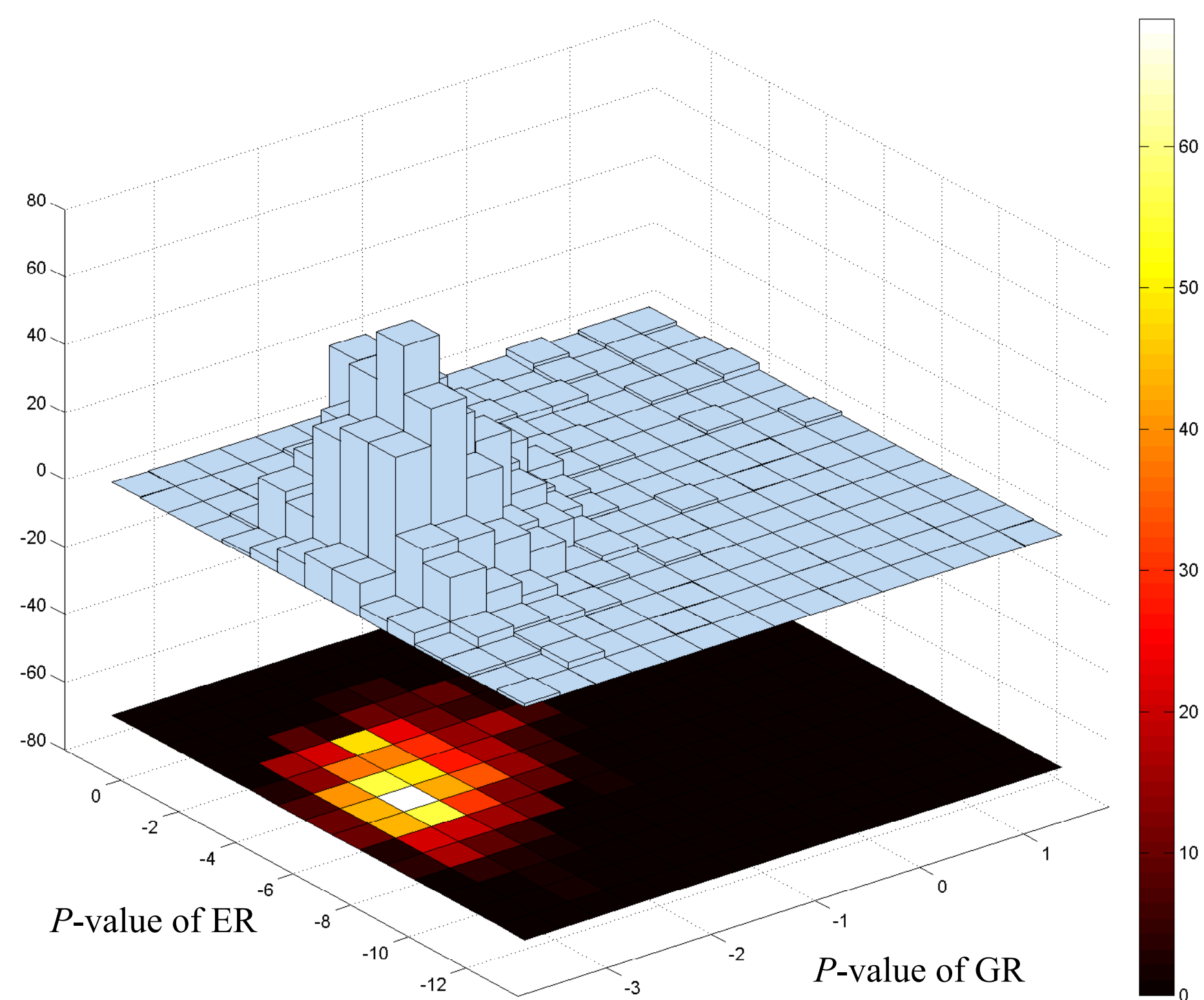

(c)

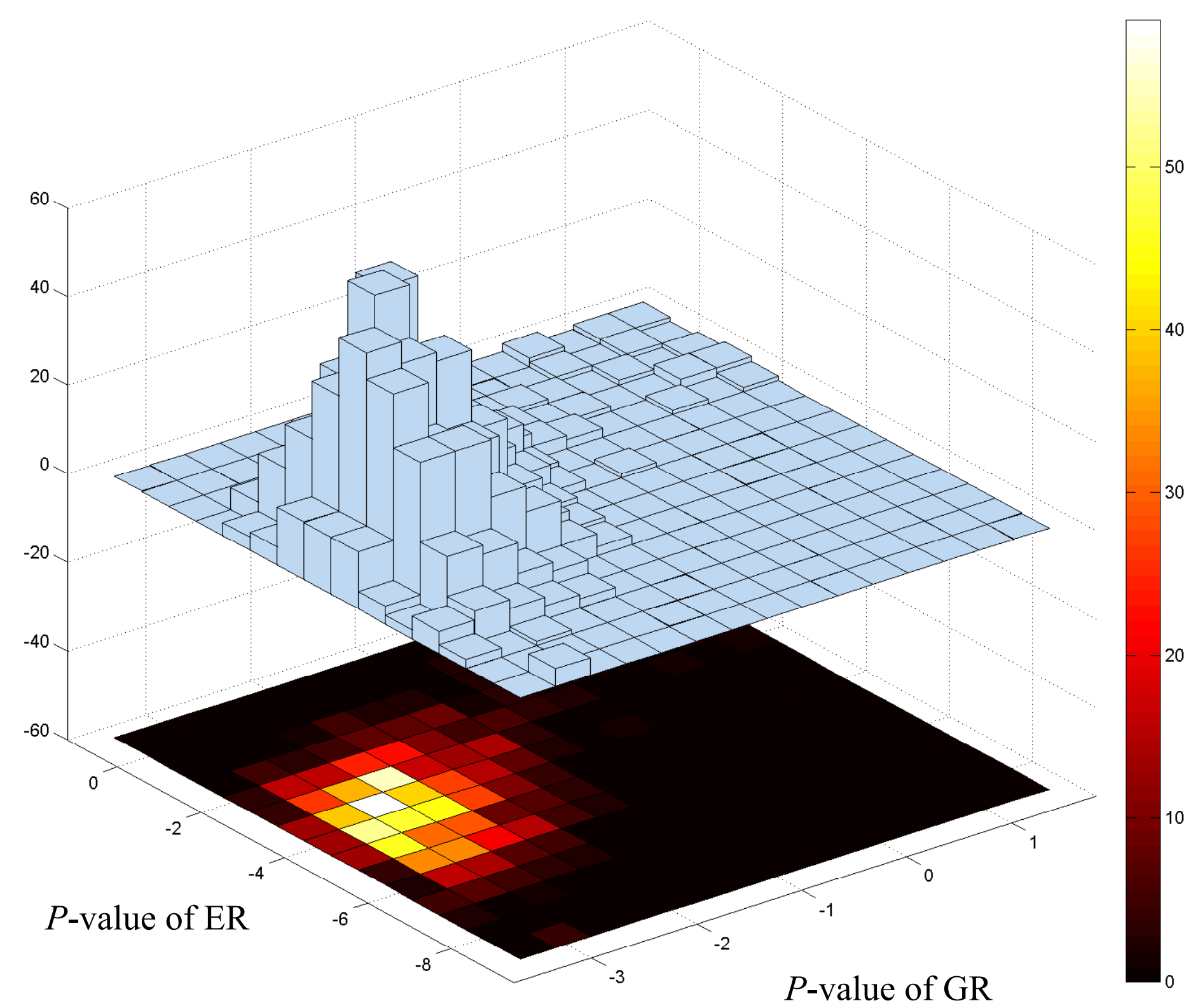

(d)

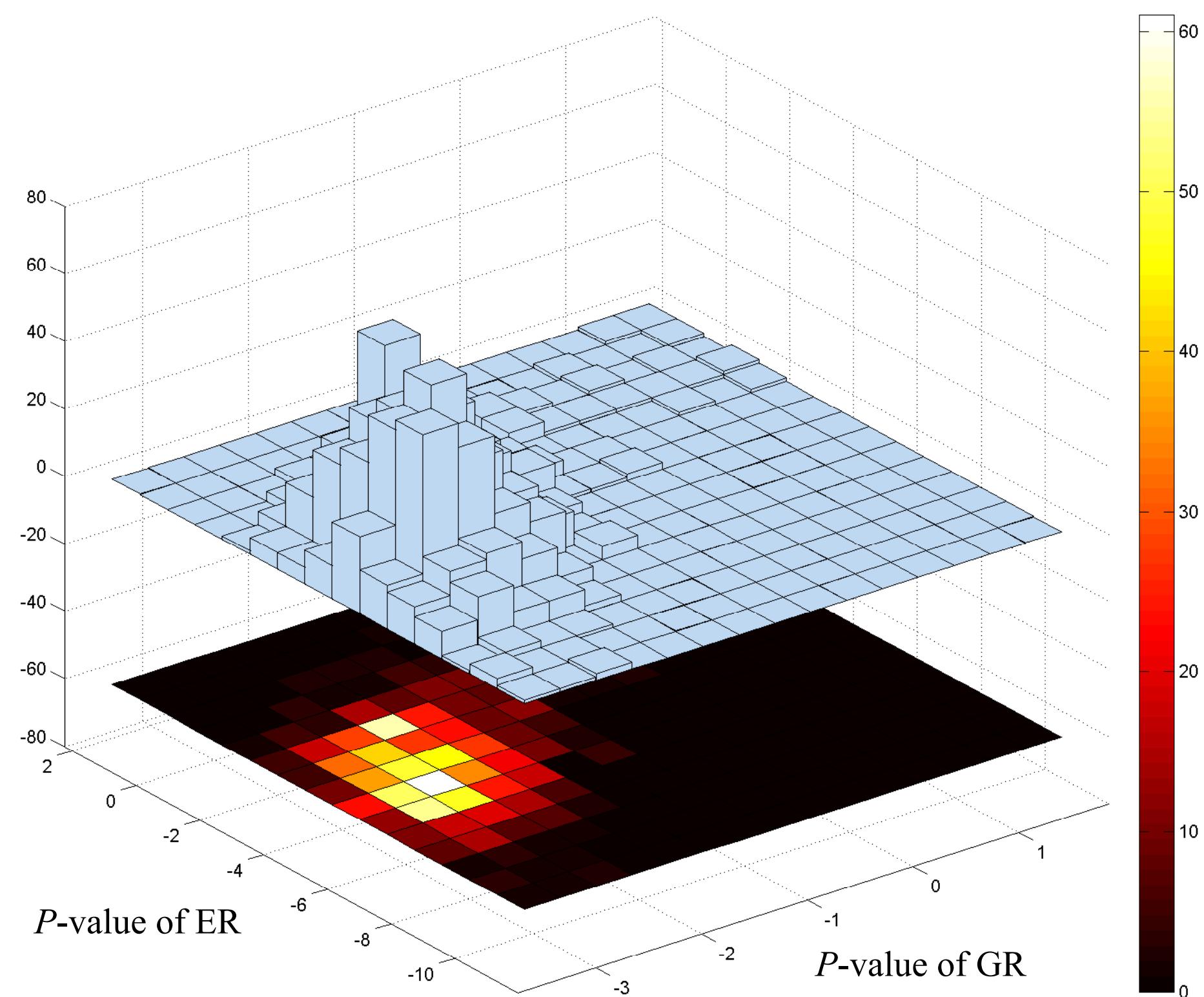

Supplement: Additional File 13 — Distribution of Z-score of genetic and environmental robustness. 3D histogram plots of Z-scores of genetic robustness and environmental robustness. The Z-scores were obtained by comparing the robustness of real pre-miRNAs with that of 1,000 zero-markov shuffling sequences (a), monoshuffling sequences (b), first-markov shuffling sequences (c) and dishuffling sequences (d). [file 1471-2148-7-223-S13.pdf]

(a)

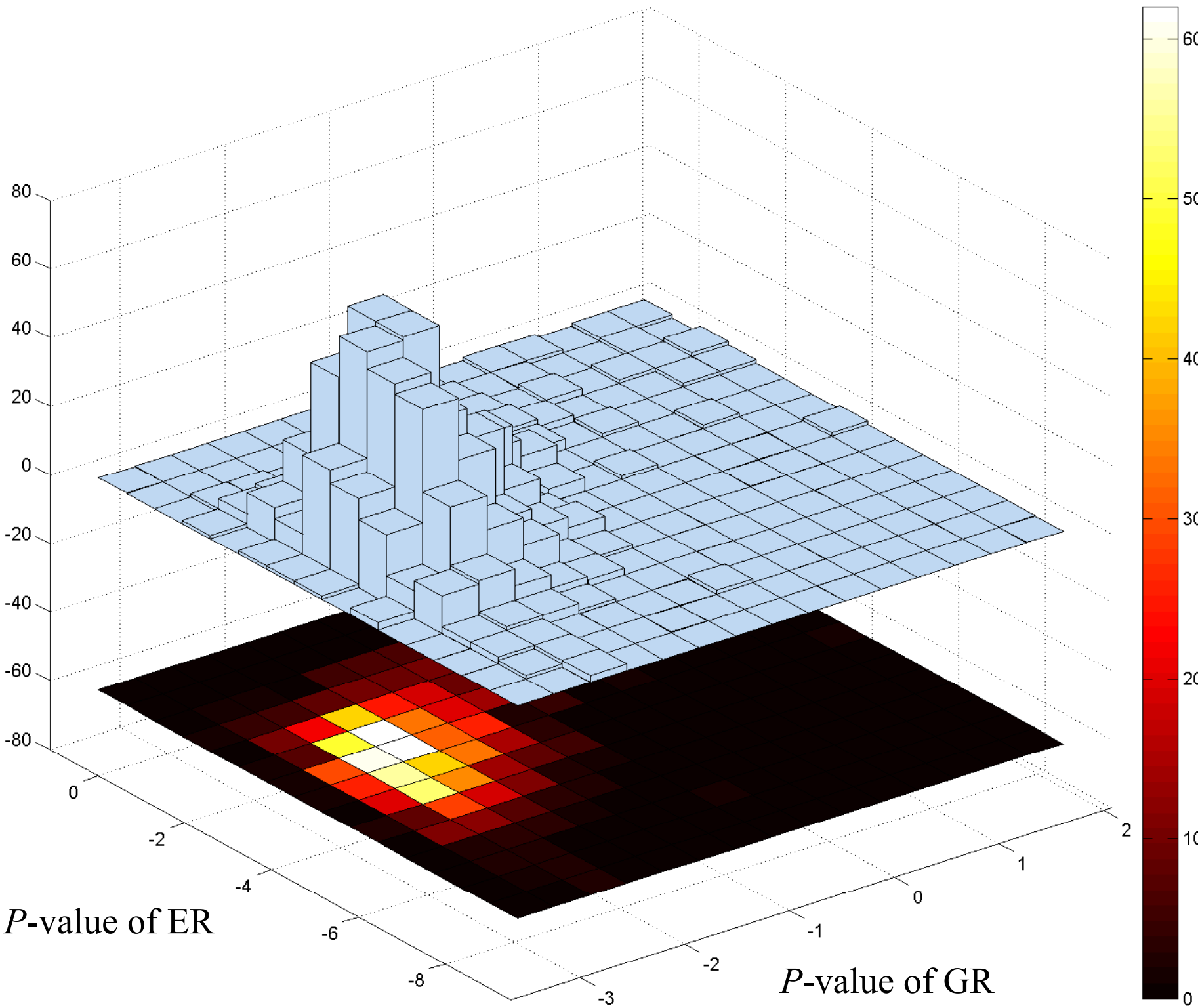

(b)

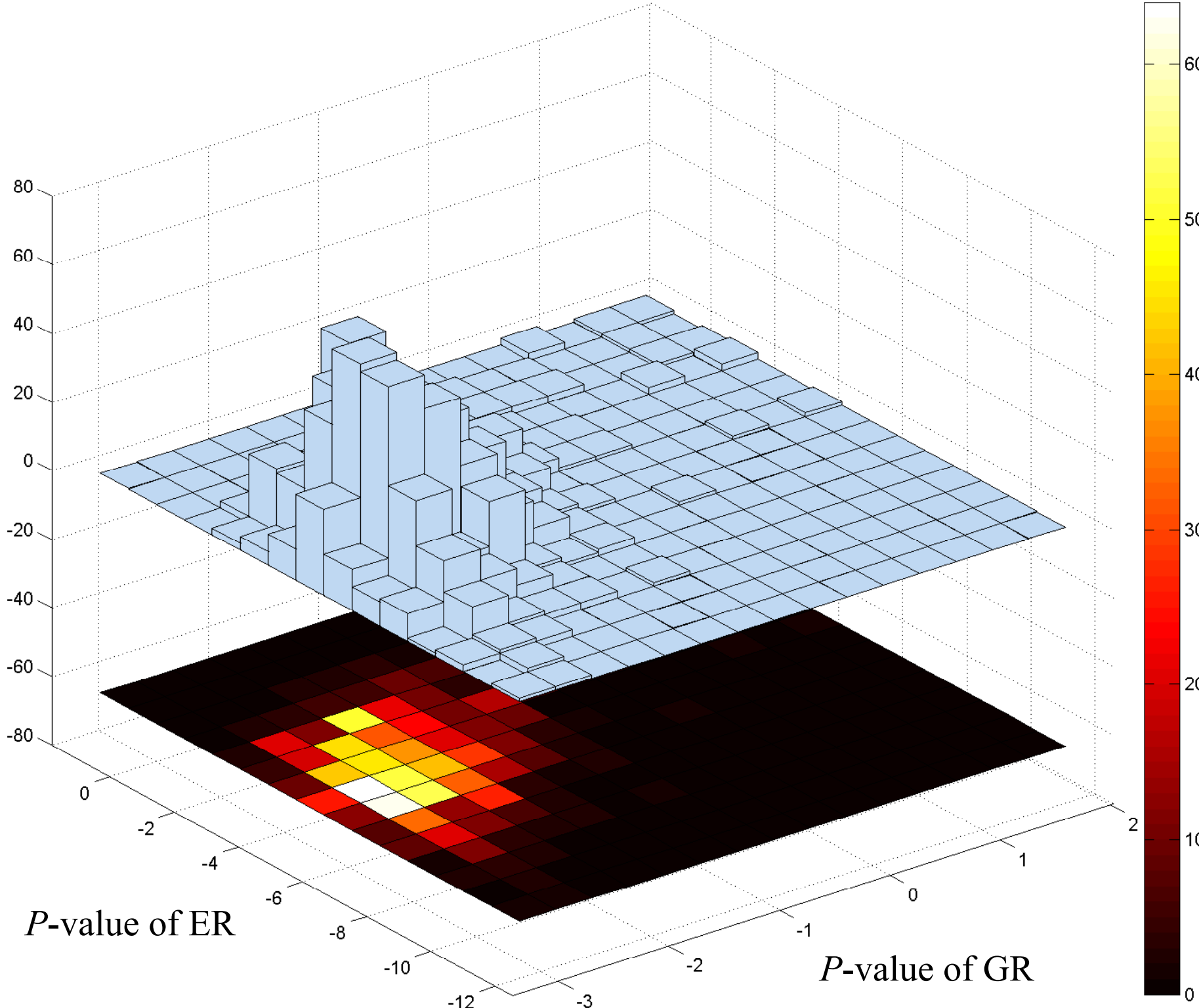

(c)

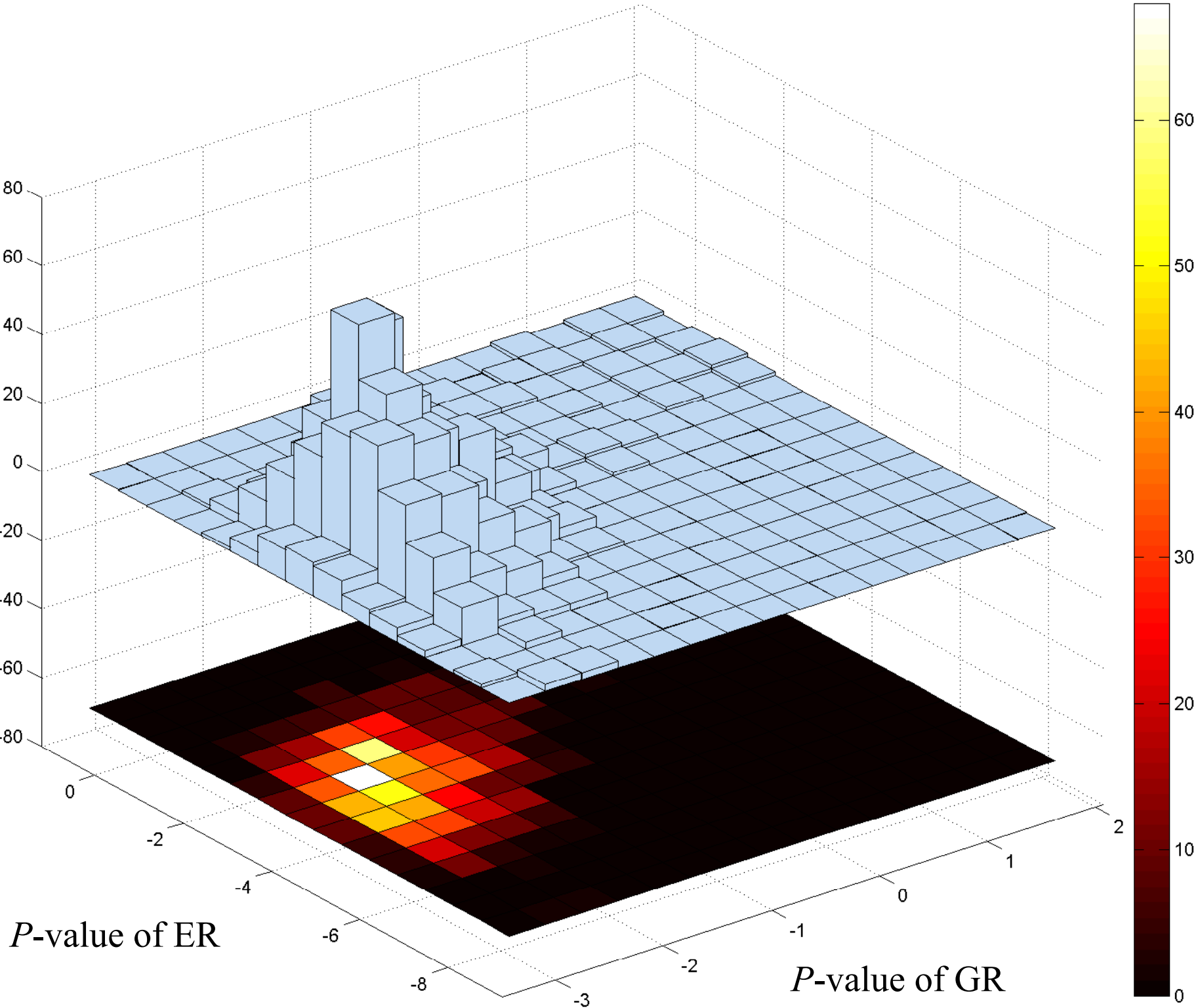

(d)

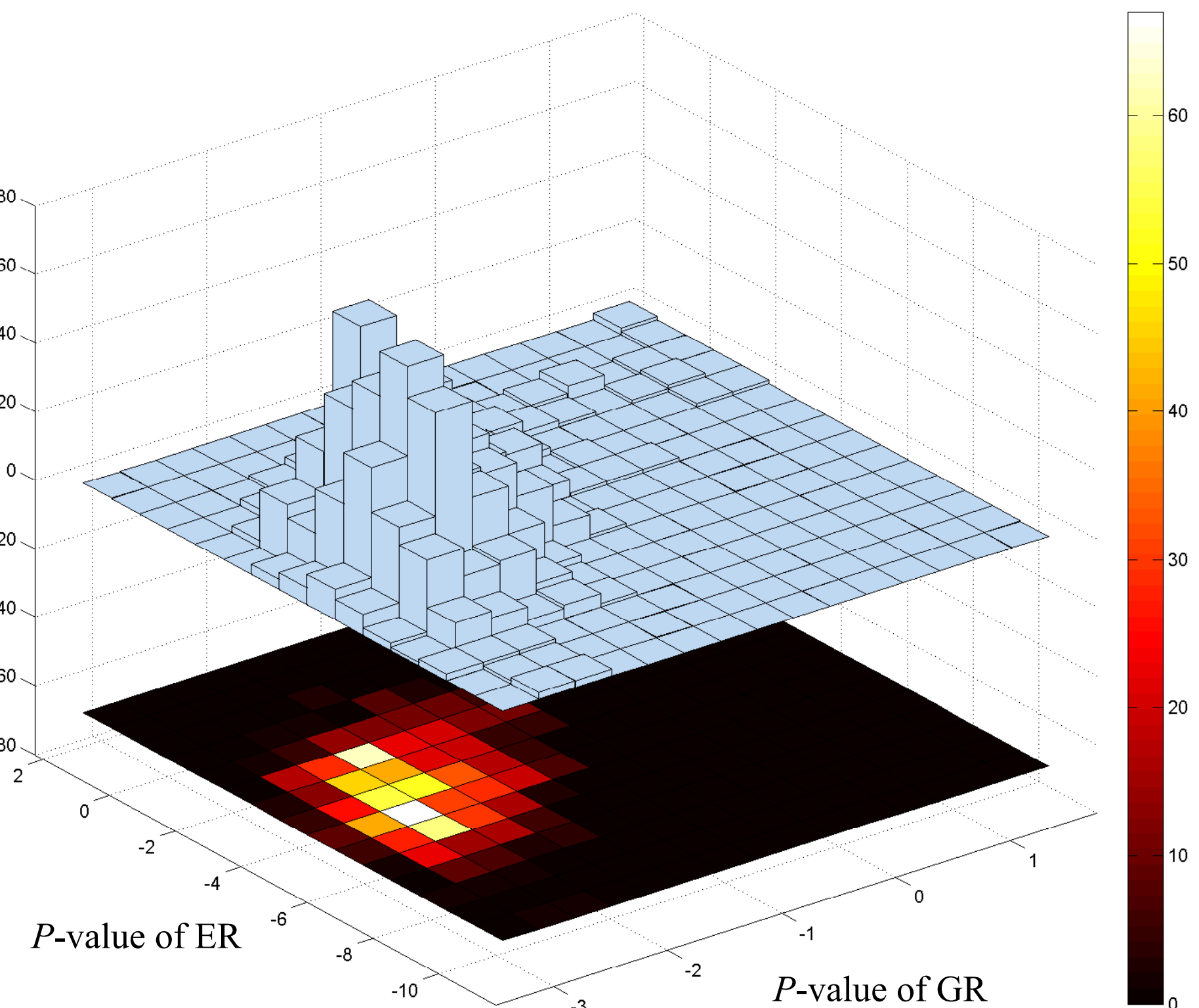

Supplement: Additional File 14 — Distribution of Z-score of genetic and environmental robustness. 3D histogram plots of Z-scores of genetic robustness and environmental robustness. The Z-scores were obtained by comparing the robustness of real pre-miRNAs with that of 1,000 zero-markov shuffling pseudo pre-miRNAs (a), monoshuffling pseudo pre-miRNAs (b), first-markov shuffling pseudo pre-miRNAs (c) and dishuffling pseudo pre-miRNAs (d). [file 1471-2148-7-223-S14.pdf]
